# The Role of Pigments in Light Color Variation of the Firefly *Photinus pyralis*

**DOI:** 10.1002/ece3.71927

**Published:** 2025-08-19

**Authors:** M. S. Popecki, S. A. Archer‐Hartmann, P. Azadi, R. L. Rogers, J. P. Wares, K. F. Stanger‐Hall

**Affiliations:** ^1^ Department of Genetics University of Georgia Athens Georgia USA; ^2^ Department of Bioinformatics and Genomics UNC Charlotte Charlotte North Carolina USA; ^3^ Complex Carbohydrate Research Center University of Georgia Athens Georgia USA; ^4^ Odum School of Ecology University of Georgia Athens Georgia USA; ^5^ Department of Plant Biology University of Georgia Athens Georgia USA

**Keywords:** fireflies, gene expression, pigment, signal evolution

## Abstract

Firefly light color does not appear to directly influence mate choice, but it seems to be under selection to enhance signal detectability by increasing contrast with the visual background. While luciferase has been considered the sole determinant of light color, populations of the Big Dipper firefly ( *Photinus pyralis* ) with identical luciferases display variation in emitted light color. Here, we examined whether *P. pyralis* fireflies use pigments to filter the light produced by luciferase and contribute to variation in light color across populations. If pigments influence light color, we predicted that genes involved with pigment biosynthesis would be expressed in light organs, and that pigment substrates could also be detected in firefly light organs. Since screening pigments are important for insect vision, we examined whether any pigment genes and/or transporters expressed in light organs were also expressed in eyes, thus providing a direct pigment‐based mechanism for the reported tuning between emitted light color (light organ) and visual sensitivity (eyes). We identified 46 ommochrome and pterin pigment genes expressed in *P. pyralis* light organs and eyes, including those whose products could filter the light generated by luciferase and influence emitted light color. The shared pigments between light organs and eyes offer candidates for signal tuning. While we found no statistically significant differences between the pigment gene expression of *P. pyralis* fireflies in populations with yellower and greener signals, our data suggest several mechanisms for how pigments in the light organ could modify *P. pyralis* signal color.

## Graphical abstract

We tested the novel hypothesis that genes encoding enzymes and transporters in pigment pathways (“pigment genes”) produce pigments in the firefly light organ that filter bioluminescent light, tuning the color of emitted light signals to match visual sensitivity. We detected the gene expression of 46 ommochrome and pterin pigment genes in *P. pyralis* light organs, including those whose products could filter the light generated by luciferase and influence emitted light color. The presence of pigments in both light organs and eyes could serve as a mechanism for the known tuning between firefly light spectra and visual sensitivity. While we found no statistically significant differences between the pigment gene expression of *P. pyralis* fireflies with yellower and greener signals, our data suggest several mechanisms by which pigments in *P. pyralis* light organs could modify the signal color of *P. pyralis* fireflies. Image © Radim Schreiber/FireflyExperience.org, used with permission.

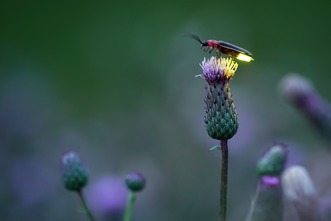

## 1 Introduction

Fireflies (beetles in the family Lampyridae) are famous for their bioluminescent displays when searching for a mate. There are more than 150 firefly species in North America, and over 10 may display at a single location (Lloyd 1969). To recognize conspecifics and reduce mating mistakes, fireflies emit species‐specific flash patterns with a unique flash duration, inter‐flash intervals, and pattern repeat intervals (e.g., Stanger‐Hall and Lloyd 2015). There is also a diversity in light color across species, ranging from green to orange (550–579 nm; Hall et al. 2016). While light color may not play a direct role in mate choice (Lloyd 1966), it is crucial for signal detection (i.e., generating contrast with the visual background) and a prerequisite for mating success (Lall et al. 1980). The evolution of firefly light color has been postulated as a two‐step process for improving light signal detection in their environment (Seliger et al. 1982a, 1982b).

**FIGURE 1:**
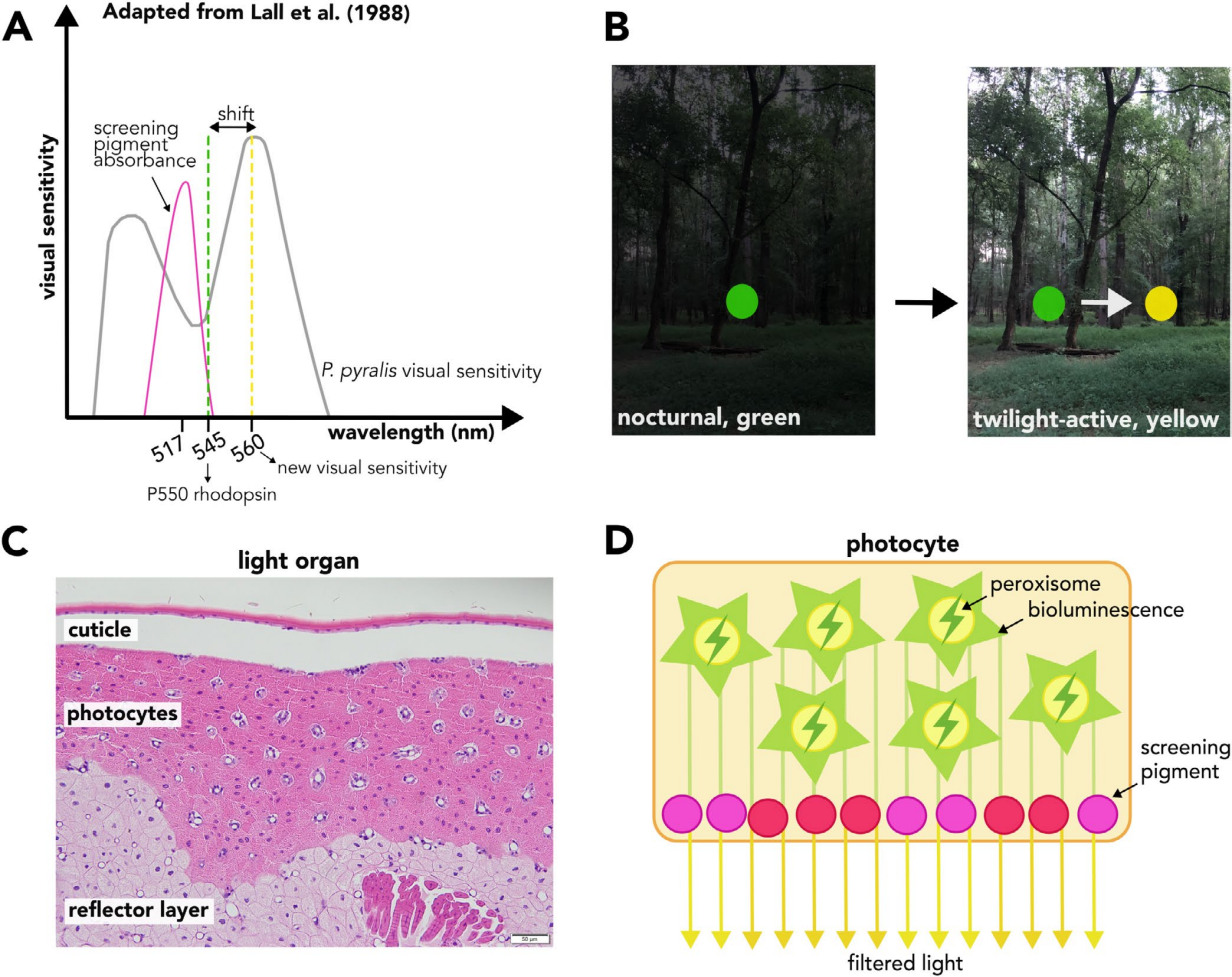
(A) Diagram of the shifted peak visual sensitivity in *P. pyralis* . An unknown screening pigment absorbs wavelengths at the green end of the incoming light spectrum (Lall et al. 1988; Cronin et al. 2000), resulting in relatively more yellow light stimulating the P550 rhodopsin (with LW opsins: Sander and Hall 2015), which maximally absorbs photons at 545 nm (green). As a result, the peak visual sensitivity has narrowed from a broad green/yellow to a narrow, yellow‐shifted peak sensitivity (modified from Lall et al. 1988). (B) At twilight, yellow or orange signals are more conspicuous than green signals as they have increased contrast with background foliage. (C) Microscopic image of the *P. pyralis* light organ. The reflective layer (dorsal) is composed primarily of uric acid crystals which amplify signal intensity and direct bioluminesence outward through the clear cuticle (ventral). Bioluminescence is produced within photocytes. (D) Depiction of a photic cell within the light organ; bioluminescence is produced in peroxisomes by luciferase. We propose that light passes through pigment granules in the photic cells and absorbs wavelengths at the greener end of the spectrum, shifting the emitted light towards yellow.

Ancestral fireflies likely emitted green bioluminescence during their nocturnal displays (Oba et al. 2020), matching the peak green sensitivity of insect eyes (Briscoe and Chittka 2001). However, when some fireflies (i.e., many species in the genus *Photinus*) shifted their activity time from dark to twilight, possibly due to predation pressure from nocturnal *Photuris* fireflies (Lloyd 1973), green light signals became harder for their mates to detect among the now visible green vegetation. This likely introduced selective pressure on firefly eyes for enhanced detection of yellow wavelengths within their green light signals, resulting in a shifted peak visual sensitivity from green towards yellow (Seliger et al. 1982a, 1982b). Consequently, fireflies that emitted light spectra with relatively more yellow wavelengths had increased mating success, as they were more easily seen by potential mates (Figure 1). Such shifts in peak visual sensitivity towards more yellow light was followed by a corresponding shift in the signal color of different twilight‐active species, expanding their range of light color from green‐yellow to orange (Lall et al. 1980; Seliger et al. 1982a, 1982b; Hall et al. 2016).

Our study focuses on the second step, the evolution of firefly light color. Adult fireflies generate bioluminescence within their light organs (specialized abdominal segments with a clear cuticle) through catalysis of luciferin by the enzyme luciferase. While luciferin is conserved among beetles (Seliger et al. 1961; Seliger and McElroy 1964), luciferase exhibits structural diversity across fireflies (Hastings 1983). The amino acid sequence of luciferase determines enzymatic activity, and thus bioluminescent properties such as light color (Nakatsu et al. 2006) and intensity (Liu and Urban 2017), which can be altered by individual amino acid substitutions, as demonstrated by in vitro studies on luciferase (e.g., Fujii et al. 2007; Kajiyama and Nakano 1991; Branchini et al. 1999, 2017).

Under the traditional paradigm, luciferase is thought to be the sole determinant of bioluminescence color in fireflies; however, this was challenged by Lower et al. (2018), who documented that *Photinus pyralis* populations with identical luciferases differed in emitted light color. This raises the question: How is variation in firefly light color generated? Here, we propose and test the hypothesis that pigments in firefly light organs contribute to their emitted light color. If pigments can modify light color, we predict that (1) genes encoding enzymes and transporters in pigment biosynthesis pathways are expressed in light organs, and (2) pigment substrates can be detected in the light organ. Should this be supported, we expect that (3) pigments can modify firefly signal color by absorbing greener wavelengths from the light produced by luciferase, shifting the color of emitted light from green towards yellow. Thus, (4) fireflies with identical luciferases that differ in light color should display variation in both the expression of genes encoding pigments and pigment content (e.g., abundance, types) within their light organs.

**FIGURE 2:**
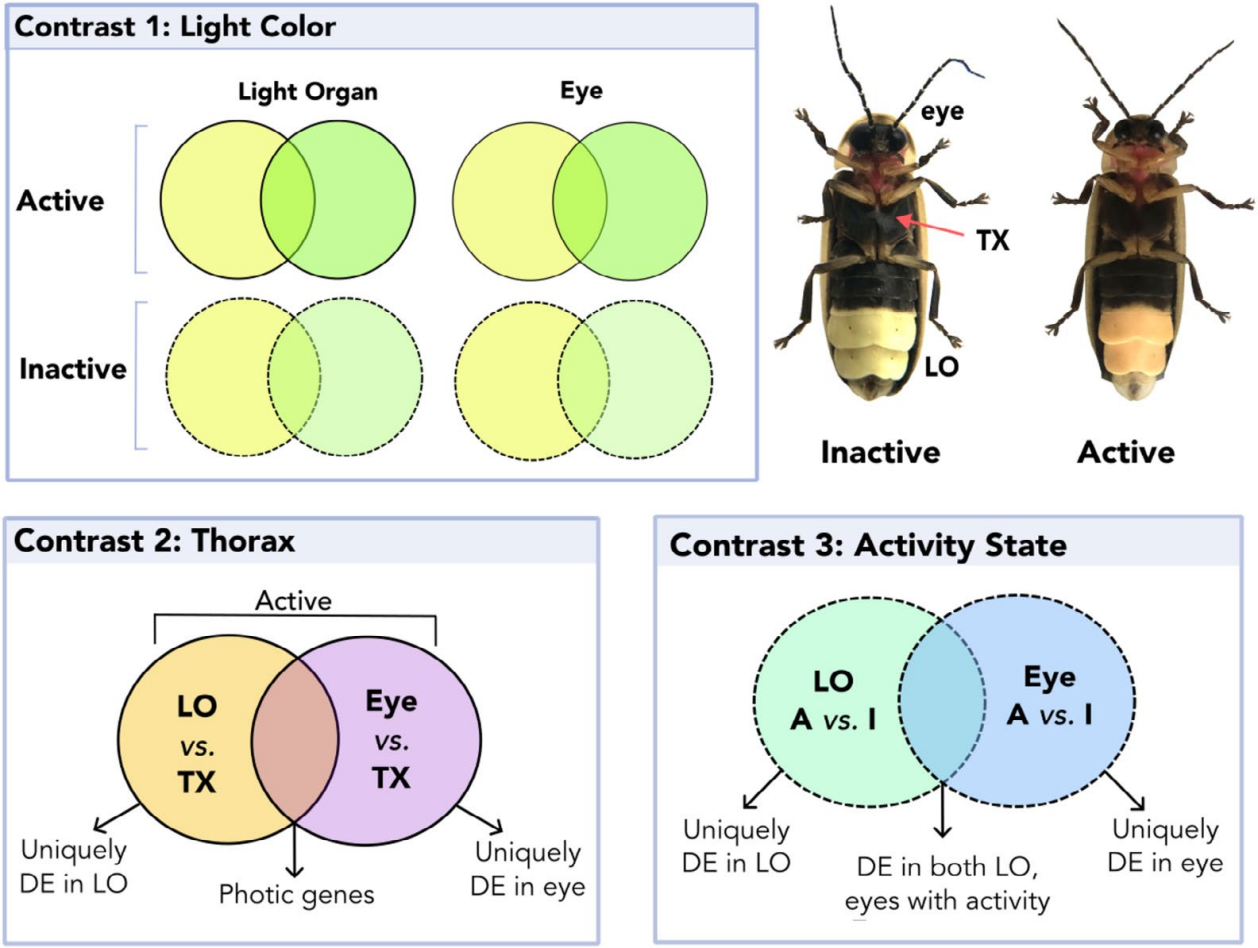
Overview of differential gene expression analyses. LO, light organ; TX, thorax. We compared expression patterns between tissues, activity state, and light color. The light organ of *P. pyralis* fireflies appears ivory‐yellow during the inactive state (left) and shifts to a yellow‐orange hue while fireflies are active (flying and signaling) at twilight (right).

Indirect evidence that pigments are present in light organs come from observations that the light organ of many *Photinus* fireflies undergo a hue change immediately before their activity begins. To human eyes, the light organs of inactive *Photuris* and *Photinus* fireflies appear ivory to pale yellow in color. Just before their twilight display, however, the light organs of *Photinus* species such as *P. australis* and *P. pyralis* acquire a deeper yellow or yellow‐orange hue (Figure 2). After light displays end each evening and *Photinus* fireflies return to rest (or when they are captured), their light organs revert to a paler hue within 10–15 min. Twilight‐active *P. scintillans* undergo a similar hue change from yellow to pink (Faust et al. 2019).

This reversible color change suggests that their light organs contain pigments, which could be newly generated, modified, and/or transported into pigment granules prior to activity. Morphological studies of fireflies have documented “differentiated‐zone granules” within photic cells of the light organ in proximity to mitochondria and peroxisomes (Peterson and Buck 1968; Ghiradella and Schmidt 2004). This description is consistent with pigments, which are synthesized in lysosomal‐related organelles (LROs) via biosynthetic pathways. Pigment biosynthesis occurs through a series of enzymatic reactions that mediate the conversion of pigment precursors into colored substrates. Transporters (e.g., ABC, MFS proteins) move precursors into granules, where they can undergo additional modifications that alter their specific light absorption characteristics (Figon and Casas 2019; Andrade and Carneiro 2021). Both pigment precursors in the cytoplasm and pigments inside granules can absorb light and reflect the remaining wavelengths as color.

To test our hypothesis that pigments in the light organ of *P. pyralis* contribute to the variation in light color between *P. pyralis* populations with identical luciferases, we first determined whether “pigment genes” (genes encoding enzymes and transporters in pigment pathways) are expressed in their light organs (Prediction 1), establishing pigments synthesized in light organs could interact with bioluminescence. Among insects, the most common pigment classes include melanins, carotenoids, pterins, and ommochromes (Andrade and Carneiro 2021). Unlike melanins, which are frequently used for cryptic coloration across animals, and carotenoids, which rely on a dietary uptake, pterins and ommochromes are produced endogenously (Andrade and Carneiro 2021). Due to their universal role in insect pigmentation, particularly as screening pigments in eyes (Langer 1975), our emphasis was on pigments in the pterin and ommochrome pathways. Pterins (red, yellow) and ommochromes (yellow, orange, red, brown, violet) can be used for protection against oxidative damage induced by UV exposure, oxygen radicals, and metabolic toxins, and are integral for visual acuity in insect eyes (Insausti et al. 2013). Since pigments are stored in granules, we also examined whether genes involved in granule formation and/or transport of precursors into granules were expressed. To complement our gene expression analysis, we also extracted pigments from active and inactive light organs to identify which pigments were present in each state (Prediction 2) and whether they could shift light color (Prediction 3).

To determine if pigments could be responsible for the observed light color variation between *P. pyralis* populations, we tested if these pigment, granule, and transporter genes differ in type or expression level (Prediction 4) between the light color extremes of *P. pyralis* (greener light: 560–562 nm versus yellower light: 565–568 nm) in two study populations. Since the expression of pigment biosynthesis and/or transport may change prior to signaling activity (as indicated by the hue change of light organs), we compared pigment gene expression in the light organ between active (twilight) and inactive (morning) fireflies to identify candidate genes underlying the light organ hue change and/or possibly light color. Changes to the intracellular environment where pigments are located can modify their molecular structure, thus influencing light absorption and reflection (Figon et al. 2021). Therefore, cellular processes outside the pigment biosynthesis pathways could contribute to shifting light color. As light color is a continuous trait likely influenced by multiple factors, we used network analysis to identify patterns of gene expression underlying active and inactive light organs, as well as fireflies with variation in light color.

Since screening pigments are known to play an important role in insect eyes (Ziegler 1961; Linzen 1967; Langer 1975), including fireflies (Lall et al. 1988; Cronin et al. 2000), we examined whether any expressed pigment genes and/or transporters in light organs are also expressed in their eyes, thus providing a direct pigment‐based mechanism for the reported tuning between emitted light color (light organ) and the visual sensitivity of firefly eyes (Lall et al. 1980; Seliger et al. 1982a, 1982b; Lall et al. 1988). In addition, we examined whether the bright pink aposematic coloration of the firefly headshield (with unknown pigments) shares any pigments (and biosynthesis pathways) with firefly light organs and/or eyes.

## 2 Methods

### 2.1 Field Collections and Firefly Light Spectra

We collected *P. pyralis* fireflies with light color variation from two natural populations in an urban area of Athens, GA (−83.369901, 33.96541; greener light color, Flint Street) and a farm in Watkinsville, GA (−83.4009, 33.8214; yellower light color, Rose Creek) located about 20 km (straight line distance) from each other (Figure S1). After capture with a net or by hand, we immediately recorded the light spectra of firefly flashes using a portable Jaz spectrophotometer (Ocean Optics Jaz, Orlando, FL) with a laptop computer (SpectraSuite 2.0). Air temperature records were obtained from the Iowa Environmental Mesonet (Herzmann et al. 2004; Tables S1, S2, and S24, Figure S17). Fireflies were either immediately flash‐frozen on dry ice (for gene expression and pigment analysis of active fireflies) or kept overnight at room temperature in Falcon tubes (with small pieces of apple and damp coffee filters to prevent dehydration) before flash‐freezing them 12 h after their activity period (for gene expression and pigment analysis of inactive fireflies). All specimens were stored in a −80°C freezer until pigment extraction or transitioning into RNAlater‐ICE following manufacturer protocol (Thermofisher, Waltham, MA) with storage at −30°C until RNA isolation.

Firefly light spectra collected in the field were analyzed using a custom Mathematica script by fitting a cubic polynomial to raw measurements (Hall et al. 2016). Measurements with a saturated amplitude (light intensity) were removed, as their peak emission wavelengths could not be determined accurately. For each firefly, we calculated the mean wavelength (at peak light intensity) across its individual recorded light spectra, as well as the standard deviation and range (Table S1)(Hall et al. 2016). For transcriptome analysis, we chose a total of 16 *P. pyralis* fireflies (biological replicates) from each population with the most extreme light colors: eight with the most yellow (566–569 nm) flashes from our yellower population and eight with the most green (560–562 nm) flashes from our greener population. Four of the eight fireflies in each color sample were preserved in their active state and four in their inactive state. For the biochemical analysis of pigment substrates, we pooled two replicates from each state (Table S2). To rule out evolutionary divergence between the two populations as a factor in our study, we amplified and sequenced a mitochondrial COI gene region (~620 bp) for all individuals in our study (Appendix 1.1: Appendix S9) and estimated Hudson's Snn statistic for the differentiation of subpopulations (Hudson 2000). We also verified that the luciferases of the fireflies in our study had the same AA sequence (Appendix 1.3: Appendix S9).

### 2.2 Gene Expression Analysis and Experimental Design

For each preserved specimen, we dissected the eyes (light sensing), light organs (LOs; light generation), and thorax muscle (reference tissue not associated with light signaling) from flash‐frozen tissues transitioned into RNA*later*‐ICE. LOs included the clear ventral cuticle, the layer of photic cells where bioluminescence is produced, and the reflective layer. To generate a reference transcriptome for a tissue with conspicuous pink body pigmentation, we extracted RNA from the head shield (HS; pronotum). As this tissue was sparse and fatty, pooling *N* = 3 active individuals achieved an RNA concentration high enough for sequencing. RNA was isolated from all tissues using the Trizol protocol (https://github.com/mspopecki/Gene‐expression‐Photinus‐pyralis.git). All samples were subjected to DNAse treatment (TURBO Invitrogen DNAse) and potential residual organic contamination was removed (Zymo Clean & Concentrator‐25) before quality check (Qubit and Bioanalyzer) and transcriptome sequencing (150‐PE, non‐strand specific) at Novogene (Sacramento, CA). The HS sample was sequenced on a separate run (Table S3).

Transcripts were assessed for quality using FASTQC v0.11.9 (Andrews 2010) with minor trimming using default parameters of TrimGalore! v0.6.5 (Krueger 2015). Trimmed reads were aligned to the *P. pyralis* genome v1.4 (Fallon et al. 2018) with STAR v2.7.3a (Dobin et al. 2013), and transcript abundance was calculated with RSEM v1.3.3 (Li and Dewey 2011). To ensure libraries were high quality and that missing data would not reduce our power to evaluate changes in gene expression, we removed any transcriptomes with fewer than 10% of the average library size from analyses. This resulted in the removal of one transcriptome (FS34I3: inactive light organ, greener light color; Table S3).

**FIGURE 3:**
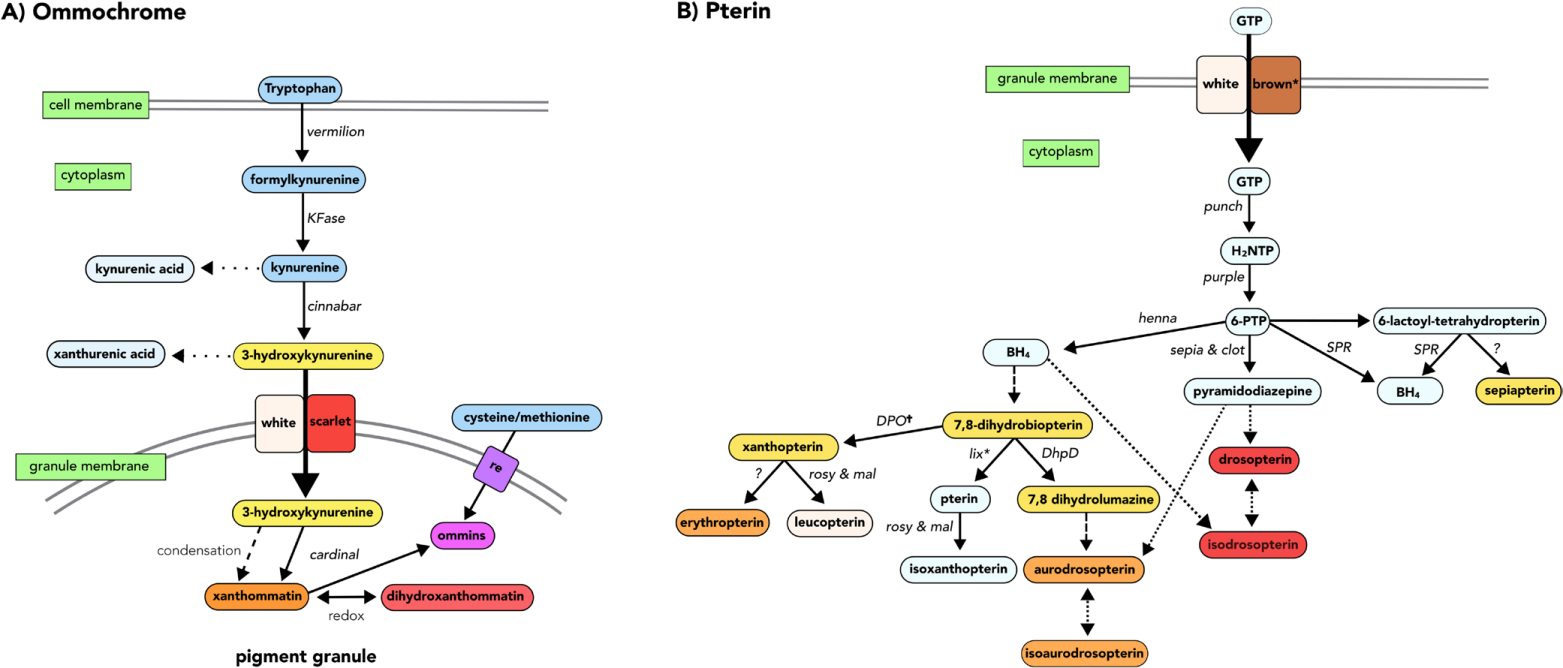
Pigment biosynthesis pathways for (A) ommochromes (modified from Figon and Casas 2018; Vargas‐Lowman et al. 2019; León‐Letelier et al. 2023) and (B) pterins (modified from Vargas‐Lowman et al. 2019; Andrade and Carneiro 2021; Ferré 2024) in *Drosophila melanogaster* . Includes enzymatic (straight lines) and nonenzymatic/condensation (dashed lines) reactions. Boxes: Pigment substrates (with pigment color); gene name of the enzyme catalyzing the respective step (arrow) in *D. melanogaster* . *White, scarlet*, and *brown*: ABC transporters involved in ommochrome and pterin synthesis; *red egg (re)*: MFS transporter in ommochrome pathway; (*) genes we did not identify (expressed < 3 TPM) in *P. pyralis* (Table S5). Ommochrome pathway also includes reactions catalyzed by kynurenine aminotransferase (KAT) (dotted lines), which branch from ommochrome precurors and are involved in detoxification.

We used OrthoFinder v2.3.8 (Emms and Kelly 2019) with the protein genomes of 13 insect species (Table S4) to obtain all gene families (orthogroups) and identify homologous sequences, including putatively duplicated genes. To identify orthologous pigment genes between model organism *Drosophila melanogaster* and *P. pyralis* , we searched orthogroups for the *D. melanogaster* FlyBase identifier (Table S5). We focused on genes involved in ommochrome and pterin biosynthesis (“pigment genes”) (Figure 3) given their widespread use as screening pigments. To identify the MFS pigment transporter gene for *re* in *P. pyralis*, we used the published AA sequence for *Tribolium castaneum* as a query against the *P. pyralis* genome (Fallon et al. 2018) in BLASTP v2.9.0 (NCBI). We chose the best hit to *P. pyralis* (lowest *e*‐value and highest bit score) to identify the most likely ortholog of this gene in *P. pyralis* .

#### 2.2.1 Pigment Gene Expression in Light Organs, Eyes, and Thorax

We identified differentially expressed genes between tissues and/or activity states using the R package DESeq2 v1.36.0 (Love et al. 2014), which uses negative binomial generalized linear models to test for differential expression. We filtered genes with low or no expression by requiring at least three samples to have an expression level of ≥ 3 transcripts per million (TPM) for that gene. We report all pigment genes expressed > 3 TPM in three firefly tissues: LOs, eyes and thorax. In addition, we report pigment gene expression for the HS (aposematic signal), including those in the 90th percentile (top 10%) of genes with the highest expression in the HS.

#### 2.2.2 Differential Gene Expression

Significance values for differential gene expression were adjusted with the Benjamini – Hochberg correction for false discovery rate (FDR) in multiple comparisons (Benjamini and Hochberg 1995). To investigate patterns of differential expression across all transcripts, for contrasts with thorax (Contrast 2) we focused on transcripts with both an adjusted *p*‐value < 0.05 and log_2_fold change greater than |2| but relaxed the log_2_ fold change threshold to |1| when comparing light color (Contrast 1) and activity state (Contrast 3) to investigate subtler changes within tissues. Given the threshold for biological relevance of pigment gene expression is unknown in fireflies, we report all pigment genes that were differentially expressed at *p*‐adjusted < 0.05, both with and without the log_2_fold change threshold for each contrast.

#### 2.2.3 Experimental Design

To test our hypothesis that pigments influence firefly light color, we performed multiple differential expression contrasts across samples and tissues (Figure 2). First, we contrasted the light color (categorized as yellow or green) of individual fireflies by activity state (active, inactive) within the two photic tissues (LOs, eyes) (Contrast 1). Second, we contrasted each active photic tissue against the non‐photic thorax (pooled from all samples), as genes shared between LOs and eyes that are distinct from a non‐photic tissue may have an important role in light signaling (Contrast 2). Finally, we reevaluated differences in activity state using all LO and eye samples, with a focus on differentially expressed pigment genes (Contrast 3).

#### 2.2.4 Pigments in Aposematic Signal: Highly Expressed Pigment Genes in the Head Shield

It is unknown which pigments are used to produce the pink aposematic coloration on the HS of fireflies and whether these same pigments could be utilized in the LO. Thus, we included the HS in our analyses, and calculated the top 90th percentile of expressed transcripts to identify the most highly expressed (HE) genes for comparison with active LOs and eyes.

### 2.3 Weighted Co‐Expression Gene Network Analysis (WCGNA) for Light Organs

We were particularly interested in understanding if co‐expressed gene networks that included pigment genes were associated with signaling activity and light color. Network analysis can also be used for characterizing the metabolic responses underlying the traits of non‐model organisms (Ovens et al. 2021). To identify genes with shared expression patterns across LOs, we complemented our differential expression analyses with a weighted co‐expression gene network analysis using 15 LO samples (4 active yellow, 4 active green, 4 inactive yellow, 3 inactive green; Figure S2) and the R package WCGNA v1.72–1 (Langfelder and Horvath 2008). We prepared transcript count data with the “getVarianceStabilizedData” function from DESeq2, a homoscedastic transformation used to normalize variance across samples for clustering, and generated a signed network, which considers only positive correlations between genes (all genes share the same direction of expression, such as up‐ or down‐regulation). We used hierarchical clustering to group genes with similar expression patterns (low dissimilarity) into sets of co‐expressed genes (modules).

To summarize gene expression within modules, we performed a principal component analysis on module gene expression using the “moduleEigengenes” function. The first principal component, (module eigenvector, or ME) describes the expression pattern for all genes in a module. To determine if module gene expression (ME) was significantly correlated with the mean recorded light color of firefly LOs, we performed a Pearson's correlation using the “cor” function in base R v4.2.1 between module expression (ME = PC1 or summary value of module genes) and light color (nm). We modelled the correlation between ME and activity state (active, inactive) as a discrete variable by fitting a linear model with the “lmFit” function (limma package v3.52.4) in R (Ritchie et al. 2015). To address the variability in expression across samples (from our smaller sample size), we used the limma “eBayes” function to stabilize variance (shift genes with both high and low variation towards the mean variance). The statistics were generated by the limma “topTable” function, which was also used to apply the Benjamini–Hochberg (BH) FDR method correction to *p*‐values to account for multiple comparisons. We considered correlations between ME and either light color or activity state with a *p*‐value < 0.05 and an adjusted *p*‐value < 0.05 as robust; modules with a *p*‐value < 0.05 and an adjusted *p*‐value slightly > 0.05 were reported as “of interest.” Given the limitations of sample size and high variation within natural populations, we included all modules with *p* < 0.05 in our analysis.

#### 2.3.1 Gene Ontology (GO) Enrichment and Module Characterization

To provide biological context for significant gene expression modules, we conducted GO enrichment analyses. For increased annotation accuracy, we did not rely solely on homology with *D. melanogaster* and retrieved *P. pyralis* GO terms using eggNOG‐mapper v2.1.9 (Cantalapiedra et al. 2021), which compares protein orthology across several databases. We subsequently tested for GO term enrichment using the R package TopGo v2.48.0 (Alexa and Rahnenführer 2009) using the default algorithm (weight‐01) for Fisher's Exact Test using all genes expressed in LO as background. Resulting *p*‐values were corrected for multiple testing (BH) with the “*p*.adjust” function from the base R stats function (4.2.1). GO terms with *p* < 0.005 were considered significant. We searched all modules for pigment genes. As gene annotations related to module function and firefly signal production are sparse, we further characterized our modules by comparing them with the gene list published by Fallon et al. (2018), referred to as “LO genes.” Modules were tested for enrichment with pigment and LO genes, in addition to select differentially expressed gene lists using Fisher Exact Test in R; *p*‐values were adjusted for multiple tests with FDR.

#### 2.3.2 Gene Connectivity Within Modules

To understand the interaction of genes within significant modules, we identified highly connected genes (hubs). Hubs are strongly associated with the expression of other genes in the same module and could potentially regulate their gene expression (Yu et al. 2017). We first calculated Module Membership (MM: correlation between expression of a gene and a module eigenvector) using the “signedKME” function. Next, we determined gene significance (GS) as the correlation between the expression of an individual gene and light color using Pearson's correlations. Following Langfelder and Horvath (2008), genes with MM > 0.8 and an absolute value of GS > 0.2 with *p*‐adjusted (BH) < 0.05 were identified as hubs. To test the relationship between gene expression and activity state, we determined GS using a linear model (limma) and established hubs using the same thresholds.

### 2.4 Pigment Extraction

In parallel to our approach for RNA extraction, we dissected eyes, LOs, and HS tissues from 10 additional *P. pyralis* specimens (six active, four inactive) to analyze their pigment substrates (Predictions 2 and 3). Due to the limited number of flash‐frozen fireflies at the green and yellow ends of the light spectrum, we pooled the pigment extracts from two fireflies (each) with similar light emission spectra, for a total of 3 samples for both active LOs and active eyes: (1) emitting green (*x̄* = 561.46 ± 0.067 nm), intermediate yellow‐green (*x̄* = 563.08 ± 0.311 nm), and yellow (*x̄* = 565.30 ± 0.306 nm) light color. In addition, we prepared two inactive samples (*N* = 2) of fireflies emitting yellow light color (*x̄* = 565.080 ± 0.857 nm) and those without light color measurements. For comparison of the relative abundance of pterin substrates in LO and eyes with HS, we also pooled the HS from four active *P. pyralis* specimens to ensure sufficient pigment concentration for analysis (Appendix 2.3: Appendix S9; Table S2).

Our pigment extracts were optimized to extract pterins from firefly tissues. Following Rutowski et al. (2005) for extraction of pterin pigments, we added 400 μL of 1% NH_4_OH (in water) to each tissue sample before grinding the tissues with plastic pestles and an electric hand‐held homogenizer until fully disrupted. Extracts were incubated in the dark on a shaker at low speed at 4°C for 48 h. Extracts were then centrifuged with a Nanosep 0.2 μm BioInert membrane spin column (Pall Corporation) at 5000 rcf for 15 min.

#### 2.4.1 Measuring Absorbance of Pigment Extracts With UV–Vis

To determine if pigments in the light organ could potentially shift the light spectrum of the bioluminescent reaction, we measured the absorbance spectra of our total extracts across the UV and visible spectrum (UV–VIS 325–1000 nm) with a UV–vis (OlisWorks HP 8453), using the extract medium as a blank. We analyzed the spectra with OlisWorks (v1.8.17063.294) and compared the peak absorbance of our samples with the published absorbance spectra of pterins (Figon and Casas 2021; Roca‐Sanjuán et al. 2014; Chen et al. 2007). After UV–vis, the remaining pigment extracts were frozen at −30°C until liquid chromatography with mass spectrometry (LC–MS).

#### 2.4.2 LC–MS

The pterin‐optimized pigment extracts were analyzed on a Thermo Vanquish (ThermoFisher Scientific) connected to a Thermo QExactive HF orbitrap mass spectrometer (ThermoFisher Scientific). A separation was carried out on a Supel Carbon LC (Supelco; 2.1 × 100 mm; 2.7 μm) with a separation condition as follows at 0.3 mL/min: From 0 to 3 min the buffer was held at 90% acetonitrile in 0.1% formic acid, dropped to 50% acetonitrile and held for 8 min, and increased from 50% acetonitrile to 90% acetonitrile for another 12 min to equilibrate the column for the next run. The first 10 min of the separation was fed into the mass spectrometer for analysis while the equilibration was diverted to waste. A data‐dependent program was used for acquisition where the precursor ion scan was acquired at 120 k resolution, followed by top‐down fragmentation of high‐to‐low intensity m/z values by stepped HCD at 30 k resolution. To test for the presence of pigments in the LOs, eyes, and HS, we used the MH+ values and the respective retention times (RT) of known compounds in the guanine‐derived pterin pathway. Despite being optimized for pterins, our pigment extracts also contained ommochrome pigments (Appendix 2.3: Appendix S9). We searched for a total of 17 pterin substrates in *P. pyralis* fireflies with LC–MS (Table S21) identifying any colored pigments as candidates for shifting light color to contextualize our gene expression analysis and inform more in‐depth pigment studies.

## 3 Results

### 3.1 Genetic Variation and Light Spectra of *P. pyralis* Populations

**FIGURE 4:**
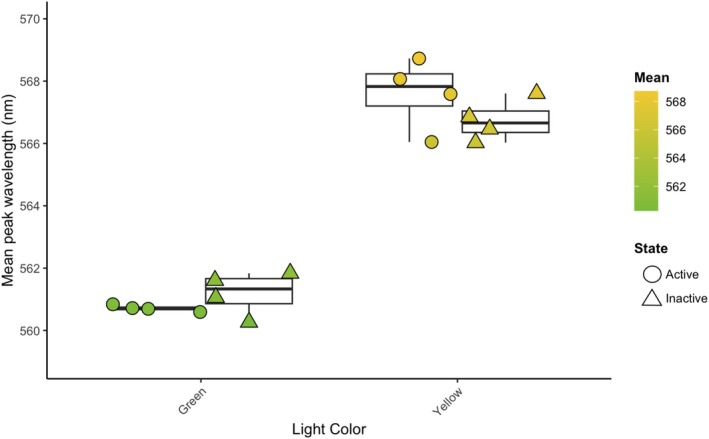
Mean light color (wavelength at peak intensity in nm: *x̄* ± SE) of fireflies from green and yellow populations chosen for transcriptome analysis. Mean: Wavelength (nm) as it corresponds to light color perceived by the human eye. State: Fireflies used for gene expression analysis and preserved in active (circle) or inactive (triangle) state.

We collected *P. pyralis* from two populations in geographic proximity with a difference in mean light color. To rule out evolutionary divergence as a factor, we also sequenced a mitochondrial COI gene region (~620 bp; Folmer et al. 1994; Figure S3) for all individuals and estimated Hudson's Snn statistic for differentiation of subpopulations (Hudson 2000). Mitochondrial (Snn = 0.5; *p* ~ 1) and additional whole‐genome resequencing data confirmed no measurable evolutionary divergence of these populations (manuscript forthcoming). The uniformity of luciferase amino acid sequences between individuals was confirmed (Figure S4). We calculated the mean light color for each firefly as mean wavelength (at highest light intensity) across all their recorded light spectra (Table S1, Figure 4), which were used to determine population means (grouped by activity).

### 3.2 Gene Expression Analysis

Sequencing of multiple tissues from 16 fireflies for transcriptomic analysis (4 active and 4 inactive for each light color category) resulted in a total of 38 transcriptomes across light colors, activity states, and tissues (Table S3) with a mean library size (*x̄* ± SD) of 10,128,638 ± 1,665,770 read counts. Small library size due to missing data can bias results, so we removed samples with less than 10% of the mean library size, resulting in the exclusion of one inactive light organ replicate (green: FS34I3 with 717,767 reads). All sequences are archived at the NCBI Short Read Archive (PRJNA1047132). Eliminating genes with low expression (< 3 TPM) in at least three samples per tissue group resulted in 12,590 (from 15,764) transcripts in our final analyses.

#### 3.2.1 Pigment, Granule, and Transporter Genes in the Genome of *P. pyralis*

**FIGURE 5:**
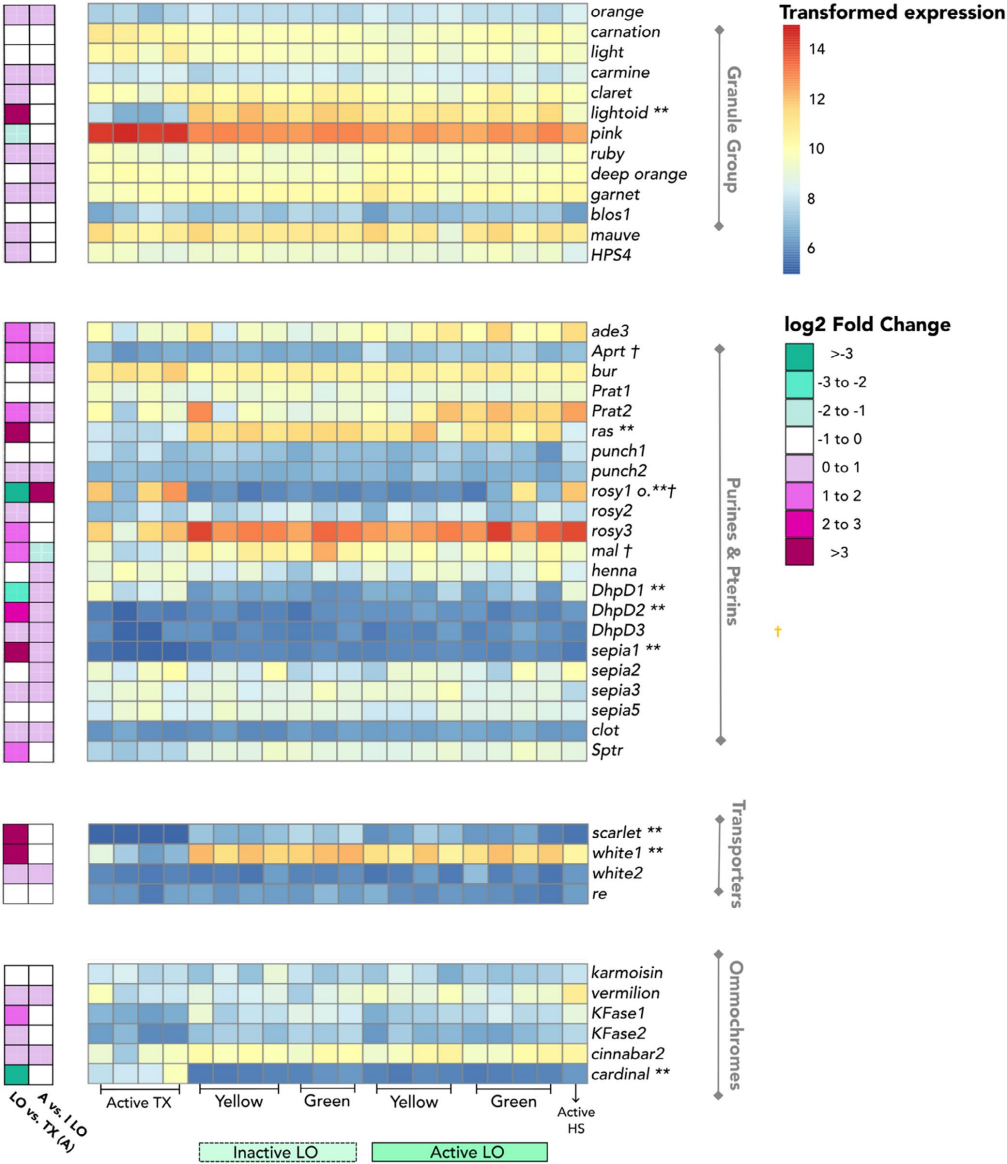
Heatmap (pigment gene expression levels) across active thorax (TX), inactive and active light organs (LO), and active head shield (HS) samples. Ommochromes and pterins (including purines) are ordered according to their approximate placement in the *D. melanogaster* bioisynthesis pathways. The granule group and transporters are unordered. Color legend is scaled by transformed TPM (variance stablizing transformation from DESeq2), and color indicates genes with low (blue), moderate (yellow), or high (red) expression. Pigment genes that were recovered but not expressed > 3 TPM in at least one tissue were omitted. Log_2_fold change values are included to visualize magnitude of expression changes in pigment genes (positive = upregulated in LOs/downregulated in TX, negative = downregulated in LOs/upregulated in TX). Differential expression significance denoted by ** > |2| log_2_fold change (LO vs. thorax) and † > |1| log_2_fold change (active vs. inactive LO).

To identify firefly orthologs of pigment‐related genes in *D. melanogaster* (and *T. castaneum* ), we searched orthogroups (gene families) for 37 different pigment genes, including pterins (with their purine precursors), ommochromes, transporter, and granule genes (Table S5). Overall, 53 pigment genes from 34 orthogroups (including multiple copies) were identified in *P. pyralis* (Table 1, Figure 5; Table S6), though six pigment genes (*purple1*, *purple4*, *white3*, *white4*, *cinnabar1*, and *sepia4*) were not expressed (> 3 TPM) in any firefly tissue (active and inactive replicates pooled). *Purple3* (pterin) was expressed only in HS, and five pigment genes were absent from the thorax (pterins: *DhpD2, DhpD3, sepia1* and ABC transporter: *scarlet*). Seven orthogroups contained increased copy numbers (Figures S5 and S6), with the highest numbers found within pterins. We did not recover any orthologs for *lix* (pterin) or *brown* (ABC transporter in pterin pathway).

**TABLE 1:** Pigment genes used to identify orthologs from the *P. pyralis* genome (PPYR ID; based on list from Croucher et al. 2013).

Gene Name	Class	PPYR ID	Orthogroup	FlyBase ID
*scarlet*	ABC transporter	PPYR_04279	OG0000728	FBgn0003515
*white1*	ABC transporter	PPYR_04280	OG0000728	FBgn0003996
*white2*	ABC transporter	PPYR_04281	OG0000728	FBgn0003996
*white3* ^a^	ABC transporter	PPYR_04282	OG0000728	FBgn0003996
*white4* ^a^	ABC transporter	PPYR_14447	OG0000728	FBgn0003996
*re*	MFS transporter	PPYR_05423	OG0005173	TC013631^b^
*blos1*	Granule	PPYR_11283	OG0008505	FBgn0050077
*carmine*	Granule	PPYR_03448	OG0005216	FBgn0000330
*carnation*	Granule	PPYR_02799	OG0005324	FBgn0000257
*claret*	Granule	PPYR_04283	OG0002347	FBgn0000247
*deep‐orange*	Granule	PPYR_10566	OG0004412	FBgn0000482
*garnet*	Granule	PPYR_11202	OG0004785	FBgn0030089
*HPS4*	Granule	PPYR_14639	OG0006916	FBgn0034261
*light*	Granule	PPYR_02943	OG0005849	FBgn0002566
*lightoid*	Granule	PPYR_05587	OG0006327	FBgn0002567
*mauve*	Granule	PPYR_12819	OG0002750	FBgn0043362
*orange*	Granule	PPYR_02585	OG0006036	FBgn0003008
*pink*	Granule	PPYR_09054	OG0009008	FBgn0029891
*ruby*	Granule	PPYR_09882	OG0003078	FBgn0003210
*cardinal*	Ommochrome	PPYR_05801	OG0003614	FBgn0263986
*cinnabar1* ^a^	Ommochrome	PPYR_12971	OG0002355	FBgn0000337
*cinnabar2*	Ommochrome	PPYR_06324	OG0002355	FBgn0000337
*karmoisin*	Ommochrome	PPYR_05108	OG0007399	FBgn0001296
*Kfase1*	Ommochrome	PPYR_14911	OG0001298	FBgn0031821
*Kfase2*	Ommochrome	PPYR_04835	OG0001298	FBgn0031821
*vermilion*	Ommochrome	PPYR_07427	OG0002319	FBgn0003965
*clot*	Pterin	PPYR_11797	OG0006442	FBgn0000318
*DhpD1*	Pterin	PPYR_00483	OG0001971	FBgn0261436
*DhpD2*	Pterin	PPYR_01709	OG0001971	FBgn0261436
*DhpD3*	Pterin	PPYR_01710	OG0001971	FBgn0261436
*henna*	Pterin	PPYR_10049	OG0004429	FBgn0001208
*mal*	Pterin	PPYR_07656	OG0004893	FBgn0002641
*punch1*	Pterin	PPYR_14140	OG0003359	FBgn0003162
*punch2*	Pterin	PPYR_14143	OG0003359	FBgn0003162
*purple1* ^a^	Pterin	PPYR_04028	OG0002731	FBgn0003141
*purple2*	Pterin	PPYR_09660	OG0002731	FBgn0003141
*purple3*	Pterin	PPYR_09659	OG0002731	FBgn0003141
*purple4* ^a^	Pterin	PPYR_15506	OG0002731	FBgn0003141
*rosy1*	Pterin	PPYR_11236	OG0000709	FBgn0003308
*rosy2*	Pterin	PPYR_11237	OG0000709	FBgn0003308
*rosy3*	Pterin	PPYR_11235	OG0000709	FBgn0003308
*sepia1*	Pterin	PPYR_09852	OG0000366	FBgn0086348
*sepia2*	Pterin	PPYR_13566	OG0000366	FBgn0086348
*sepia3*	Pterin	PPYR_02423	OG0000366	FBgn0086348
*sepia4* ^a^	Pterin	PPYR_14507	OG0000366	FBgn0086348
*sepia5*	Pterin	PPYR_14121	OG0000366	FBgn0086348
*Sptr*	Pterin	PPYR_12709	OG0008551	FBgn0014032
*ade3*	Purine	PPYR_13352	OG0002091	FBgn0000053
*Aprt*	Purine	PPYR_00822	OG0006160	FBgn0000109
*bur*	Purine	PPYR_12914	OG0005692	FBgn0000239
*Prat1*	Purine	PPYR_03533	OG0001300	FBgn0004901
*Prat2*	Purine	PPYR_10414	OG0001300	FBgn0004901
*ras*	Purine	PPYR_05313	OG0003143	FBgn0003204

*Note:* FlyBase ID = Identity of *D. melanogaster* genes and protein ID of *T. castaneum* *red egg (re)*: Unique to beetles.

^a^ Was not expressed in any tissue (LO, eyes, thorax) > 3 TPM.

^b^ Gene derived from *Tribolium castanaum* (Osanai‐Futahashi et al. 2012).

### 3.3 Pigment Gene Expression

Of the 53 pigment genes in the pterin and ommochrome pathways identified in the *P. pyralis* genome, 46 were expressed (> 3 TPM) in *P. pyralis* LOs (Table S6), where they could potentially contribute to the formation of colored pigment products (Prediction 1). In *P. pyralis* eyes, the same 46 pigment genes were expressed (> 3 TPM; Table S6) and could have a role in tuning visual sensitivity. *Cinnabar1* was expressed just above our threshold at extremely low levels in eyes (*N* = 16, mean TPM = 3.50 ± 6.32 SD, Table S6), so we did not include it in expressed gene counts. To identify the most likely candidates for shifting light color in LO and/or for tuning between light spectra and visual sensitivity, we used three contrasts to identify pigment genes that were differentially expressed between light colors, tissue type, and activity. We identified the pigment genes with the most substantial differential expression (log_2_fold change), but since any pigment present may impact light color and tuning, we also reported log_2_fold changes below these thresholds.

#### 3.3.1 Contrast 1: Light Color

To test our hypothesis that pigments in the LO contribute to the emitted light color in fireflies due to the differential expression of pigment genes (Prediction 4), we contrasted the active LOs of *P. pyralis* with yellow and green signals. There were 88 differentially expressed genes (21 upregulated in yellow, 67 upregulated in green), while the same contrast with inactive LO yielded 19 differentially expressed genes (14 upregulated in yellow, 5 upregulated in green). Only one pigment gene (*rosy1*) was differentially expressed between active yellow and green LOs (upregulated in green LOs, though this was likely driven by an outlier; Table S7, Figure S8).

To identify parallel changes in eyes, we compared pigment gene expression in the eyes of fireflies with yellow or green flashes. During activity, 105 genes were differentially expressed (77 upregulated in yellow, 28 upregulated in green) between the eyes of green and yellow fireflies. One pigment gene, *sepia3*, was upregulated in the eyes of active fireflies with yellow light (log_2_fold change = 1.022). In inactive eyes, only five genes were differentially expressed (all 5 upregulated in yellow versus green); none of these were pigment genes.

#### 3.3.2 Contrast 2: Tissues (Active State)

Given that tissue type was the primary driver of gene expression differences across samples (Figure S7), and our expectation that the pigment genes most relevant to light signals should be more highly expressed in photic tissues, we contrasted LOs and eyes with the thorax of active fireflies. We reported all significantly differentially expressed pigment genes (and whether they differed at a log_2_fold change of > 2 or < 2).

##### 3.3.2.1 Active LO Versus Thorax

**FIGURE 6:**
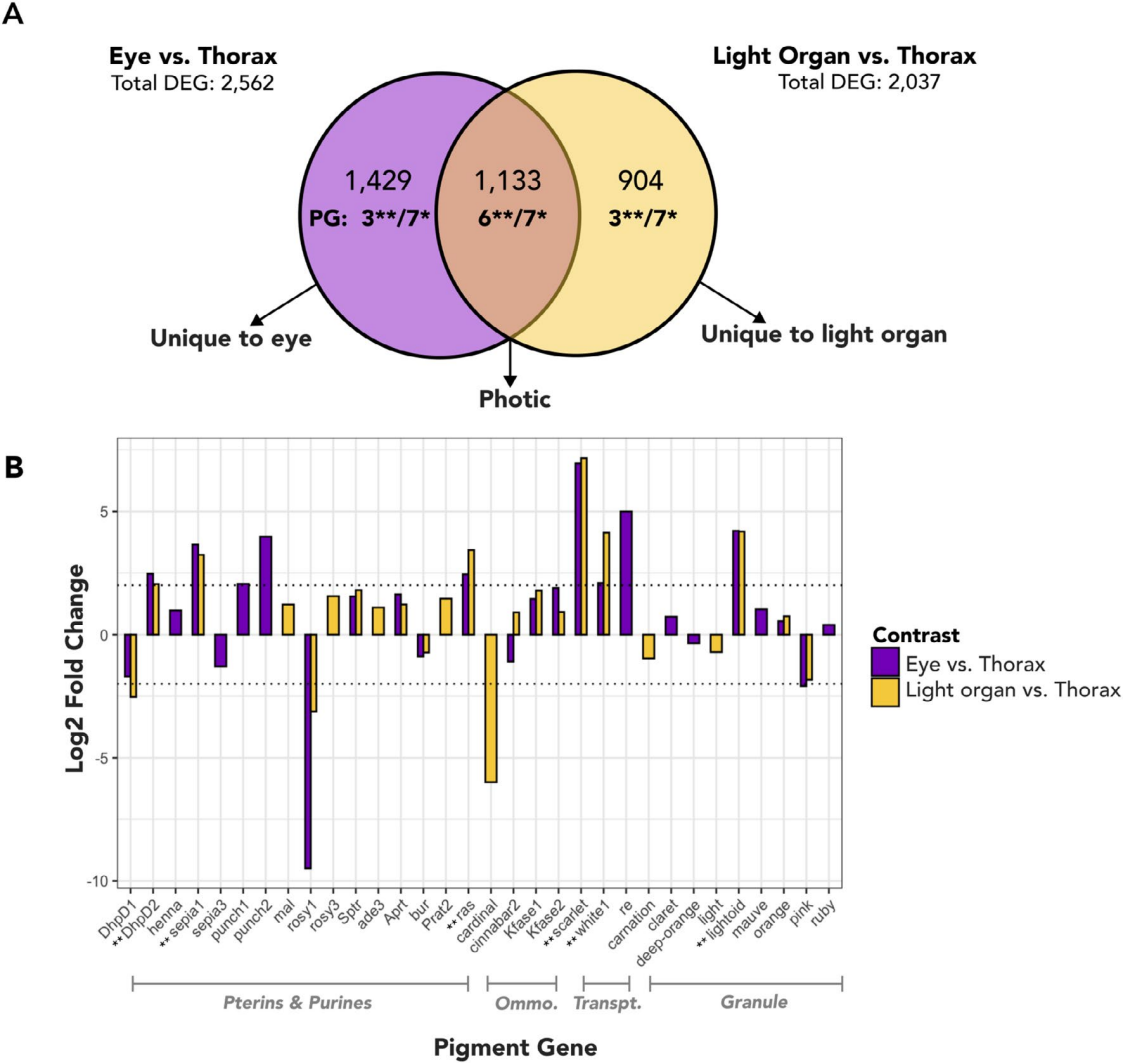
Pigment genes expressed in both light organs (LOs) and eyes that differ from thorax. (A) Results of Contrast 1: Comparison of differential gene expression between tissues (active state). *Significant *p*‐adjusted (BH) DEG; **Significant *p*‐adjusted (BH) and log_2_fold change > |2|. (B) Expression patterns of 32 pigment genes in the light organs and eyes of active fireflies that were differentially expressed from thorax, including 16 photic genes, 9 genes uniquely DE in the eye, and 7 genes uniquely DE in LO. Apart from *cinnabar2*, all other pigment genes DE with thorax in both LO and eyes show the same directionality of expression. Dotted lines on the y‐axis (at 2 and −2 log_2_fold change) indicate the significance thresholds used for Contrast 2: (1) log_2_ fold change > |2| and *p*‐adjusted < 0.05, (2) *p*‐adjusted < 0.05. Pigment gene names on the x‐axis labeled ** indicate significant p‐adjusted (BH) and log_2_fold change > |2|.

There were 2037 differentially expressed genes between active LOs and the thorax (1260 upregulated in the light organ, 777 upregulated in the thorax; Figure 6A). Of the 1260 genes expressed more highly in LOs, six were pigment genes, including 2 pterins (*DhpD2*, *sepia1*), 2 transporters (*scarlet, white1*), and 1 granule gene (*lightoid*; Table S7). Relative to the thorax, three pigment genes were downregulated in LO (2 pterins: *DhpD1, rosy1*, and 1 ommochrome: *cardinal*); however, the expression pattern of *rosy1* was driven by one sample (outlier; Figure S8). We identified 10 additional pigment genes that were significantly upregulated (log_2_ fold change < |2|) in active light organs (and four downregulated; Table S7) that could also affect light color, including three pterins (*mal*, *rosy3*, and *Sptr*) and three ommochromes (*cinnabar2, KFase1*, and *KFase2*). Significant KEGG pathways are reported in Table S8, Figure S15.

##### 3.3.2.2 Active Eye Versus Thorax

Between active eyes and thorax, there were 2562 differentially expressed genes (in eyes: 1503 upregulated, 1059 downregulated). Of these, nine pigment genes were upregulated in eyes (4 pterins: *DhpD2, sepia1, punch1, punch2*, 2 ABC transporters: *scarlet, white1*, 1 MFS transporter: *re*, and 2 granule genes: *lightoid, ras*), whereas two were downregulated in active eyes: 1 pterin (*rosy1*) and 1 granule gene (*pink*). There were 14 pigment genes (9 upregulated, 5 downregulated) that were significantly DE in eyes (log_2_fold change < |2|; Table S7), including those downregulated in eyes: *DhpD1* and *sepia3* (pterins), as well as *cinnabar2* (ommochrome). For significant KEGG results, see the Table S9, Figure S16.

##### 3.3.2.3 Shared Differential Expression for Both Active LO and Eye Versus Thorax

We identified 1075 genes that were differentially expressed from thorax in both LOs and eyes (600 upregulated, 475 downregulated in photic tissues), including 12 pigment genes (Figure 6A).

Seven pigment genes were DE with > |2| log_2_fold change (Figure 6); of these, six were more highly expressed in both LOs and eyes (i.e., DE with, or absent, from thorax): *DhpD2, sepia1, ras, scarlet, white1*, and *lightoid*. Only one DE pigment gene, *cinnabar2* (ommochrome), displayed a divergent expression pattern between the two photic tissues (upregulated in LOs but downregulated in eyes, relative to thorax). The remaining five other pigment genes were significantly DE with log_2_fold change < |2| relative to thorax: *KFase1, KFase2, Sptr, Aprt*, and *orange*. A total of four pigment genes were downregulated in both photic tissues relative to thorax at both thresholds: *DhpD1, rosy1, bur, pink* (Figure 6A, Table S7). To gain functional context for all genes upregulated in both LO and eyes (photic genes), we performed GO enrichment (Table S11) and compared with LO genes from Fallon et al. (2018), yielding an overlap of 9 photic genes (Table S10).

##### 3.3.2.4 Tissue‐Specific Expression

To understand which genes were important in a specific photic tissue, we identified genes that were upregulated or downregulated (relative to thorax) only in LOs (i.e., upregulated in LO vs. TX, but not eye vs. TX). Similarly, we identified genes upregulated only in eyes. Of the 1260 genes upregulated in LO, 660 genes were upregulated only in LOs; including 34 LO genes from Fallon et al. (2018) (Table S10), such as luciferase (PPYR_00001) and luciferin sulfotransferase (PPYR_00003), whose products are key for bioluminescence. No pigment genes were upregulated only in LOs at a log_2_fold change > 2, however, four pigment genes were upregulated at log_2_fold change < |2|, including two pterins (*mal, rosy3*). Of 292 genes downregulated only in LOs relative to thorax, three were pigment genes: *cardinal* (ommochrome) (log_2_fold change > |2|), as well as *carnation* and *light* (log_2_fold change <|2|). Conversely, between eyes and thorax, a total of 903 genes were only upregulated in eyes and 574 genes were only downregulated in eyes, including a total of nine pigment genes (Figure 6; Table S7). GO enrichment of differentially expressed genes in LOs and/or eyes are reported in Tables S11–S13.

#### 3.3.3 Contrast 3: Activity

**FIGURE 7:**
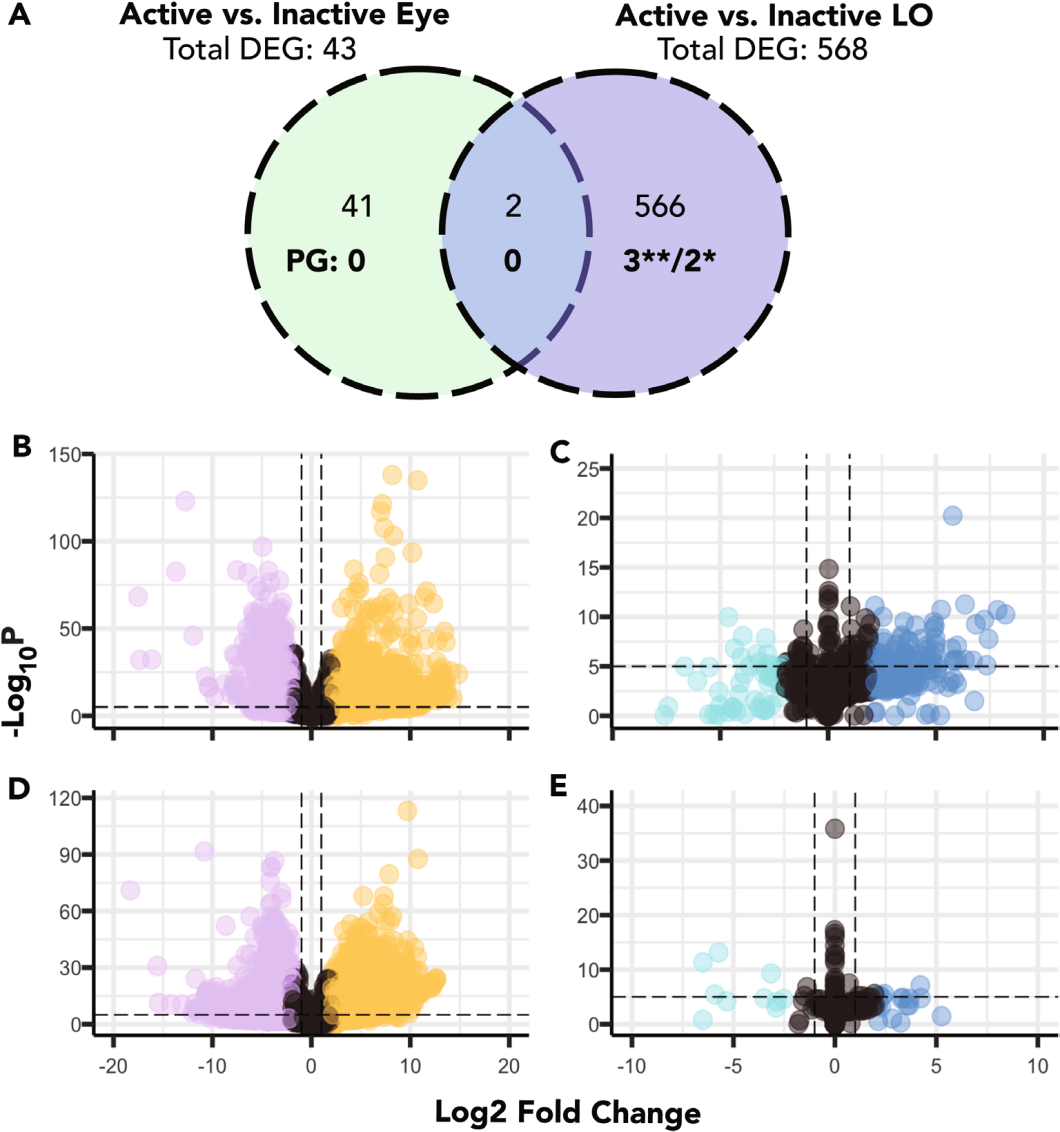
(A) Differential gene expression in light organs (LOs) and eyes between active and inactive fireflies. Of 43 genes differentially expressed between active and inactive eyes, none were pigment genes. Of 568 genes differentially expressed between active and inactive LOs, five were pigment genes. Overall, 46 pigment genes were expressed (> 3 TPM) in both states of LO and in eyes (Table S6). **Significant *p*‐adjusted (BH) and log_2_fold change > |1|, *Significant *p*‐adjusted (BH) DEG. Comparison of gene expression between (B) LO versus TX (active), (C) Active versus inactive LO, (D) Eye versus TX (active), and (E) Active versus inactive eye. Dashed lines indicate thresholds used for *p*‐value (horizontal) and log_2_fold change (vertical) for Contrasts 2 and 3. Plots made with R package EnhancedVolcano v1.20.0 (Blighe et al. 2018).

We investigated transcriptional changes that occur in the photic tissues of active fireflies by comparing LO and eye samples while signaling (active at twilight) and at rest (inactive in the morning). Compared with Contrast 2 (tissue), fewer genes were DE with activity in LO and eyes. Between LO activity states, 568 genes were DE (active: 393 upregulated; 175 downregulated; Figure 7). This included five pigment genes: *Aprt* and *rosy1* were upregulated in active LOs, whereas *mal* was upregulated when fireflies were inactive (log_2_fold change > |1|). Active LO samples had higher expression of *rosy1* than inactive LOs (Figure S8), but the statistical significance of this expression difference appeared to be driven by one (green active) LO sample (Figure S8). Pigment genes DE with log_2_fold change < |1| were *DhpD1* (upregulated in active) and *lightoid* (upregulated while inactive). Only *Cytochrome‐P450* (PPYR_06980) of the LO genes from Fallon et al. (2018) was upregulated in inactive LOs. Between activity states in the eye, we detected 43 differentially expressed genes (in active: 32 upregulated, 11 downregulated). Pigment gene expression remained stable across activity states in the eye, with no differential expression detected (Figure 7). We identified 150 genes that were upregulated in active LO (using both thorax and activity contrasts). Of these 150 genes, none were pigment or LO genes; however, as these genes could be otherwise relevant to signaling in LO, the list is reported in Table S14.

#### 3.3.4 Pigments in Colored Body Tissue (Head Shield)

Overall, 45 pigment genes were expressed > 3 TPM in the HS of *P. pyralis* fireflies (Table S15). Notably, two genes, *scarlet* (ABC transporter into ommochrome granules) and *sepia1* (pterin), which were differentially expressed with thorax in both LOs and eyes, were not expressed in HS. The 90th percentile of highest expression (HE) in HS consisted of 1133 genes, including 10 pigment genes (Table S15). These 10 pigment genes included two pterins (*rosy1, rosy 3*), two ommochromes (*vermilion, cinnabar2*), and one transporter gene (*white1*). Of these, *white1* was also upregulated in both LOs and eyes relative to thorax, and *cinnabar*2 was upregulated in LOs only (but not eyes) when contrasted with thorax (log_2_fold change < | 2 |).

### 3.4 Network Analysis of Light Organ Samples

A total of 12,572 genes were used to build a signed network (blockwiseModules function, R with power = 14) with default parameters, yielding 40 different co‐expression modules ranging in size from 33 to 1829 genes (*x̄* = 314 ± 372 genes; Tables S16 and S17). In 23 of these modules, we identified at least one pigment gene. There were 62 hubs significantly (adjusted *p*‐value < 0.05) associated with activity state across 10 modules; none were pigment genes (Table S18). No hubs were associated with light color, though two modules had marginally significant (*p* < 0.05, *p*‐adjusted > 0.05) associations with light color. Notably, in both modules, there is a larger variation within the active yellow LO samples compared to inactive yellow LOs or active and inactive green LO samples, suggesting that the difference between yellow and green LOs emerges when yellow LOs transition from the inactive to active state (Figure S9).

#### 3.4.1 Modules Associated With Light Organ Activity State

**FIGURE 8:**
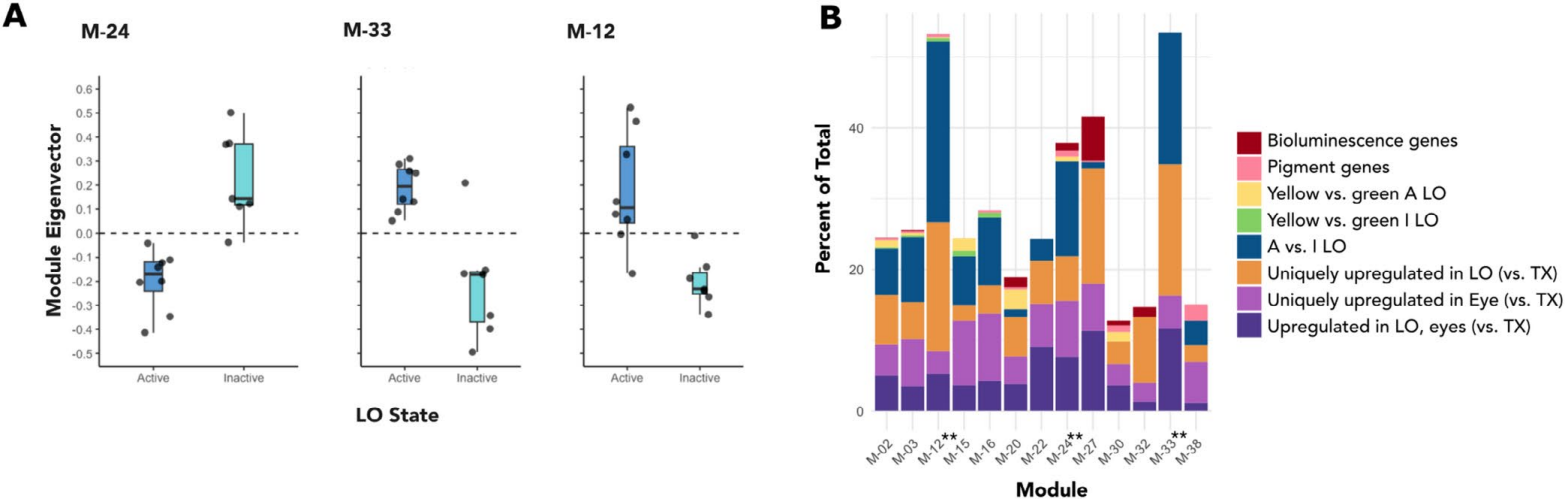
(A) Gene network modules with expression patterns significantly associated with light organ (LO) activity. The 659 genes in M‐12 (“green”) and the 43 genes in M‐33 (“skyblue3”) were more highly expressed in active light organs; the 539 genes in M‐24 (“pink”) were more highly expressed in inactive LOs. (B) Proportion of pigment and bioluminescence (LO, Fallon et al.  (2018) genes, as well as DEG, within significant modules (** denotes highly significant, *p* < 0.05 and *p*‐adjusted < 0.05). A: active, I: inactive, TX: thorax.

Three modules were significantly associated (*p*‐adjusted < 0.05) with activity state: The genes in module M‐24 (“pink”) had a significantly higher expression in inactive LOs, while the genes in modules M‐12 (“green”) and M‐33 (“skyblue3”) were significantly higher expressed in active LOs (M = 12 had borderline significance, with *p*‐adjusted = 0.0551; Table 2, Figure 8). We identified nine other modules with marginal significance, with five modules upregulated in active LOs and four upregulated in inactive LOs, presented in the Tables S16–S20. For our analysis, we focused primarily on the three modules that had the greatest statistical association with activity state: M‐33, M‐12, and M‐24. Module characterization (e.g., hub genes, enrichment, and GO terms) is reported in Tables S18–S20.

**TABLE 2:** Modules significantly associated with (a) the light organ activity state (A = active, I = inactive) and/or (c) emitted light color (G = green, Y = yellow). Activity: logFC = DE between active vs. inactive LO, *t* = moderated t‐statistic, *B* = B‐statistic (linear model, lmFit from limma). Light color: *r* = correlation between light color (nm) and ME (Pearson's correlation).

Module	Module color	*N*	Expression directionality^a^	logFC^a^	*t^a^*	*B^a^*	*p^a^ *	adj. *p^a^*	Expression directionality^c^	*r^c^*	*p* ^c^	adj. *p^c^*
M‐24	Pink	539	↑I ↓A	−0.4240	−3.2249	−1.1295	0.0013	0.0419	↑G ↓Y	−0.0871	0.7576	0.9268
M‐33	Skyblue3	43	↑A ↓I	0.4065	3.0919	−1.5261	0.0021	0.0419	↑G ↓Y	−0.1574	0.5753	0.8075
M‐12	Green	659	↑A ↓I	0.3787	2.8806	−2.1221	0.0041	0.0551	↑G ↓Y	−0.0193	0.9455	0.8075
M‐02	Blue	1413	↑A ↓I	0.3354	2.5515	−2.9663	0.0110	0.1101	↑Y ↓G	0.3677	0.1775	0.9268
M‐27	Red	546	↑I ↓A	−0.3131	−2.3815	−3.3625	0.0176	0.1304	↑G ↓Y	−0.0348	0.9021	0.8075
M‐16	Lightcyan	282	↑I ↓A	−0.3079	−2.3419	−3.4508	0.0196	0.1304	↑Y ↓G	0.1832	0.5134	0.9184
M‐20	Midnightblue	286	↑I ↓A	−0.2992	−2.2759	−3.5946	0.0233	0.1304	↑G ↓Y	−0.5125	0.0508	0.8075
M‐32	Skyblue	75	↑I ↓A	−0.2875	−2.1870	−3.7821	0.0292	0.1304	↑G ↓Y	−0.2324	0.4045	0.9455
M‐22	Orangered4	33	↑A ↓I	0.2870	2.1834	−3.7896	0.0295	0.1304	↑G ↓Y	−0.2285	0.4128	0.8075
M‐15	Grey60	274	↑I ↓A	−0.2769	−2.1061	−3.9460	0.0357	0.1304	↑G ↓Y	−0.0533	0.8504	0.9450
M‐03	Brown	748	↑A ↓I	0.2764	2.1022	−3.9537	0.0360	0.1304	↑Y ↓G	0.0665	0.8138	0.8144
M‐38	White	86	↑A ↓I	0.2719	2.0681	−4.0209	0.0391	0.1304	↑G ↓Y	−0.1889	0.5001	0.8075
M‐30	Salmon	305	↑I ↓A	−0.0245	−0.1861	−6.0244	0.8524	0.8743	↑G ↓Y	−0.5402	0.0376	0.8144

#### 3.4.2 Modules Associated With Active Light Organs

Two modules (M‐12 “green” and M‐33 “skyblue3”) were significantly associated with active LOs. The “green” (M‐12) module included pigment genes (pterins *DhpD1, sepia1*, and *clot*), in addition to genes associated with circadian rhythm and light perception, such as *arrestin domain‐containing protein 17‐like* (PPYR_02715; Table S17). We identified 13 hubs in M‐12 (Table S18) in addition to genes DE between yellow and green active eyes (2 genes; Table S17), and genes DE between yellow and green LOs (4 genes), including *modular serine protease‐like* (PPYR_13682).

The “skyblue3” (M‐33) module was small with only 43 associated genes, eight of which were hubs (Table S18), including *aristaless‐related homeobox protein‐like | retinal homeobox protein Rx1‐like* (PPYR_00572), a gene involved in vertebrate eye development (Nelson et al. 2009). This module also contained *phenoloxidase‐activating factor 1‐like* (PPYR_14004) and *CLIP domain‐containing serine protease 2‐like| phenoloxidase‐activating factor 3‐like* (PPYR_01625), which activate melanogenesis in the immune response. There were 16 genes in M‐33 (“skyblue3”) that were annotated as “uncharacterized protein” or lacked annotation, suggesting species‐specific function. Both M‐12 (“green”) and “ M‐33 (“skyblue3”) modules were significantly enriched for genes DE with activity (M‐12 FDR = 1.04E‐84, M‐33 FDR = 2.95E‐03) and LO‐specific genes (M‐12 FDR = 2.63E‐35 and M‐33 FDR = 9.06E‐03; Table S19).

#### 3.4.3 Modules Associated With Inactive Light Organs

The “pink” (M‐24) module included transcripts upregulated in inactive LO. Four of these were pigment genes (ommochrome: *cardinal*, ABC transporter: *scarlet*, granule: *lightoid, pink*). *Pink* is part of the biogenesis of lysosome‐related organelles complex (BLOC1‐4), which is involved with the formation of pigment granules via intracellular trafficking (Falcón‐Pérez et al. 2007). Other members of the BLOC family were observed: *ras‐related protein Rab‐32|ras‐related protein Rab‐38* (PPYR_05587), encoded by BLOC‐3 (Gerondopoulos et al. 2012), and *biogenesis of lysosome‐related organelles complex 1 subunit 4* (PPYR_10058).

As with modules “green” (M‐12) and “skyblue3” (M‐33), in the “pink” (M‐24) module we observed genes related to phototransduction, including four copies of *retinol‐binding protein pinta‐like* (Table S17). We also noted genes that play a role in eye development and/or maintenance: *homeobox protein OTX1‐like|homeobox protein OTX2‐like* (PPYR_13979), *rab11 family‐interacting protein 2* (PPYR_05444), *ras‐related protein Rab‐28‐like* (PPYR_12570), and *chaoptin‐like* (PPYR_14596), the latter of which was also a hub (Table S18). Along with genes associated with signal reception were six LO genes in module “pink” (M‐24) (Table S17), including *luciferin‐sulfotransferase* (PPYR_00003), which is integral for the bioluminescent reaction. This module also included two other genes associated with photoreception, both of which were upregulated only in eyes (relative to thorax) and differentially expressed between LO activity states (PPYR_12570, PPYR_12713). Importantly, in module “pink” (M‐24) we identified 19 hubs (Table S18) in addition to genes DE betw yellow and green LOs (*N* = 4) as well as yellow and green eyes (*N* = 8) (Table S17). Module “pink” (M‐24) was significantly enriched for photic genes (FDR = 1.25E‐02) and genes DE with activity (FDR = 1.30E‐16; Table S19).

### 3.5 Pigment Analysis

As a complement to our gene expression analysis, we conducted a pigment analysis of LOs and eyes (Prediction 2). Our UV–vis analysis of pterin‐optimized extracts (Figure S11) showed an absorption peak around 370 nm, which is characteristic of pterins that absorb light < 400 nm (Wijnen et al. 2007; Andrade and Carneiro 2021). Notably, these absorption curves extended into the visible spectrum (Johnson and Fuller 2015), which includes the range of *P. pyralis* light signals (560–568 nm).

For LC–MS, we used pooled samples (*N* = 2 fireflies each) of the LOs and eyes of active fireflies that emitted green light (*x̄*=561.46 ± 0.067 nm), intermediate yellow‐green light (*x̄*=563.08 ± 0.311 nm), and yellow light (*x̄*=565.30 ± 0.306 nm). For comparison between activity states, we also analyzed pooled samples (2 fireflies each) of inactive fireflies that emitted yellow light (*x̄* = 565.080 ± 0.857 nm) or had no recorded light spectra (“NS”; Table S2). Despite being optimized for pterins, our extracts also contained eight substrates in the ommochrome pathway (Appendix 2.3: Appendix S9, Figures S13 and S14). We identified 17 compounds from the pterin pathway in *P. pyralis* fireflies with LC–MS (Table S22). The most abundant pterins in LOs were leucopterin (white) and xanthopterin/isoxanthopterin (yellow/colorless), as well as 7,8‐dihydrolumazine (yellow). These pigments varied across both light colors and activity states.

#### 3.5.1 Light Colors

The yellow 7,8‐dihydrolumazine was present at a higher relative abundance (more than double) in active yellow (6.72%) and yellow‐green (8.97%) LOs compared to green LOs (2.89%; Table S22). In parallel with LOs, 7,8 dihydrolumazine (yellow) was relatively more abundant in active yellow eyes (77.26%) and active yellow‐green (66.77%) eyes, compared to active green eyes (28.48%), providing a possible mechanism of tuning between yellower light emission spectra and yellower visual sensitivity. However, since we know only the relative abundance of pigments (dependent on the abundance of other pigments), we do not know how this translates to the actual pigment level in each sample.

#### 3.5.2 Active Versus Inactive Yellow Light Emitting Fireflies

The LOs of active yellow fireflies contained a relatively lower abundance of xanthopterin/isoxanthopterin (yellow/colorless) than the LOs of inactive yellow fireflies (active: 41.20% vs. inactive: 45.45%), along with a relatively higher amount of both leucopterin (active: 50.36% vs. inactive: 46.84%) and 7,8 dihydrolumazine (active: 6.72% vs. inactive: 4.0%; Table S22). In parallel to their LOs, the eyes of active yellow fireflies contained relatively higher levels of 7,8 dihydrolumazine (77.26%) than the eyes of inactive yellow fireflies (56.97%; Table S22). Leucopterin (active: 3.53% vs. inactive: 9.45%) and xanthopterin/isoxanthopterin (active 4.00% vs. inactive: 10.05%) levels were both lower in active yellow eyes compared to their inactive state. The relative abundance of pterin substrates in the inactive samples without light spectra was consistent with others, supporting the reliable detection of these pterin substrates across tissues and activity states. Overall, a PCA with all 17 pterin compounds suggests the pterin profile of active LOs is similar (Figure S12), and testing for differences between active yellow and green LOs across all pterin substrates showed no statistical difference (Welch's two‐sample *t*‐test, *p* = 0.9998: Appendix 1.5: Appendix S9).

#### 3.5.3 Coloration in the Head Shield

In our pooled HS sample (*N* = 4), the most abundant pigments in the pterin pathway were xanthopterin/isoxanthopterin (49.57%), 7,8‐dihydrolumazine (27.78%), and leucopterin (8.92%; Table S22).

## 4 Discussion

Our results show that ommochrome and pterin pigment genes are indeed expressed in the light organs (LOs) and eyes of *P. pyralis* fireflies, and at surprising numbers. The same 46 pigment genes from the ommochrome and pterin biosynthesis pathways were expressed (> 3 TPM) in both *P. pyralis* LOs and eyes (Table S6). Therefore, the production, transport, and/or storage of the colored pigments generated by these genes could filter the light generated by luciferase in firefly LOs and contribute to the visual tuning between spectral sensitivity and light color (Seliger et al. 1982a, 1982b; Lall et al. 1988). Contrary to our initial expectation, we did not identify any differentially expressed (DE) pigment genes between the LO of *P. pyralis* with “yellow” (565–568 nm) and “green” (560–562 nm) light color, and in the eyes only one pigment gene, *sepia3*, was upregulated in fireflies with yellower light. Thus, detecting more subtle differences in gene expression potentially relevant to the low levels of light color variation between our two *P. pyralis* populations may require larger numbers of replicates to achieve greater statistical power. It is unknown whether fine scale variation of pigment gene expression is biologically relevant to fireflies or whether the intraspecific variation is driven by genetic changes with large effect substantial enough for detection in current statistical tests. If only slight differences in pigment composition are needed to shift light color, then they could lie beneath detection limits of the current study. Given that pterin and ommochrome pathway products are used for detoxification and preventing oxidative damage (Insausti et al. 2013), lack of differential expression could be explained by their important role in maintaining cellular function across tissues. Our tissue and activity contrasts yielded important insights toward addressing these questions.

The transition from a resting to signaling firefly involves diel changes in physiology, including the hue change observed in *P. pyralis* LOs prior to the onset of activity at twilight. Interestingly, a much greater transcriptional change occurred between activity states in LOs (*N* = 568) compared to eyes (*N* = 43), indicating there is a heightened biological response in LOs in preparation for signaling. This response included three pigment genes that were expressed significantly higher in active LOs: *rosy1* (Figure S8), *DhpD1*, and *Aprt*, and two downregulated pigment genes: *mal* and *lightoid*. In contrast, no pigment genes were differentially expressed with activity in eyes. As screening pigments are integral for vision and protect eyes from UV light, the 46 pigment genes in eyes may be consistently expressed with only small changes in expression. Preliminary LC–MS analysis of pigment extracts was consistent with these trends in gene expression. Overall, the different tissue extracts displayed unique pigment profiles (Table S22), and a PCA clustering analysis suggests differences between tissues and activity states (Figure S12).

### 4.1 Shared Patterns of Pigment Gene Expression in LOs and Eyes (vs Thorax)

Active *P. pyralis* fireflies expressed 15 “photic” genes in both LOs and eyes. Interestingly, 14 of these pigment genes shared the same directionality (i.e., upregulated or downregulated relative to thorax), suggesting the same types of pigments are synthesized for use in light emission (LOs) and signal detection (eyes). These pigments could therefore contribute to the tuning between light spectra and visual sensitivity (Lall et al. 1988; Cronin et al. 2000). Among the 14 genes, we identified five pigment genes involved in consecutive steps of the ommochrome pathway: *KFase1, KFase2*, and *cinnabar2*, as well as the ABC transporters *white1* and *scarlet*. This suggests that in both LOs and eyes, kynurenine (light yellow) is produced (*KFase*) and converted (*cinnabar*) into the yellow pigment 3‐hydroxy kynurenine (3‐OHK). The 3‐OHK can either remain in the cytoplasm (Llandres et al. 2013) or be imported into ommochrome granules by the *white* and *scarlet* transporter pair. The expression of the ABC transporters *white* and *scarlet* indicates that 3‐OHK is transported into the ommochrome granules in both photic tissues, where it could get converted nonenzymatically into xanthommatin (yellow‐brown; Figon et al. 2020) and/or other pigments. For example, in *Drosophila*, xanthommatin can be reduced to dihydroxanthommatin (red), or converted into ommins (violet) via either nonenzymatic condensation of xanthommatin with a sulfur‐containing derivative of methionine/cysteine (Figon et al. 2020), or enzymatically by *cardinal* (Shirai and Daimon 2020; Xu et al. 2020).

The low *cardinal* activity in LOs and a lack of nonenzymatic conversion (if present) of 3‐OHK into xanthommatin should correspond with high levels of 3‐OHK (substrate), which produces yellow coloration in crab spiders (Riou and Christidès 2010) and *Heliconius* butterflies (Finkbeiner et al. 2017), with the latter using 3‐OHK in both eyes and wings for mate recognition. Since our pigment extraction was optimized for pterins, we do not have a representative pigment profile for ommochromes in firefly LOs and eyes (Appendix 2.3: Appendix S9). However, we detected much higher levels of ommochromes in eyes than in LOs (Figure S13), and 3‐OHK was present in LOs only in trace amounts. A pigment analysis optimized for ommochromes will be needed to test and quantify the presence of all ommochrome pigments in firefly LOs and eyes, including those generated by nonenzymatic reactions.

In the pterin pathway, upregulated photic genes included *DhpD2* and *sepia1. DhpD* encodes dihydropterin deaminase that transforms 7,8‐dihydrobiopterin into 7,8‐dihydrolumazine (yellow). In turn, 7,8‐dihydrolumazine can undergo nonenzymatic condensation into aurodrosopterin (orange; Figure 3). The production of yellow 7,8‐dihydrolumazine in both LOs and eyes of *P. pyralis* is supported by our pigment analysis, but we found no evidence of aurodrosopterin production (Table S22). Aurodrosopterin and its isomer, isoaurodrosopterin, are produced by the nonenzymatic condensation of 7,8‐dihydrolumazine with pyrimidodiazepine (PDA) under acidic conditions (Yim et al. 1993).

The second upregulated photic gene in the pterin pathway, *sepia*, encodes pyrimidodiazepine synthase, which converts 6‐pyruvol‐tetrahydropterin (PTP; Figure 3) into pyrimidodiazepine (Kim et al. 2006). This reaction also requires thioredoxin reductase, the product of *clot* (Giordano et al. 2003; Wiederrecht et al. 1984). Pyrimidodiazepine, together with 7,8‐dihydrolumazine, can condense into several orange and red pigments: aurodrosopterin, drosopterin, or their isoforms (Schwinck 1975; Rokos and Pfleiderer 1975; Ayling et al. 2012; Andrade and Carneiro 2021). *Clot* was expressed in both photic tissues, raising the possibility it could function with *sepia* to produce pyrimidodiazepine and the orange‐red pterins; however, we did not detect any in our pigment extracts (Table S22).

Two other pterins, *rosy3* and *mal*, seem to play key roles in LOs because they were exclusively upregulated in LOs relative to the thorax. The *rosy* locus encodes the enzyme xanthin dehydrogenase (XDH), which is involved in several enzymatic reactions, such as the transformation of pterin (colorless) into isoxanthopterin (colorless), of 7,8 dihydropterin into the yellow precursor of xanthopterin (H2‐xanthopterin), and of xanthopterin (yellow) into leucopterin (white), as well as the production of uric acid (Hilliker et al. 1992; Rizki and Rizki 1962), which forms a large reflector layer in the firefly LO to increase the intensity of emitted light (Goh et al. 2013). Leucopterin, a white pigment, underlies the white appearance of *Pieris* butterfly wings (Wijnen et al. 2007). Importantly, *mal* encodes the molybdenum cofactor sulfotransferase required for *rosy* (pterin) activity (Finnerty et al. 1979; Browder et al. 1982; Forrest et al. 1961; Glassman and Mitchell 1959). *Drosophila* mutants without *rosy* and *mal* activity had brown eyes due to a lack of purines and pterins (Hubby and Forrest 1960), signifying their effect on red coloration (Reaume et al. 1989), which could potentially influence LOs. Despite the significant differential expression of *mal* and two copies of *rosy* (*rosy1, rosy3*) between LO and thorax, LC–MS did not recover drosopterins or any substrates with red coloration. It is possible our extraction did not effectively extract drosopterins, so further examination of pterins in fireflies is needed to confirm their absence.

### 4.2 Pigments in Head Shield Coloration

*Photinus* fireflies use conspicuous pink coloration with black and yellow contrasts on their head shield (HS) as an aposematic signal, advertising toxicity (lucibufagins) to diurnal predators (Meinwald et al. 1979; Day 2011). Our computational analysis has helped to clarify the potential genetic basis that may contribute to this essential trait in fireflies. Overall, 44 shared pigment genes were expressed in HS, LOs, and eyes (Table S6), supporting the possibility that gene expression underlying pigmentation is shared across colored tissues. Among the 90th percentile of highly expressed (HE) genes in HS were 10 pigment genes, including ommochromes and pterins, as well as the granule genes *pink* and *mauve*. *Drosophila* with mutant *mauve* appeared to have increased abundance of ommochromes relative to pterins, as well as pigment granules of abnormal size (Rahman et al. 2012). Yellow ommochromes appear to play an important role in HS coloration, as *vermilion* and *cinnabar2* were HE and are involved in the production of 3‐OHK (yellow). The next step of the ommochrome pathway requires both *scarlet* and *white* to transport 3‐OHK into ommochrome granules; however, *scarlet* was not expressed in HS, suggesting additional modification of 3‐OHK into red and/or purple pigments does not occur (Figure 3, Tables S6 and S15). Interestingly, we did not recover 3‐OHK in HS from our preliminary analysis (Table S23), so additional work is needed to verify the contribution of 3‐OHK to HS coloration.

In the pterin pathway of *Drosophila*, *white* also dimerizes with *brown* to import GDP into granules (Figure 3). Though *white1* was in the top 10% of HE genes in HS, we did not recover a *brown* ortholog in *P. pyralis* , possibly due to evolutionary divergence between *Drosophila* and *Photinus* (Grubbs et al. 2015). It is therefore possible that a different ABC transporter dimerizes with *white1* for pterin synthesis in fireflies. (Additional information on the proposed red coloration of *P. pyralis* HS in Appendix 2.5: Appendix S9). Insects are known to redeploy pigment expression networks across tissues and developmental stages, resulting in intraspecific variation (Wittkopp et al. 2002; Vargas‐Lowman et al. 2019) and the evolution of novel traits involved with coloration (Reed and Nagy 2005; Ferguson and Jiggins 2009; Monteiro 2012; Martin et al. 2014). Both *cinnabar2* and *rosy3* were HE in HS (Table S15) and upregulated in LO relative to thorax (Contrast 2), so it is possible that *P. pyralis* body coloration is determined by the varying quantities of the same pigment types. If this is the case, altering expression levels across tissues may reshape trait variation in meaningful ways, even under genetic constraint at the protein level.

### 4.3 Integrating Networks With Gene Expression and Pigments

Our LO gene network analysis supports the importance of diel changes for gene expression in *P. pyralis* that was evident from our gene expression (and pigment) analysis. Our investigation revealed three gene modules significantly associated with LO activity. The genes in both M‐33 (“skyblue3”) and M‐12 (“green”) were more highly expressed in active LOs, but only M‐12 contained pigment genes (three genes from the pterin pathway: *DhpD1*, *sepia1*, and *clot*). Other copies of these genes (*DhpD2, sepia3*) were present in M‐13 (“greenyellow”), which was not associated with the LO state, suggesting that different copies indeed may vary in function. The combined evidence of elevated copy numbers (Table 1, Figure S5) and their expression divergence across tissues and activity states (Table S7), combined with their co‐expression with different gene networks (modules), further supports our hypothesis that these genes may have undergone duplication and neofunctionalization in different firefly tissues. This will serve as an important direction for future studies on the origin of new genes in fireflies.

In contrast, genes in M‐24 (“pink”) were more highly expressed in inactive LOs, including four pigment genes: *scarlet*, *cardinal*, *lightoid*, and *pink* and two genes in the ommochrome pathway (*scarlet* and *cardinal*). *Scarlet* forms a dimer with *white* to transport 3‐OHK from the cytoplasm into granules, which could be elevated during inactivity, possibly to protect firefly tissues from oxidative stress and the potential neurotoxic effects of 3‐OHK (Hughes et al. 2022) while fireflies are resting during the day.

Pigment molecules are influenced by processes outside their direct biosynthesis pathway and are packaged into granules that can be transported to distinct cellular locations. Genes involved in the biogenesis of pigment granules were also part of M‐24 (“pink”). Pigment granules are considered lysosomal‐related organelles because they share characteristics with lysosomes, including acidified lumens (Dell'Angelica et al. 2000; Ohkuma and Poole 1978) and their formation process (Luzio et al. 2014). *Pink* encodes a subunit of the biogenesis of lysosome‐related organelles complex (BLOC), which is implicated in the formation of screening pigments (Falcón‐Pérez et al. 2007) and is critical for body coloration. In *Drosophila, pink* mutants displayed a decline in the abundance of red and brown pigments compared with wildtype (Falcón‐Pérez et al. 2007). The decrease in these pigments was compounded by loss of function in the rab GTPase *lightoid* (Ma et al. 2004; Falcón‐Pérez et al. 2007), which was highly upregulated in both photic tissues in this study (Contrast 2). *Lightoid* plays a key role in the pigment migration, which insects use to enhance vision (Stavenga and Kuiper 1977). In *Drosophila* eyes, *lightoid* interacts with pigment granules (Fujikawa et al. 2002; Satoh et al. 2008) by linking granules to the myosin V motor for transport along actin filaments of the cytoskeleton (Satoh et al. 2008). Whether *lightoid* could trigger pigment granule transport in the firefly eye (Horridge 1969) or even LO remains a question for future investigation.

As with modules “green” (M‐12) and “skyblue3” (M‐33), in “pink” (M‐24) we observed genes related to phototransduction, including four copies of *retinol‐binding protein pinta‐like* (Table S17). We additionally noted genes that play a role in eye development and/or maintenance: *homeobox protein OTX1‐like|homeobox protein OTX2‐like* (PPYR_13979), *chaoptin‐like* (PPYR_14596), *rab11 family‐interacting protein 2* (PPYR_05444), and *ras‐related protein Rab‐28‐like* (PPYR_12570). Module “pink” (M‐24) was also enriched for photic genes, which were upregulated in both LO and eyes relative to thorax (Table S19) and were associated with the “retinal metabolic process” GO term (Table S20). Surprisingly, this module contained four copies of *retinol‐binding protein pinta‐like* (Table S17). *Pinta* is integral for vision in *Drosophila* and is expressed in retinal pigment cells (Wang and Montell 2005), raising questions about why genes characteristic of eyes are associated with inactive LO and if this transcription has functional consequences, e.g., the timing of the observed LO hue change during twilight. It is possible that genes related to circadian rhythm, which relies upon light perception (Wijnen et al. 2006; Yoshii et al. 2016) mediate physiological shifts in activity state. Importantly, *luciferin sulfotransferase* (PPYR_00003), as well as four other LO genes from Fallon et al. (2018), were also present in M‐24, indicating that members of the ommochrome pathway and granule group are regulated alongside genes essential for light signaling. As pigment biosynthesis pathways are complex with interrelated components, modules provide important insight on genes and cellular processes associated with signaling. Collectively, these results point to even greater complexity of pigment production, modification, and localization via multiple interacting factors in firefly signaling.

### 4.4 What Generates Light Color Variation in *P. pyralis* Fireflies?

While we identified relatively few genes that were differentially expressed between yellow and green LOs (Prediction 4), an astonishing number of pigment genes in the ommochrome and pterin pathways (46) were expressed in both LOs and eyes (Prediction 1), with some variation in expression levels. It is possible that small differences in gene expression may not yield effect sizes large enough for statistical significance (e.g., 7 LO samples). If so, increasing the sample size in future studies may reveal even greater genetic variation underlying differences in light color or activity state.

In the photic tissues of active fireflies, we observed both the expression of pterin genes *DhpD* and *sepia*, as well as their pigment products. Three copies of *DhpD2* and *sepia1* each were expressed in *P. pyralis* , and both pterin genes were significantly upregulated in both active LOs and active eyes relative to the active thorax (Contrast 2), implicating them as possible candidates for tuning between light color and visual sensitivity (Lall et al. 1988). The importance of *DhpD* to signaling is further supported by the observation that *DhpD1* was upregulated in active LOs compared to their inactive state (Contrast 3) and part of module “green” (M‐12), which was associated with active LOs.

The pterin product of *DhpD* is the yellow (possibly pink; Yim et al. 1993) pigment 7,8‐dihydrolumazine, which was one of the most abundant pterins in *P. pyralis* LOs and eyes. This may constitute the mysterious “lampyrine” pigment described by Metcalf (1943) and could also be used for tuning *P. pyralis* visual sensitivity (Lall et al. 1988). Further, our pigment analysis suggests a trend in 7,8‐dihydrolumazine levels, which were relatively higher in the LOs and eyes of yellower fireflies compared with greener fireflies. The product of *sepia* is pyrimidodiazepine (Kim et al. 2006), which can condense with 7,8‐dihydrolumazine into several orange and red pigments (aurodrosopterin, drosopterin, or their isoforms), underscoring the importance of these colored pigments for light production and detection.

In addition to differences in gene expression encoding pigment synthesis, we identified potential modes for pigment modification and interaction with other pathways in LOs. It is possible that pigments in LOs and eyes are altered by changes in the intracellular conditions. Specifically, shifts in the expression of genes that regulate pH or redox state can cause structural changes to pigments that affect how they interact with light. For example, ommatins reflect different light colors based on their redox state (Huang et al. 2024), and in male dragonflies, xanthommatin (yellow) is reduced to dihydroxanthomatin (red) under acidic conditions, resulting in a body color change from yellow to red (Futahashi et al. 2012). Such changes can also affect the rate of nonenzymatic reactions, as the condensation rate of 7,8‐dihydrolumazine with pyramidodiazepine to aurodrosopterins increased with low pH (Yim et al. 1993; Kim et al. 2009). Among genes upregulated only in LO (Contrast 2), we identified DEG with annotations associated with cellular pH, as well as several GO terms related to pH (i.e., proton transmembrane transporter; Table S12). Our KEGG analysis revealed that within the “lysosome” pathway, genes encoding acidification regulators were upregulated in LO relative to thorax (Appendix 2.4: Appendix S9, Table S8, Figure S15). Importantly, the M‐27 (“red”) module (marginally significant for its association with inactive LOs) was enriched for LO genes from Fallon et al. (2018). Within M‐27, we observed GO terms for vacuolar acidification, pH reduction, and response to alkaline pH, suggesting a strong association between genes related to pH and light signaling, including luciferase (Tables S17–S20, Figure S10). Such shifts in regulation between activity states could modify pigments present in LOs and their subsequent filtering effect and possibly mediate the hue change. Hence, we suggest that multiple cellular processes and interactions contribute to signal color.

Extensive experimentation with firefly luciferase has shown that *P. pyralis* has a pH‐sensitive luciferase (Viviani et al. 2022), meaning the light spectrum shifts to red wavelengths (> 600 nm) when pH or heavy metal concentrations are altered (Viviani et al. 2022). Could light color variation in *P. pyralis* be achieved by manipulating pH and luciferase light emission, rather than changing the pigment filter in light organs? Current evidence suggests that this is not the case. Under varied pH conditions presented by Viviani et al. (2018), the in vitro light spectra of *P. pyralis* luciferase display nearly identical peaks in the yellow‐green range at pH 6.5 and 7.0; however, at pH 8 the peak shifts entirely into the red range. Interestingly, the peak broadens substantially at pH 7.5, encompassing wavelengths from yellow to red (Ando et al. 2008; Viviani et al. 2018). Similar spectral distributions are observed when luciferase is exposed to Zn^++^ (Seliger and McElroy 1964) and temperatures > 34°C (Rabha et al. 2021) but have not yet been observed in fireflies signaling under natural conditions (Hall et al. 2016, as well as this study). Further, the red shift resulting from these manipulations was accompanied by a substantial reduction in light intensity (Seliger and McElroy 1964; Ando et al. 2008; Rabha et al. 2021; Viviani et al. 2022), possibly due to denaturation of luciferase (Rabha et al. 2021). As mating success is linked to brighter signals (Vencl and Carlson 1998; Cratsley and Lewis 2003; Lewis and Cratsley 2008), these mechanisms would likely reduce fitness, suggesting in vivo firefly light color is more complex than it would appear from in vitro manipulations. Based on the luciferase light spectra recorded during these manipulations, it is more likely that the pH‐related gene expression in firefly LOs contributes to pH homeostasis and does not alter luciferase light production. However, cellular sub‐compartmentalization of the bioluminescent reaction (e.g., luciferase) inside peroxisomes could potentially allow for shifts in pH within the cytoplasm (where pigment precursors are located) and/or pigment granules.

Temperature can also influence flashing activity, though it primarily affects flash duration and repetition rate (Lloyd 1966). Rabha et al. (2021) used a laboratory experiment to show that extreme temperatures (below 10.5°C and above 34°C) could shift the light spectra of the Asian firefly (*Sclerotia substriata*) towards red wavelengths. *Photinus* fireflies tend to stop flying and signaling below 12.78°C, and a previous study (Hall et al. 2016) detected no influence of temperature on the light spectra of *P. scintillans* fireflies. Similarly, our light spectra were recorded between 20.6°C and 32.2°C (Iowa Environmental Mesonet, Table S24), and there was no evidence of a correlation between light color and temperature (Appendix 1.2: Appendix S9, Figure S17, Table S25).

Finally, the observation that *P. pyralis* light organs change hue approximately 15 min prior to their activity onset suggested that transporters play an important role in this transition. Transporters import pigment precursors into the pigment granules, where both enzymatic and nonenzymatic reactions may occur. However, transporter expression was consistent across activity states, implying that transporters are always active and that the hue change is more likely triggered by a substrate‐driven change in transport rate (which can be reversed when fireflies are captured or become inactive). This rapid physiological change could also be triggered by a pH shift that modifies the balance of nonenzymatic pigment reactions and their color. Alternatively, there is evidence that by binding to proteins or metals, pigments can be stabilized by preventing interactions with other molecules, despite changes in pH (Figon et al. 2021). In this case, the hue change at the beginning of activity could be due to the migration of colored pigment granules in photocytes, such as changes from a diffuse to concentrated distribution below the ventral LO surface. Studies of firefly granules in LOs indicate that granules are located within the photocytes (Ghiradella and Schmidt 2004), but *lightoid*, a granule gene involved in pigment migration, was identified as a member of M‐24 (“pink”), which was significantly associated with inactive light organs. Screening pigments in insect eyes migrate daily as an adaptation to changing light conditions (Bernhard et al. 1963; Kirschfeld and Franceschini 1969; Ro and Nilsson 1993). Since *lightoid* was highly upregulated in both active LOs and eyes, it raises the question of whether *lightoid* could play a role in the positioning of pigment granules within LOs and change the filter for the emitted light. If *lightoid* does have pleiotropic effects, it would offer a potential genetic mechanism for co‐evolutionary changes in eyes and LOs of fireflies in a single gene. For more on the potential mechanisms that could influence emitted light color variation, see Figure S18.

## 5 Summary

Fireflies produce pigments in their LOs and eyes that could tune light color to the visual sensitivity of their mates. Using an integrated approach of combining gene expression data with a whole transcriptome computational analysis, and complementing these with preliminary pigment data, we provide evidence that almost all pigment genes in the ommochrome and pterin pathways are expressed in *P. pyralis* LOs and eyes (Predictions 1 and 2), including those with the potential to shift the emitted and/or detected light color (Prediction 3). While we could not identify differentially expressed genes between fireflies that emit green and yellow light colors (Prediction 4), our analysis suggests several pigments and influences on pigment properties that could shift the emitted light towards yellow in fireflies with yellow signals. The same pigments may also shift the visual sensitivity towards yellow and could be involved in the tuning between LOs and eyes. We discuss several alternative explanations for how pigments can influence light color that emerged from our data, including the influence of pH and temperature. We suggest several mechanisms underlying the rapid hue change of LOs at the onset of activity, including a compartmentalized pH change and/or a relocalization of granules. To further examine the role of pigments in firefly signals, future studies should use an extended sampling scheme to investigate additional firefly species whose signals span the range of firefly light color.

Firefly signals evolve to be more conspicuous to potential mates against the signaling background (Lall et al. 1980; Endler 1992). Twilight‐active firefly populations that signal in forest environments (i.e., against a green‐dominated ambient light background) produce significantly more yellow flashes than those in open environments (Hall et al. 2016). Here, we present a pigment mechanism that contributes to the evolution of yellower light color by modifying both the signal color and visual sensitivity of the twilight‐active firefly *P. pyralis* . Adding additional firefly species to such a pigment analysis will not only expand our knowledge on the pigment pathways involved, but also whether different pigments may be under selection for twilight‐ versus dark‐active fireflies, and for populations active in different habitats. Signal divergence in different signal environments can lead to character displacement between populations (Stanger‐Hall and Lloyd 2015), and possibly to speciation; the role that pigments play in this process remains to be seen.

## Author Contributions

**M. S. Popecki:** conceptualization (equal), data curation (lead), formal analysis (lead), investigation (lead), methodology (equal), visualization (lead), writing – original draft (lead). **S. A. Archer‐Hartmann:** formal analysis (supporting), resources (supporting). **P. Azadi:** resources (supporting). **R. L. Rogers:** funding acquisition (equal), investigation (supporting), methodology (equal), project administration (equal), supervision (equal), writing – review and editing (equal). **J. P. Wares:** formal analysis (equal), methodology (equal), project administration (equal), resources (equal), supervision (equal), writing – review and editing (equal). **K. F. Stanger‐Hall:** conceptualization (lead), funding acquisition (equal), methodology (equal), project administration (lead), resources (lead), supervision (equal), writing – review and editing (equal).

## Conflicts of Interest

The authors declare no conflicts of interest.

## Supporting information


**Appendix S1:** ece371927‐sup‐0001‐AppendixS1.txt.

**Appendix S2:** ece371927‐sup‐0002‐AppendixS2.txt.

**Appendix S3:** ece371927‐sup‐0003‐AppendixS3.txt.

**Appendix S4:** ece371927‐sup‐0004‐AppendixS4.txt.

**Appendix S5:** ece371927‐sup‐0005‐AppendixS5.txt.

**Appendix S6:** ece371927‐sup‐0006‐AppendixS6.txt.

**Appendix S7:** ece371927‐sup‐0007‐AppendixS7.txt.

**Appendix S8:** ece371927‐sup‐0008‐AppendixS8.txt.

**Appendix S9:** ece371927‐sup‐0009‐AppendixS9.docx.

**Appendix S10:** ece371927‐sup‐0010‐AppendixS10.xlsx.

## Data Availability

SRA: PRJNA1047132. Supplemental data and protocols available at https://github.com/mspopecki/Gene‐expression‐Photinus‐pyralis.git.
