# Supplementary material for: The Role of Pigments in Light Color Variation of the Firefly Photinus pyralis
Source: Ecol Evol. 2025 Aug 19;15(8):e71927. doi: 10.1002/ece3.71927 (PMC12364529; doi:10.1002/ece3.71927)
Supplement: Supplementary file 9 — Appendix S9: ece371927‐sup‐0009‐AppendixS9.docx. [file ECE3-15-e71927-s001.docx]

**Appendix**

**1. Supplemental Methods**

1.1 Molecular species confirmation with COI………………………………………………….....3

1.2 Correlation between light color and temperature……………………………………………...3

1.3 Verify luciferase amino acid sequences are identical…………………………………………4

1.4 PCA of pterin substrates from LC-MS……………………………………………………......4

1.5 Welch’s T-test between pterin substrates and light color……………………………………..4

**2. Supplemental Results**

2.1 Modules associated with light color…………………………………………………………..5

2.2. Module “Red” (M-27) and bioluminescence…………………………………………..……..5

2.3 Insights on ommochromes from LC-MS..………………………………...…………………..5

2.4 KEGG Enrichment of light organs and eyes (vs. thorax)………………. …..………….…….6

2.5 Head shield coloration………………………………………………………………………...7

2.6 Intraspecific light color variation in *P. pyralis*………………………………………………..8

**3. Supplemental Figures**

S1. Sampling sites………………………………………………………………………………...9

S2. Network analysis with WCGNA…………………………………………………….………10

S3. *Cytochrome oxidase subunit I* (COI) gene tree………………………………………….…..11

S4. Alignment of luciferase sequences derived from light organ transcriptomes…………….....13

S5. Copy number of orthologous pigments sequences across tissues………………………...…14

S6. Gene tree of white/scarlet gene family…………………………………………………...….15

S7. PCA of gene expression using VST-transformed counts…………………………………....16

S8. Expression of *rosy1* (outlier)………………………………..……………………………….17

S9. Modules associated with light color of *P. pyralis* light organs (marginally significant)…....18

S10. Heatmap of Module-27 “Red”……………………………………………………………...19

S11. Absorbance measurements of pterin pigments from UV-vis…………………………...….20

S12. PCA Biplot of the 15 pterin substrates recovered in *Photinus pyralis* tissue samples…..…21

S13. Relative abundance of pigments in active LOs and eyes (different light colors)…………..22

S14. Relative abundance of pigments in active and inactive yellow LOs and eyes……………..23

S15. KEGG pathway enrichment of genes upregulated in active LO (vs. thorax)………………24

S16. KEGG pathway enrichment of genes upregulated in active eye (vs. thorax)………………25

S17. No correlation between light color and temperature……………………………………….26

S18. Possible mechanisms for how pigments influence light color……………………………...27

**4. Supplemental Tables**

***Located in Excel File: “Supplemental_Tables_Role_of_pigments_Photinus_pyralis_light_color.xlsx”***

S1. Mean light color of *P. pyralis* fireflies used for transcriptome analysis.

S2. *P. pyralis* fireflies used for pterin pigment isolation.

S3. Quality report of samples used in transcriptomic analysis.

S4. Proteomes used as input for OrthoFinder.

S5. List of pigment genes used to identify *P. pyralis* orthologs.

S6. Mean TPM of pigment gene expression across tissue samples.

S7. Pigment genes with significant differential expression in *P. pyralis*.

S8. Enrichment of KEGG pathways for genes DE between light organ and thorax

S9. Enrichment of KEGG pathways for genes DE between eye and thorax

S10. Expression of LO genes from Fallon et al. (2018) in *P. pyralis* light organs.

S11. GO enrichment of photic genes (Contrast 2).

S12. GO enrichment of LO-specific genes (Contrast 2).

S13. GO enrichment of eye-specific genes (Contrast 2).

S14. Genes upregulated in LO (vs. TX, Contrast 2) and upregulated in active LOs (Contrast 3)

S15. Pigment genes expressed in the head shield transcriptome (including top 10%).

S16. Number of genes in all modules.

S17. List of genes in all modules.

S18. Significant "hubs" associated with light organ activity state.

S19. Testing modules for enrichment for gene lists.

S20. Gene ontology of all modules.

S21. Pterin substrates searched for extracts analyzed by LC-MS.

S22. LC-MS results for pterin substrates.

S23. LC-MS results for pterin with ommochrome substrates.

S24. Temperature records for all samples collected.

S25. List of all firefly samples used to test for correlation between light color and temperature.

**5. Works Cited**

References…………………..……………………………………………………………………30

**1. Supplemental Methods**

**1.1 Molecular species confirmation with COI**

To verify our species identification in the field (based on flash pattern and morphological features), we isolated DNA from thorax muscle tissue (Qiagen, Germantown, MD) for COI sequencing. We used LCO and HCO primers (Folmer et al., 1994) to amplify a 620 bp segment of the COI gene. PCR products were cleaned with column and enzymatic methods to remove contamination of residual reagents (Qiagen) and submitted for Sanger sequencing (GeneWiz; South Plainfield, NJ). Raw sequences were assembled and edited (to verify and/or remove poor quality bases) using Geneious v2022.22.2. We aligned sequences (MUSCLE), including an outgroup species *Pyractomena borealis* (Genbank EU009269.1) and *Photinus pyralis* specimen with confirmed species identification (accession KP121616.1), obtained from GenBank. To verify species in our sample, we estimated a COI gene tree (neighbor-joining) in Geneious v2022.22.2 and confirmed that our firefly specimens clustered in clades with a verified representative of that species (Figure S3). To verify individuals were not diverging evolutionarily, we estimated Hudson’s Snn statistic genetic differentiation of subpopulations (Hudson, 2000). Sequences are deposited at NCBI (accession numbers SAMN38520475-38520511). Mitochondrial (Snn=0.5; p ~1) and additional whole-genome re-sequencing data confirmed no measurable evolutionary divergence of these populations (manuscript forthcoming).

**1.2 Correlation between light color and temperature**

Temperature was shown to modify bioluminescence color in two species of Indian fireflies (e.g., Rabha et al., 2021). In these species, temperatures above 34° C caused light color to shift from yellow-green to redder wavelengths in constrained fireflies. This has not been observed in *Photinus* *scintillans* fireflies (Hall et al., 2016). While there was variation in peak wavelength between individual fireflies, there was no trend in peak wavelength as the evening progresses in *P. carolinus* fireflies, implying that light color does not change during their activity period (Hall et al., 2016 data from Allegheny National Forest).

For our study with *P. pyralis,* we retrieved temperature records from Iowa Environmental Mesonet (Iowa State University, 2001) for 3-5 time points during firefly activity (19:51-21:51) during all eight evenings of field work from the Athens municipal station (-83.3264, 33.9486). The temperature range for all sampling was 20.56-32.22°C (mean=26.875**±**3.313956 SD °C). Temperatures were cooler at Flint St, from 20.56 - 26.67°C (mean=24.84321**±**2.462488 SD °C, N=4 evenings) compared with Rose Creek, which ranged from 26.11- 32.22°C (mean=28.90679 **±**2.830252 SD °C, N=4 evenings). Each evening, the temperature was highest at the earliest time (19:51) (Table S24). The highest recorded temperature (Mesonet) was 32.2°C on July 2, 2020 at Rose Creek.

We tested for a correlation between mean light color and temperature taken at the beginning of each evening (17:51) using in base R v4.3.3 using the function cor.test() using a total of 56 samples, including N=27 from this study (N=19: RNA, N=8: pigment) in addition to 29 samples collected at Flint St (N=15) and Rose Creek (N=14) (Table S25). Overall, there was no correlation between light color and temperature (Pearson’s correlation: r= 0.1715562, p=0.2061) (Figure S17).

**1.3 Verify luciferase amino acid sequences are identical**

Previous work (Hall et al., 2016) indicated that light color varies in *P. pyralis* despite identical amino acid sequences. To confirm the uniformity of luciferase and rule out the effects of luciferase on light color variation in our analysis, we used our trimmed RNA reads of all light organ samples (active and inactive, N=8) to assemble reference-guided transcriptomes with Trinity v2.10.0 (Grabherr et al., 2011; Haas et al., 2013) using the *P. pyralis* genome (Fallon et al., 2018). The longest open reading frame was translated with TransDecoder v5.5.0 (Haas et al., 2013). Using the luciferase amino acid sequence available on NCBI (PPYR_00001) we identified the best match in the translated transcriptomes using BLASTP v2.9.0 (NCBI) (e-value: 1E-5). These protein sequences were aligned with PASTA v1.8.5 (Mirarab et al., 2014). Due to low sequence quality (see Methods), one inactive “green” light organ sample (FS34I3) was removed from our analysis resulting in eight specimens in our final alignment. The protein alignment was viewed and manually trimmed in Geneious v2022.22.2 to a consensus length of 562 bp (Figure S4).

**1.4 PCA of pterin substrates from LC-MS**

Using data from LC-MS, we identified 17 pterin substrates (Table S21) based on their retention time (RT) and molecular mass. To calculate relative abundance of pterins, we divided the mass-to-charge ratio (m/z) of each pterin substrate by the total (m/z) of all pterin substrates identified (Table S22), which was used as input for the clustering analysis. We removed two substrates that contained 0 m/z for all samples (dihydroneopterin and aurodrosopterin) resulting in a total of 15 pterin substrates. As we used relative abundances, no additional transformation was used. The PCA biplot (Figure S11) was made in R v4.3.3 using packages prcomp (center and scale=TRUE) and fviz_pca_biplot from factoextra v1.0.7.999 (Kassambara & Mundt, 2017).

**1.5 Welch’s T-test between pterin substrates and light color**

To determine if there were differences in the relative abundances of 15 pterin substrates between light organ samples with extremes in light color (“yellow” vs. “green”), we performed Welch Two-Sample T-test in R v4.3.3. We used a non-parametric analysis to address non-equal variances between pigment substrates of fireflies with yellow or green light. There were no significant differences in pterin substrates with light color (t = 0.1772, df = 27.552, p-value = 0.8606), indicating the pterin profile of fireflies with “yellow” and “green” light color are similar and cannot explain variation in light color alone (given our sample sizes).

**2. Supplemental Results**

**2.1 Modules putatively associated with emitted light color**

To identify modules where gene expression was significantly correlated with light color, we used a Pearson correlation between module expression (ME = PC1 or summary value of module genes) and light color (nm) measured from individual fireflies. Using a significance threshold of p < 0.05, we detected two modules with marginal significance: M-20 (“midnightblue”: p= 0.0508, p-adjusted= 0.8075) and M-30 (“salmon”: p=0.0376, p-adjusted=0.8144) (Figure S9). The genes in both modules were more highly expressed in light organs that produced greener light color.

**2.2 Module “Red” (M-27) and bioluminescence**

The 546 genes in module “red” (M-27) were significantly (p<0.05) associated with inactive light organs, including one pigment gene: sepia5 (pterin). *Sepia5* was expressed in all tissues, with highest expression in the eye (mean TPM±SD: x̄=781.25±208.47, N=16), followed by light organ (x̄= 351.67±101.43, N=15) and thorax (x̄=196.25±98.21, N=4). Notably, 34 bioluminescence genes (of 73 in total) were present in this module (Figure S12), including luciferase (PPYR_00001), which we identified as a hub (Table S18). Indeed, Module “Red” was significantly enriched for LO genes from Fallon et al. (2018) *(*FDR=1.95E-25), in addition to “photic” (FDR= 5.04E-10) and LO-specific (FDR=1.90E-22) DEG (Contrast 2). Also present in this module were genes involved in phototransduction, including: *chaoptin* (PPYR_00272) and *retinol-binding protein pinta-like* (PPYR_01227), as well as those regulating circadian rhythm: *cryptochrome-1-like* (PPYR_08961), *potassium voltage-gated channel protein Shaker* (PPYR_14129), and six copies of *takeout-like* (Table S17). This was consistent with our GO analysis (Table S20), which found circadian rhythm (p-value=0.00120, FDR=1.00000), in addition to terms related to acidity: vacuolar acidification (p-value=0.00024, FDR=0.37144), pH reduction (p-value=0.00024, 1.00000), and response to alkaline pH (p-value=0.00182, FDR=1.00000) in addition to purine ribonucleoside monophosphate metabolism (p-value=0.00136, FDR=1.00000) and purine biosynthesis (p-value=0.00264, FDR=1.00000).

**2.3 Insights on ommochromes from LC-MS**

Since our pigment extracts were optimized for pterins, we did not specifically target ommochromes and their precursors, which require a different extract solution (0.5% HCL in methanol: Llandres et al., 2014; Shen et al., 2020). However, we still detected 8 substrates from the ommochrome pathway, adding up to a total of 25 ommochrome and pterin substrates detected with LC-MS in *P. pyralis* fireflies,(Table S23).

Following the approach used for just pterins, we compared pooled samples (2 fireflies each) of the LOs and eyes of active fireflies that emitted green (x̄=561.46± 0.067 nm), intermediate yellow-green (x̄=563.08±0.311 nm), and yellow (x̄=565.30±0.306 nm) light, as well as a pooled sample (2 fireflies) each of inactive light organs and inactive eyes of fireflies that emitted yellow light color (x̄= 565.080± 0.857 nm).

The most abundant molecule in the ommochrome pathway (Figure 3) in all light organs was kynurenic acid (off-white to light yellow color, Table S21). In addition, *P. pyralis* eyes contained kynurenine (light yellow), 3-hydroxy kynurenine (yellow), and an unknown compound (MH+ 162.0549) (Figure S13). The light organs of active yellow fireflies contained a relatively lower abundance of kynurenic acid (off-white to pale yellow) than the light organs of inactive yellow fireflies (5% vs. 25.4%, Figure S13, Table S23). The eyes of active yellow fireflies contained relatively higher levels of kynurenine (light yellow: 9.3% vs 0.8%), relatively lower levels of kynurenic acid (off-white/light yellow: 5.5% vs 25.4%), and 3-hydroxy kynurenine (yellow: 13.9% vs 31.9%) than the eyes of inactive yellow fireflies (Figure S14, Table S23).

*Photinus pyralis* eyes contained high levels of an unknown compound (MH+ 162.0549), which was also identified in the eyes of noctuid moths *Helicoverpa armigera*, where it had the same distribution as xanthommatin (in wildtype), and was absent in *scarlet* transporter mutants (Khan et al. 2017). The structure and function of this compound remains to be determined and could possibly expose another branch of the ommochrome pigment pathway within ommochrome granules.

In our pooled headshield sample (4 headshields), the most abundant pigment precursor in the ommochrome pathway was kynurenic acid (69.1%) (Figure S13, Table S23).

**2.4 KEGG Pathway Enrichment Analysis: Light organs and eyes (vs. thorax) (Contrast 2)**

To characterize genes differentially expressed between active photic tissues (LO, eyes) with thorax, we performed KEGG enrichment analysis and visualized significant pathways in R with gage v2.52.0 (Luo et al., 2009) and visualized with Pathview v1.42.0 (Luo & [Brouwer](https://scholar.google.com/citations?user=cPpOd0kAAAAJ&hl=en&inst=2365059173406736517&oi=sra), 2013). For DEGs upregulated in either LO or eyes relative to thorax, no pathways survived multiple test correction (Benjamini-Hochberg method) so we considered pathways with p<0.05 “of interest.” There were nine such pathways upregulated in LO relative to thorax (Table S8), including “phagosome” (p-value= 0.00580, q-value= 0.47557), “lysosome” (p-value= 0.02489, q-value=0.63728), “purine metabolism” (p-value= 0.01115, q-value= 0.47557), and “neuroactive ligand receptor” (p-value= 0.02045, q-value= 0.63728) (Figures S15-16).

Enrichment of the “lysosome” pathway could have a role related to pigments, as pigments are lysosomal-related organelles (LROs). We observed the upregulation of genes that encode lysosomal acid hydrolases, as well as membrane proteins and acidification regulators. This was particularly interesting, because intracellular conditions (e.g., pH, redox state, presence of metals and/or proteins) can alter the molecular structure of pigments, resulting in changes to their absorption properties (Figon et al., 2021). We also observed the upregulation of genes encoding proteins associated with lysosomal trafficking, which contribute to the biogenesis of pigment granules. Notably, this included the AP-3 complex, whose subunits are encoded by members of the granule group *garnet* (Simpson et al., 1997), *ruby* (Kretzschmar et al., 2000; Mullins et al., 2000), *carmine* (Mullins et al., 1999), and *orange* (Mullins et al., 2000).

Purines are involved in a multitude of roles, including biosynthesis of pterins. The purine degradation pathway gives rise to pterins through incorporation guanine (GTP) into the pigment granule; however, guanine can also be directly converted to xanthine, which results in the final breakdown product, uric acid (Vogels & Van der Drift, 1976). Fireflies have abundant levels of uric acid in their LO, which forms the reflective layer to amplify their light signals outward through the clear cuticle (Goh et al., 2013). It remains unclear whether either of these pathways are indicative of pigments, as they may represent existing pathways that are prominent in LO with a secondary function in pigment synthesis.

We additionally observed “sulfur metabolism” (p-value= 0.04221, q-value= 0.67537) and “biosynthesis of unsaturated fatty acids” (p-value= 0.03987, q-value= 0.03987), which are consistent with luciferin synthesis (Fallon et al., 2016) and luciferase activity (Oba et al., 2003), respectively, as luciferase evolved via gene duplication of fatty acyl-CoA synthase (Fallon et al., 2018).

Among genes upregulated in eyes relative to thorax, we observed the enrichment of “phototransduction” (p-value= 0.02658, q-value= 1.0000), as expected due to their use invision, as well as “Neuroactive ligand-receptor interaction” (p-value= 0.00156, q-value= 0.19992). This pathway was also enriched in active LO, suggesting some similarity in physiological response while signaling.

**2.5 Head shield coloration**

*Photinus* fireflies have clearly recognizable pink pigmentation on their heads shield. Despite the the conspicuousness of this pink pigmentation, we cannot identify any candidates underlying the pink or red component of *P. pyralis* HS. Interestingly, a pink pigment called “lampyrine” shares some characteristics with pterins and is exclusively found across several body tissues in Lampyridae (Metcalf, 1943). Similarly, in *Photinus* eyes, an unknown type of magenta-colored screening pigment, proposed to share qualities with pterins, has been implicated in tuning visual sensitivity (Cronin et al., 2000). We cannot identify which of the pigments (or combination of pigments) in our study would extract as a pink or magenta pigment; however, Yim et al. (1993) precipitated a pink pigment when they produced 7,8-dihydrolumazine by oxidation of 5,6,7,8- tetrahydrolumazine (Yim et al., 1993). This raises the strong possibility that the pink “lampyrine” pigment of fireflies is 7,8-dihydrolumazine.

In HS, our pigment analysis detected xanthopterin/isoxanthopterin (yellow/colorless), leucopterin (white), and 7,8 dihydrolumazine (yellow), the product of *DhpD.* Three copies of both *rosy* and *DhpD* were expressed in HS (Table S15). While all copies of *DhpD* were expressed at lower levels, *rosy1* and *rosy3* were among the genes with highest expression (Table S15), thus pterin biosynthesis may contribute to head shield coloration.

**2.6 Intraspecific light color variation in *P. pyralis***

Our analyses also suggest an interaction in gene expression between light color and activity state. Two modules M-20 (“midnightblue”) and M-30 (“salmon”) were marginally significant for light color (Figure S9), and genes from both modules were more highly expressed in light organs that produced greener light color, mostly because gene expression in green LOs did not change with activity states. In contrast, inactive yellow LOs displayed a similar expression level as green LOs, and active yellow LOs had lower expression levels in both modules, with larger variation between samples (Appendix: Figure S9). This suggests that the difference between active yellow and green LOs emerges when yellow LOs transition from the inactive to the active state, with considerable variation in gene expression, whereas the gene expression in greener LOs does not appear to change substantially. To capture interactions between light color and activity state, an extended multivariate analysis with increased sample sizes will be needed, along with a pigment analysis that also includes standards to quantify pigment levels. It is possible that ommochrome pigments also play an important role in LO pigmentation of *P. pyralis* fireflies, although this awaits to be tested with pigment extracts optimized for ommochromes.

**3. Supplemental Figures**


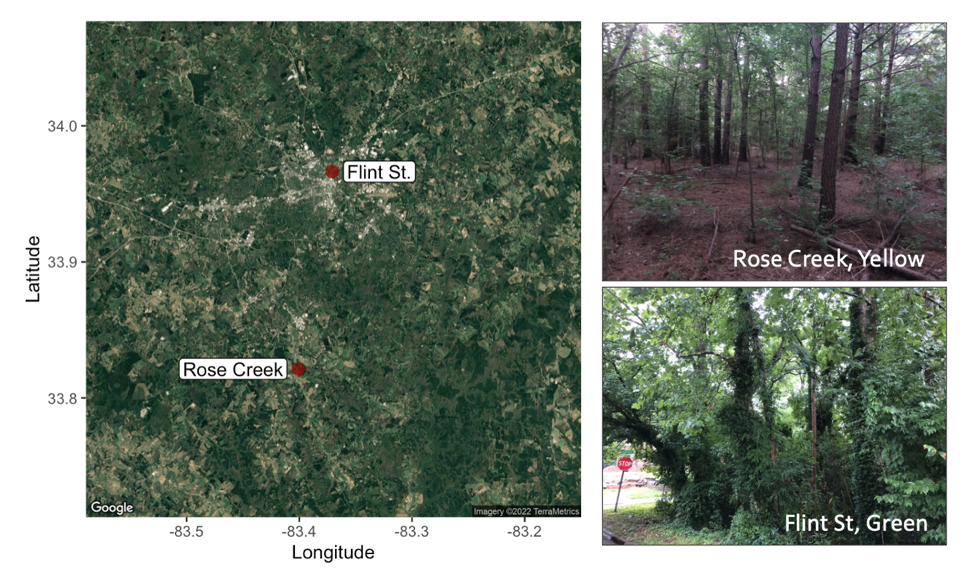


**Figure S1:** Sampling sites. Fireflies with phenotypic differences were collected from two natural populations in Georgia (United States): “green,” Flint St (Athens, GA) and “yellow,” Rose Creek (Watkinsville, GA) located 20 km (straight line distance) apart.


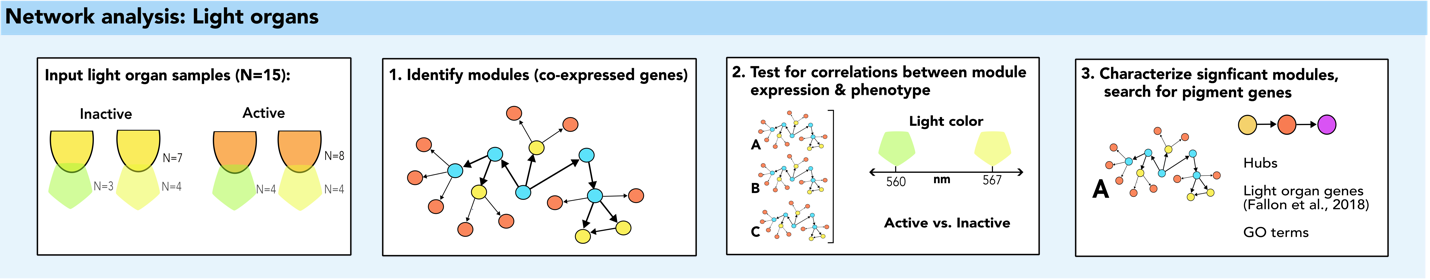


**Figure S2:** Network analysis with WCGNA. To understand patterns of gene expression in light organs, we identified sets of genes with correlated expression (modules) and then tested for correlations between modules and phenotype (activity state, emitted light color). We were particularly interested in modules that were significantly associated with a light organ phenotype (state, light color) and if any pigment genes were present in those modules.


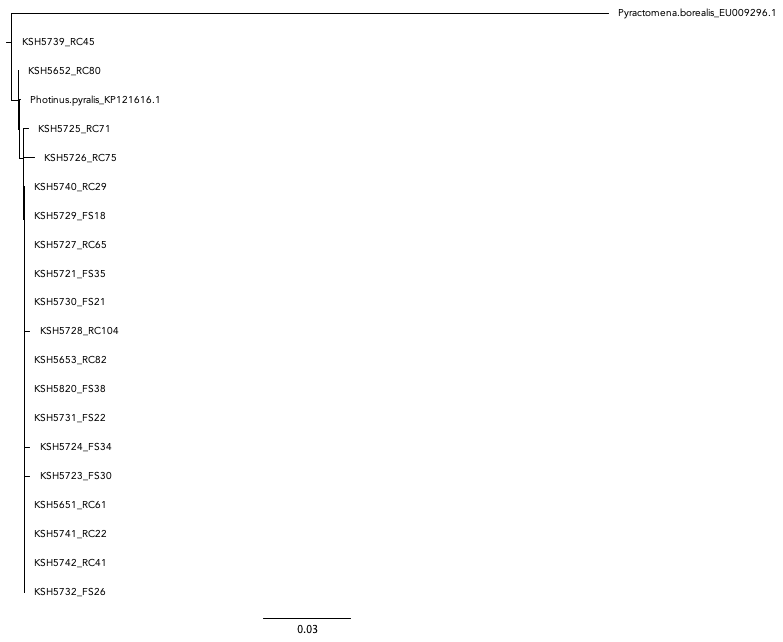


**Figure S3:** *Cytochrome oxidase subunit I* (COI) gene tree from 543 bp alignment. *Pyractomena borealis* (accession EU009296.1) was used as outgroup. Our samples clustered in a clade with the confirmed *Photinus pyralis* sequence (accession KP121616.1), verifying of field species identification for all specimens used in the transcriptome analysis.

Consensus -----------MEDAKNIKKGPAPFYPLEDGTAGEQLHKAMKRYALVPGTIAFTDAHIEV 49

TRINITY_GG_752_c0_g1_i1.p1_FS26A3 -----------................................................. 49

TRINITY_GG_731_c0_g1_i1.p1_RC41A3 -----------................................................. 49

TRINITY_GG_754_c0_g1_i1.p1_RC104I3 -----------................................................. 49

TRINITY_GG_807_c0_g1_i1.p1_RC29A3 -----------................................................. 49

TRINITY_GG_806_c0_g1_i1.p1_RC45A3 -----------................................................. 49

TRINITY_GG_751_c0_g1_i1.p1_FS35I3 -----------................................................. 49

TRINITY_GG_741_c0_g1_i1.p1_FS21A3 -----------................................................. 49

TRINITY_GG_796_c0_g1_i1.p1_FS22A3 -----------................................................. 49

TRINITY_GG_710_c0_g1_i1.p1_FS38I3 -----------................................................. 49

TRINITY_GG_734_c0_g1_i1.p1_RC65I3 -----------................................................. 49

TRINITY_GG_802_c0_g1_i1.p1_RC22A3 -----------................................................. 49

TRINITY_GG_758_c0_g1_i2.p1_RC75I3 -----------................................................. 49

TRINITY_GG_730_c0_g1_i1.p1_FS18A3 -----------................................................. 49

TRINITY_GG_756_c0_g1_i1.p1_FS30I3 -----------................................................. 49

TRINITY_GG_730_c0_g1_i2.p1_RC71I3 -----------................................................. 49

Consensus NITYAEYFEMSVRLAEAMKRYGLNTNHRIVVCSENSLQFFMPVLGALFIGVAVAPANDIY 109

TRINITY_GG_752_c0_g1_i1.p1_FS26A3 ............................................................ 109

TRINITY_GG_731_c0_g1_i1.p1_RC41A3 ............................................................ 109

TRINITY_GG_754_c0_g1_i1.p1_RC104I3 ............................................................ 109

TRINITY_GG_807_c0_g1_i1.p1_RC29A3 ............................................................ 109

TRINITY_GG_806_c0_g1_i1.p1_RC45A3 ............................................................ 109

TRINITY_GG_751_c0_g1_i1.p1_FS35I3 ............................................................ 109

TRINITY_GG_741_c0_g1_i1.p1_FS21A3 ............................................................ 109

TRINITY_GG_796_c0_g1_i1.p1_FS22A3 ............................................................ 109

TRINITY_GG_710_c0_g1_i1.p1_FS38I3 ............................................................ 109

TRINITY_GG_734_c0_g1_i1.p1_RC65I3 ............................................................ 109

TRINITY_GG_802_c0_g1_i1.p1_RC22A3 ............................................................ 109

TRINITY_GG_758_c0_g1_i2.p1_RC75I3 ............................................................ 109

TRINITY_GG_730_c0_g1_i1.p1_FS18A3 ............................................................ 109

TRINITY_GG_756_c0_g1_i1.p1_FS30I3 ............................................................ 109

TRINITY_GG_730_c0_g1_i2.p1_RC71I3 ............................................................ 109

Consensus NERELLNSMNISQPTVVFVSKKGLQKILNVQKKLPIIQKIIIMDSKTDYQGFQSMYTFVT 169

TRINITY_GG_752_c0_g1_i1.p1_FS26A3 ............................................................ 169

TRINITY_GG_731_c0_g1_i1.p1_RC41A3 ............................................................ 169

TRINITY_GG_754_c0_g1_i1.p1_RC104I3 ............................................................ 169

TRINITY_GG_807_c0_g1_i1.p1_RC29A3 ............................................................ 169

TRINITY_GG_806_c0_g1_i1.p1_RC45A3 ............................................................ 169

TRINITY_GG_751_c0_g1_i1.p1_FS35I3 ............................................................ 169

TRINITY_GG_741_c0_g1_i1.p1_FS21A3 ............................................................ 169

TRINITY_GG_796_c0_g1_i1.p1_FS22A3 ............................................................ 169

TRINITY_GG_710_c0_g1_i1.p1_FS38I3 ............................................................ 169

TRINITY_GG_734_c0_g1_i1.p1_RC65I3 ............................................................ 169

TRINITY_GG_802_c0_g1_i1.p1_RC22A3 ............................................................ 169

TRINITY_GG_758_c0_g1_i2.p1_RC75I3 ............................................................ 169

TRINITY_GG_730_c0_g1_i1.p1_FS18A3 ............................................................ 169

TRINITY_GG_756_c0_g1_i1.p1_FS30I3 ............................................................ 169

TRINITY_GG_730_c0_g1_i2.p1_RC71I3 ............................................................ 169

Consensus SHLPPGFNEYDFVPESFDRDKTIALIMNSSGSTGLPKGVALPHRTACVRFSHARDPIFGN 229

TRINITY_GG_752_c0_g1_i1.p1_FS26A3 ............................................................ 229

TRINITY_GG_731_c0_g1_i1.p1_RC41A3 ............................................................ 229

TRINITY_GG_754_c0_g1_i1.p1_RC104I3 ............................................................ 229

TRINITY_GG_807_c0_g1_i1.p1_RC29A3 ............................................................ 229

TRINITY_GG_806_c0_g1_i1.p1_RC45A3 ............................................................ 229

TRINITY_GG_751_c0_g1_i1.p1_FS35I3 ............................................................ 229

TRINITY_GG_741_c0_g1_i1.p1_FS21A3 ............................................................ 229

TRINITY_GG_796_c0_g1_i1.p1_FS22A3 ............................................................ 229

TRINITY_GG_710_c0_g1_i1.p1_FS38I3 ............................................................ 229

TRINITY_GG_734_c0_g1_i1.p1_RC65I3 ............................................................ 229

TRINITY_GG_802_c0_g1_i1.p1_RC22A3 ............................................................ 229

TRINITY_GG_758_c0_g1_i2.p1_RC75I3 ............................................................ 229

TRINITY_GG_730_c0_g1_i1.p1_FS18A3 ............................................................ 229

TRINITY_GG_756_c0_g1_i1.p1_FS30I3 ............................................................ 229

TRINITY_GG_730_c0_g1_i2.p1_RC71I3 ............................................................ 229

Consensus QIIP-DTAILSVVPFHHGFGMFTTLGYLICGFRVVLMYRFEEELFLRSLQDYKIQSALLV 288

TRINITY_GG_752_c0_g1_i1.p1_FS26A3 ....-....................................................... 288

TRINITY_GG_731_c0_g1_i1.p1_RC41A3 ....-....................................................... 288

TRINITY_GG_754_c0_g1_i1.p1_RC104I3 ....-....................................................... 288

TRINITY_GG_807_c0_g1_i1.p1_RC29A3 ....-....................................................... 288

TRINITY_GG_806_c0_g1_i1.p1_RC45A3 ....-....................................................... 288

TRINITY_GG_751_c0_g1_i1.p1_FS35I3 ....-....................................................... 288

TRINITY_GG_741_c0_g1_i1.p1_FS21A3 ....-....................................................... 288

TRINITY_GG_796_c0_g1_i1.p1_FS22A3 ....-....................................................... 288

TRINITY_GG_710_c0_g1_i1.p1_FS38I3 ....-....................................................... 288

TRINITY_GG_734_c0_g1_i1.p1_RC65I3 ....-....................................................... 288

TRINITY_GG_802_c0_g1_i1.p1_RC22A3 ....-....................................................... 288

TRINITY_GG_758_c0_g1_i2.p1_RC75I3 ....-....................................................... 288

TRINITY_GG_730_c0_g1_i1.p1_FS18A3 ....-....................................................... 288

TRINITY_GG_756_c0_g1_i1.p1_FS30I3 ....-....................................................... 288

TRINITY_GG_730_c0_g1_i2.p1_RC71I3 ....-....................................................... 288

Consensus PTLFSFFAKSTLIDKYDLSNLHEIASGGAPLSKEVGEAVAKRFHLPGIRQGYGLTETTSA 348

TRINITY_GG_752_c0_g1_i1.p1_FS26A3 ............................................................ 348

TRINITY_GG_731_c0_g1_i1.p1_RC41A3 ............................................................ 348

TRINITY_GG_754_c0_g1_i1.p1_RC104I3 ............................................................ 348

TRINITY_GG_807_c0_g1_i1.p1_RC29A3 ............................................................ 348

TRINITY_GG_806_c0_g1_i1.p1_RC45A3 ............................................................ 348

TRINITY_GG_751_c0_g1_i1.p1_FS35I3 ............................................................ 348

TRINITY_GG_741_c0_g1_i1.p1_FS21A3 ............................................................ 348

TRINITY_GG_796_c0_g1_i1.p1_FS22A3 ............................................................ 348

TRINITY_GG_710_c0_g1_i1.p1_FS38I3 ............................................................ 348

TRINITY_GG_734_c0_g1_i1.p1_RC65I3 ............................................................ 348

TRINITY_GG_802_c0_g1_i1.p1_RC22A3 ............................................................ 348

TRINITY_GG_758_c0_g1_i2.p1_RC75I3 ............................................................ 348

TRINITY_GG_730_c0_g1_i1.p1_FS18A3 ............................................................ 348

TRINITY_GG_756_c0_g1_i1.p1_FS30I3 ............................................................ 348

TRINITY_GG_730_c0_g1_i2.p1_RC71I3 ............................................................ 348

Consensus ILITPEGDDKPGAVGKVVPFFEAKVVDLDTGKTLGVNQRGELCVRGPMIMSGYVNNPEAT 408

TRINITY_GG_752_c0_g1_i1.p1_FS26A3 ............................................................ 408

TRINITY_GG_731_c0_g1_i1.p1_RC41A3 ............................................................ 408

TRINITY_GG_754_c0_g1_i1.p1_RC104I3 ............................................................ 408

TRINITY_GG_807_c0_g1_i1.p1_RC29A3 ............................................................ 408

TRINITY_GG_806_c0_g1_i1.p1_RC45A3 ............................................................ 408

TRINITY_GG_751_c0_g1_i1.p1_FS35I3 ............................................................ 408

TRINITY_GG_741_c0_g1_i1.p1_FS21A3 ............................................................ 408

TRINITY_GG_796_c0_g1_i1.p1_FS22A3 ............................................................ 408

TRINITY_GG_710_c0_g1_i1.p1_FS38I3 ............................................................ 408

TRINITY_GG_734_c0_g1_i1.p1_RC65I3 ............................................................ 408

TRINITY_GG_802_c0_g1_i1.p1_RC22A3 ............................................................ 408

TRINITY_GG_758_c0_g1_i2.p1_RC75I3 ............................................................ 408

TRINITY_GG_730_c0_g1_i1.p1_FS18A3 ............................................................ 408

TRINITY_GG_756_c0_g1_i1.p1_FS30I3 ............................................................ 408

TRINITY_GG_730_c0_g1_i2.p1_RC71I3 ............................................................ 408

Consensus NALIDKDGWLHSGDIAYWDEDEHFFIVDRLKSLIKYKGYQVAPAELESILLQHPNIFDAG 468

TRINITY_GG_752_c0_g1_i1.p1_FS26A3 ............................................................ 468

TRINITY_GG_731_c0_g1_i1.p1_RC41A3 ............................................................ 468

TRINITY_GG_754_c0_g1_i1.p1_RC104I3 ............................................................ 468

TRINITY_GG_807_c0_g1_i1.p1_RC29A3 ............................................................ 468

TRINITY_GG_806_c0_g1_i1.p1_RC45A3 ............................................................ 468

TRINITY_GG_751_c0_g1_i1.p1_FS35I3 ............................................................ 468

TRINITY_GG_741_c0_g1_i1.p1_FS21A3 ............................................................ 468

TRINITY_GG_796_c0_g1_i1.p1_FS22A3 ............................................................ 468

TRINITY_GG_710_c0_g1_i1.p1_FS38I3 ............................................................ 468

TRINITY_GG_734_c0_g1_i1.p1_RC65I3 ............................................................ 468

TRINITY_GG_802_c0_g1_i1.p1_RC22A3 ............................................................ 468

TRINITY_GG_758_c0_g1_i2.p1_RC75I3 ............................................................ 468

TRINITY_GG_730_c0_g1_i1.p1_FS18A3 ............................................................ 468

TRINITY_GG_756_c0_g1_i1.p1_FS30I3 ............................................................ 468

TRINITY_GG_730_c0_g1_i2.p1_RC71I3 ............................................................ 468

Consensus VAGLPDDDAGELPAAVVVLEHGKTMTEKEIVDYVASQVTTAKKLRGGVVFVDEVPKGLTG 528

TRINITY_GG_752_c0_g1_i1.p1_FS26A3 ............................................................ 528

TRINITY_GG_731_c0_g1_i1.p1_RC41A3 ............................................................ 528

TRINITY_GG_754_c0_g1_i1.p1_RC104I3 ............................................................ 528

TRINITY_GG_807_c0_g1_i1.p1_RC29A3 ............................................................ 528

TRINITY_GG_806_c0_g1_i1.p1_RC45A3 ............................................................ 528

TRINITY_GG_751_c0_g1_i1.p1_FS35I3 ............................................................ 528

TRINITY_GG_741_c0_g1_i1.p1_FS21A3 ............................................................ 528

TRINITY_GG_796_c0_g1_i1.p1_FS22A3 ............................................................ 528

TRINITY_GG_710_c0_g1_i1.p1_FS38I3 ............................................................ 528

TRINITY_GG_734_c0_g1_i1.p1_RC65I3 ............................................................ 528

TRINITY_GG_802_c0_g1_i1.p1_RC22A3 ............................................................ 528

TRINITY_GG_758_c0_g1_i2.p1_RC75I3 ............................................................ 528

TRINITY_GG_730_c0_g1_i1.p1_FS18A3 ............................................................ 528

TRINITY_GG_756_c0_g1_i1.p1_FS30I3 ............................................................ 528

TRINITY_GG_730_c0_g1_i2.p1_RC71I3 ............................................................ 528

Consensus KLDARKIREILIKAKKGGKSKL 550

TRINITY_GG_752_c0_g1_i1.p1_FS26A3 ...................... 550

TRINITY_GG_731_c0_g1_i1.p1_RC41A3 ...................... 550

TRINITY_GG_754_c0_g1_i1.p1_RC104I3 ...................... 550

TRINITY_GG_807_c0_g1_i1.p1_RC29A3 ...................... 550

TRINITY_GG_806_c0_g1_i1.p1_RC45A3 ...................... 550

TRINITY_GG_751_c0_g1_i1.p1_FS35I3 ...................... 550

TRINITY_GG_741_c0_g1_i1.p1_FS21A3 ...................... 550

TRINITY_GG_796_c0_g1_i1.p1_FS22A3 ...................... 550

TRINITY_GG_710_c0_g1_i1.p1_FS38I3 ...................... 550

TRINITY_GG_734_c0_g1_i1.p1_RC65I3 ...................... 550

TRINITY_GG_802_c0_g1_i1.p1_RC22A3 ...................... 550

TRINITY_GG_758_c0_g1_i2.p1_RC75I3 ...................... 550

TRINITY_GG_730_c0_g1_i1.p1_FS18A3 ...................... 550

TRINITY_GG_756_c0_g1_i1.p1_FS30I3 ...................... 550

TRINITY_GG_730_c0_g1_i2.p1_RC71I3 ...................... 550

**Figure S4:** Alignment of luciferase sequences derived from light organ transcriptomes. The inactive green light organ replicate FS34I3 was excluded from the alignment, as it was omitted from our analysis due to small library size. After trimming and removal of adaptor sequences from mRNA reads, reference-guided transcriptomes were assembled with Trinity v2.10.0. The longest open reading frames were translated with Transdecoder v5.5.0. To identify the luciferase sequence, we used blastp v2.9.0 (NCBI) to obtain the best match (e-value cutoff: 1E-5) between the protein sequences and the published *Photinus pyralis* amino acid sequence (PPYR_00001). All proteins were aligned with MUSCLE in Geneious v2022.2.2 with 100% sequence identity (length: 550 bp), confirming that no differences in the luciferase coding sequence could explain variation in light color.


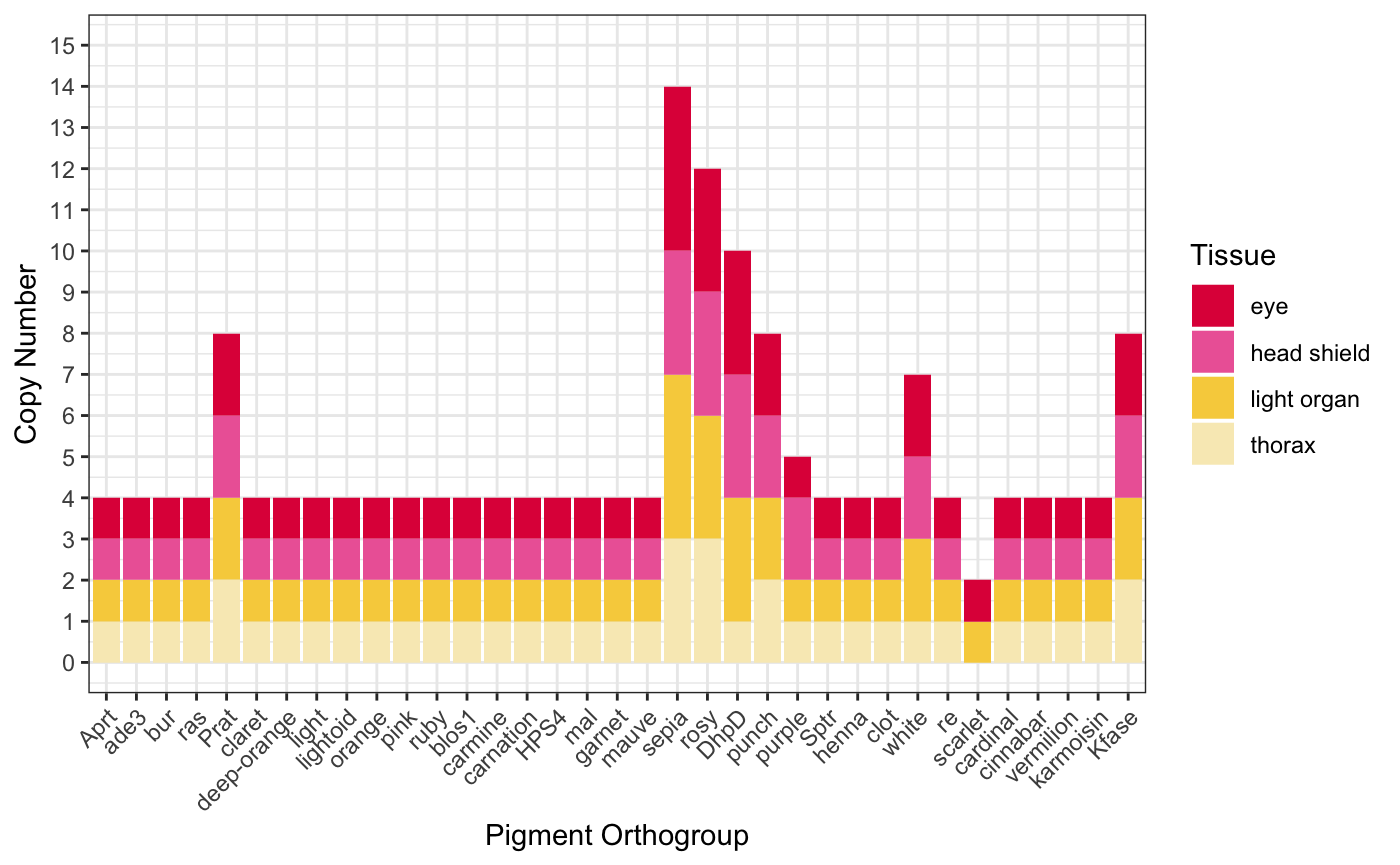


**Figure S5.** Copy number of orthologous pigments sequences across tissues. In *P. pyralis*, pterins appear to have increased copy number compared to other pigment classes.


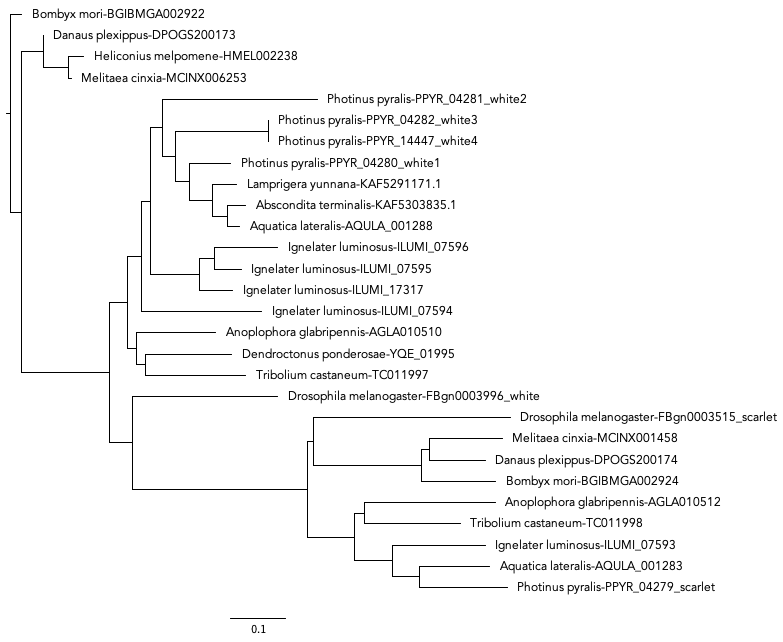


**Figure S6.** Gene tree of white/scarlet gene family. Orthologous protein sequences for each species were aligned with PASTA v1.8.5 which was trimmed with TrimAl v1.4.1 (default parameters with option *-automated1*) prior to maximum likelihood reconstruction with IQ-TREE v1.6.12 (default parameters with option -m MFP). Gene tree was viewed in FigTree v1.4.4. Nodes represent bootstrap support from 1,000 bootstraps. Tree was rooted with the *Bombyx mori* white ortholog BGIBMGA002922 (Komoto et al., 2009). Clustering patterns between the *Drosophila melanogaster* sequences *scarlet* (FBgn0003515) and *white* (FBgn0003515) with *Photinus pyralis* were used to assign orthology.


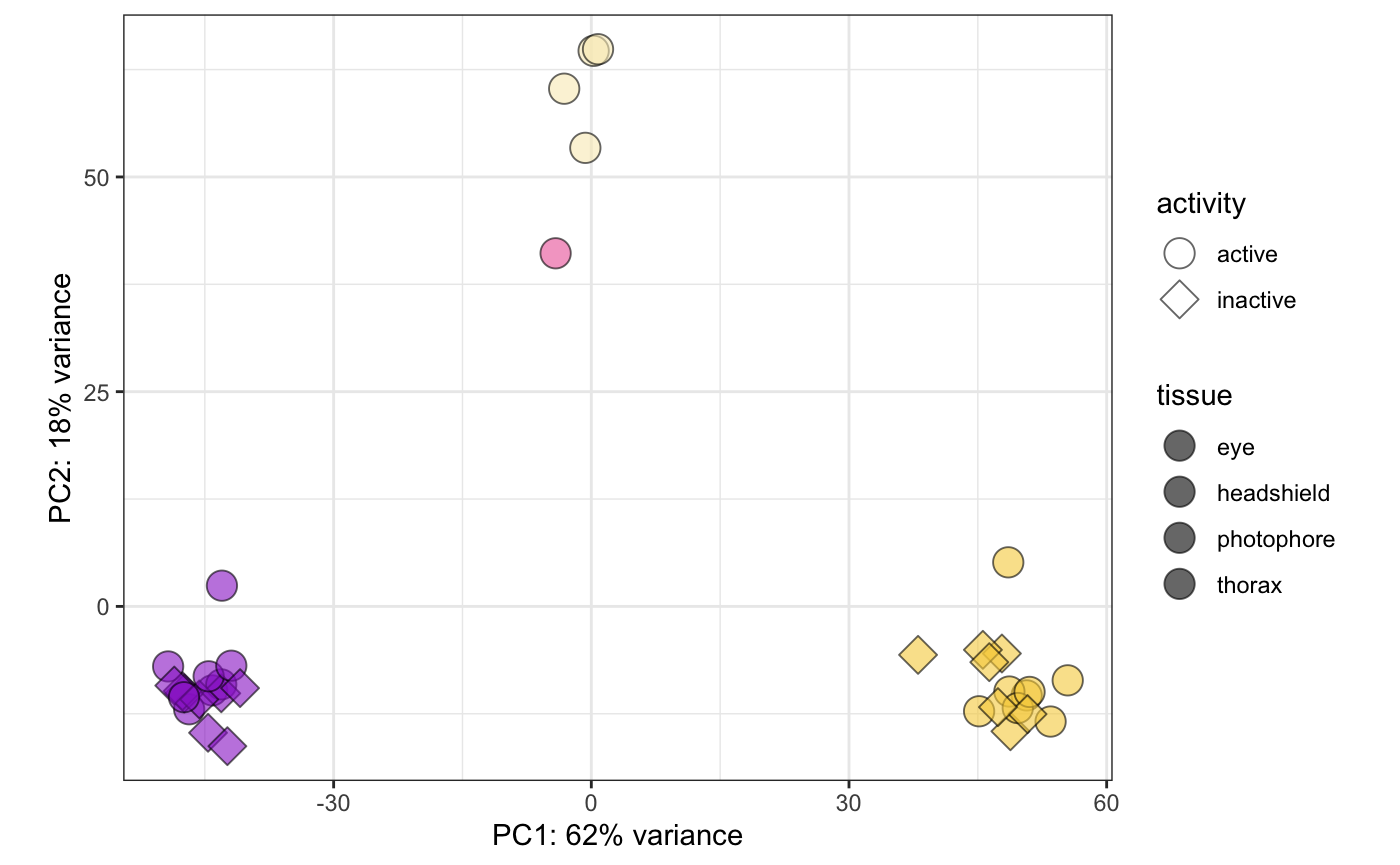


**Figure S7:** PCA of gene expression for all genes (total: 12,590 genes) using VST-transformed counts (VST transformation from DESeq2). Tissue type is the major factor driving gene expression differences in *Photinus pyralis*.


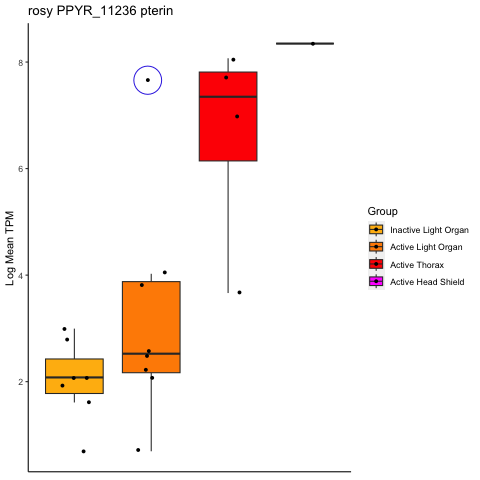


**Figure S8**. Expression of *rosy1*. There is a trend of higher expression of *rosy1* in active versus inactive light organs, but the statistical significance of this difference appears to be driven by one outlier sample (active green light organ, FS22A3) (blue circle).
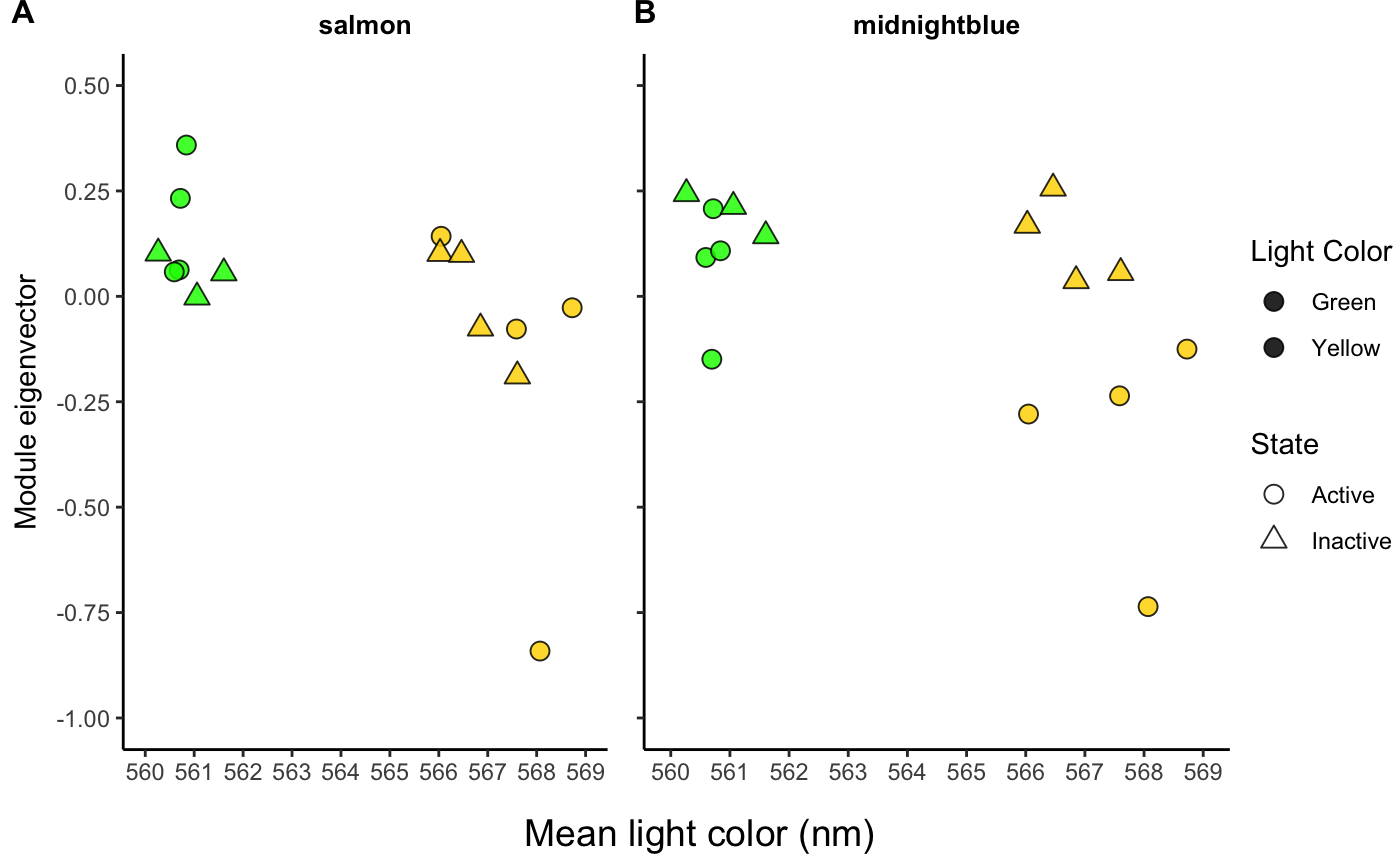


**Figure S9.** Modules associated with light color of *P. pyralis* light organs (p<0.05): M-30 (“salmon”) and M-20 (“midnightblue”). Shape corresponds to active (circle) and inactive (triangle) states. Light color is plotted on the x-axis as wavelength (nm), while samples are colored according to light color group (yellow or green). Module eigenvector, or PC1 of gene expression within the module, is plotted on the y-axis. Module gene expression varies within color groups, particularly among the active state of yellow.


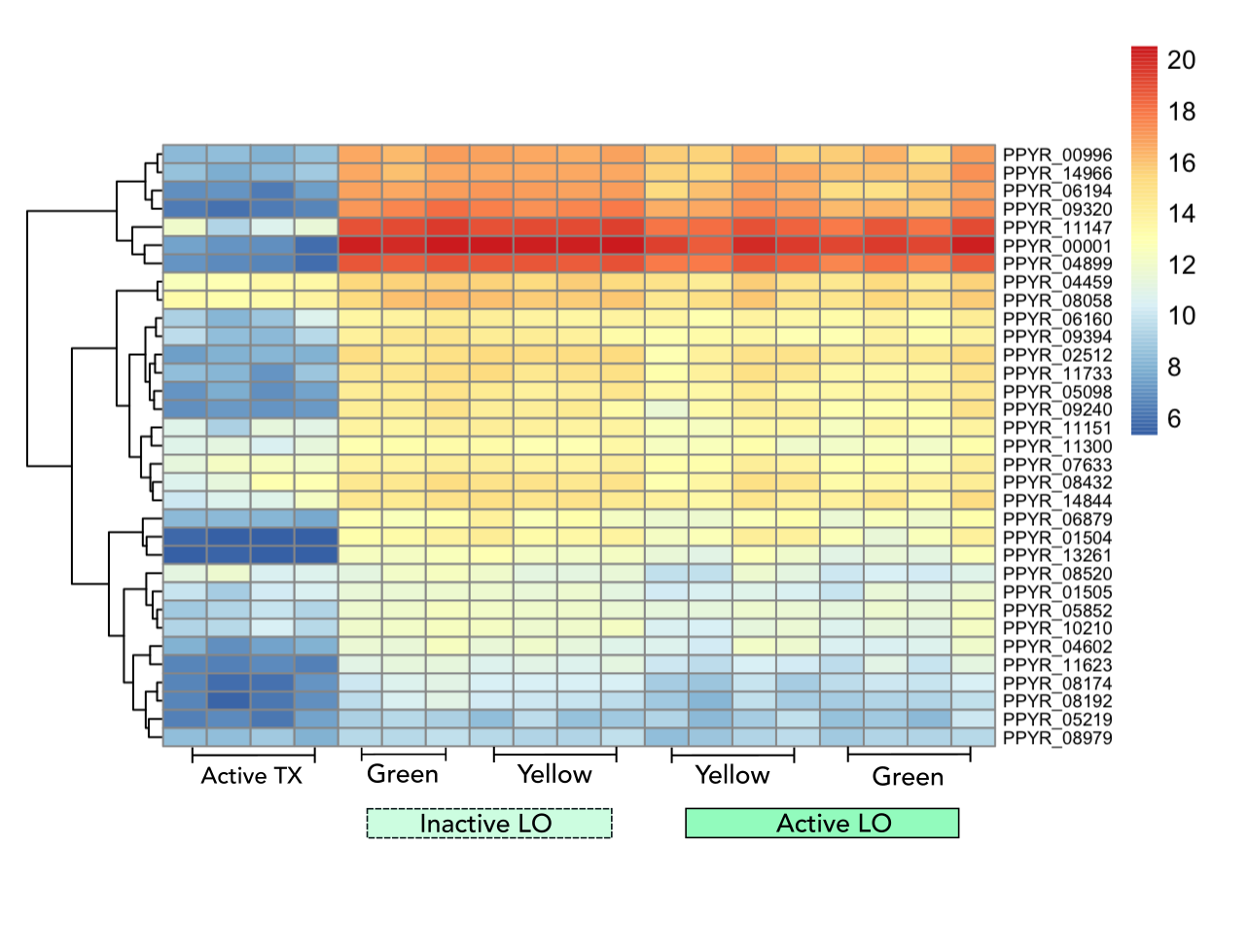


**Figure S10.** Module S-27 “Red” (N=546 genes) was marginally associated with inactive light organs (Table 2) and notably contained luciferase (PPYR_00001), a key enzyme in the bioluminescent reaction that was also a highly connected hub (Table S18). Overall, this module appears to contain an abundance of genes related to bioluminescence and may inform future studies on signaling physiology.


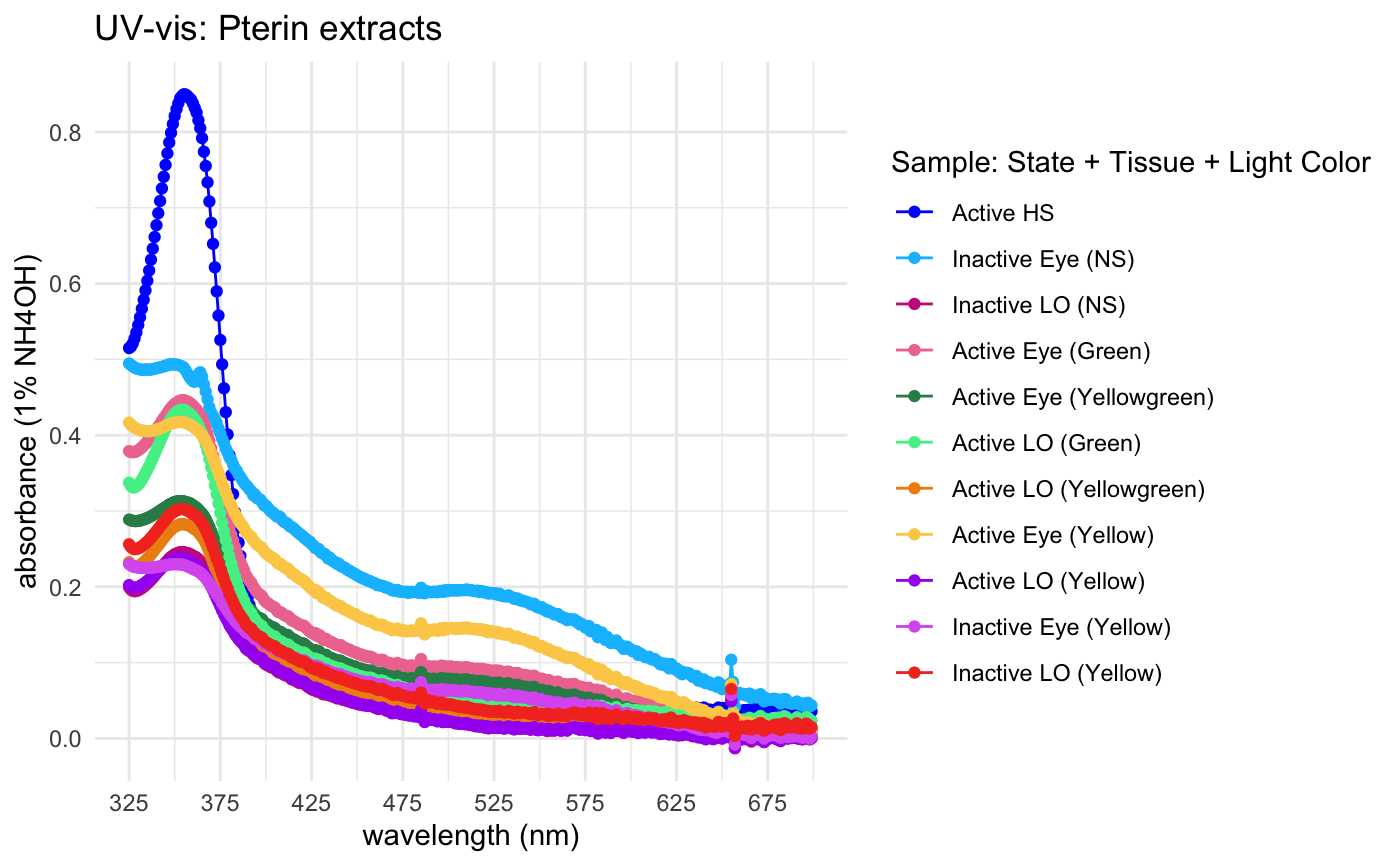


**Figure S11:** Absorbance measurements of pterin pigments from UV-vis. Blank was 1% NH_4_OH. HS=head shield, NS=no spectra (unknown light color).

**
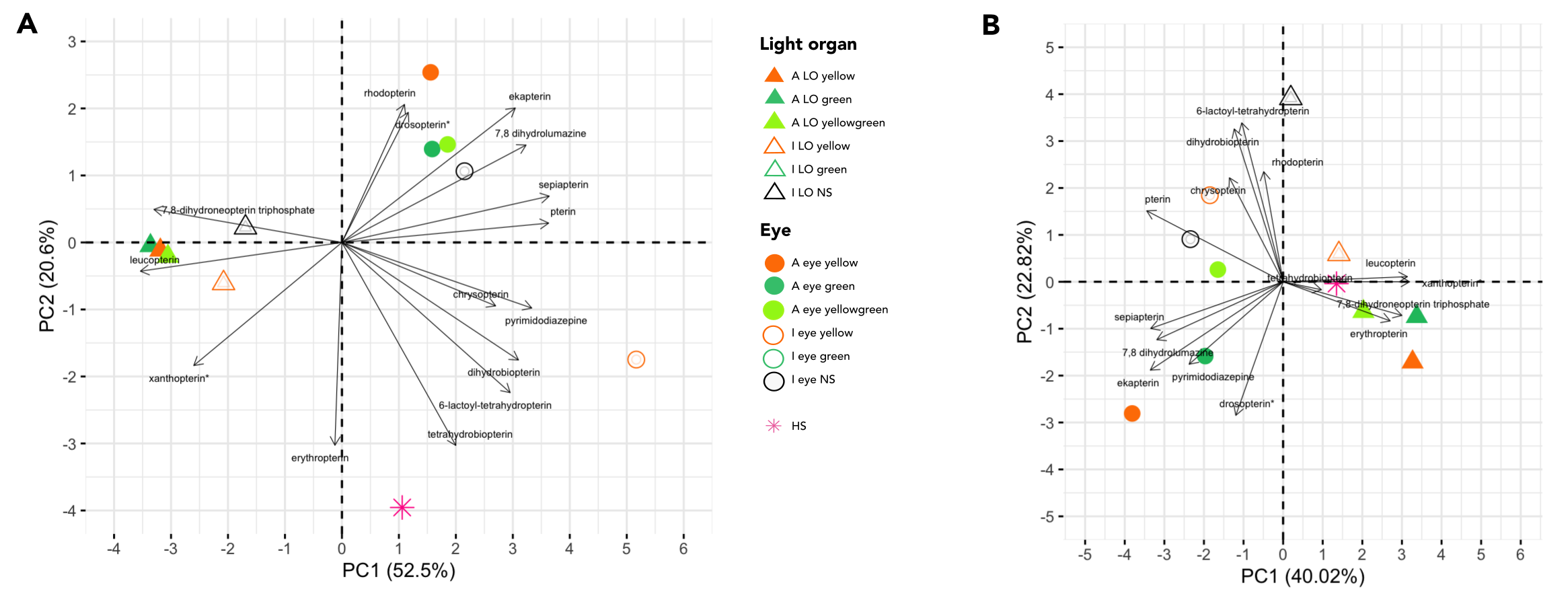
**

**Figure S12:** PCA Biplot of the 15 pterin substrates recovered in *P. pyralis* samples. Pooled samples (N=2) are represented as colored points (solid=active, open=inactive; triangle=light organ, circle=eye, star=head shield) and pterin substrates by vectors. Plot made with the fviz_pca_biplot function from R package factoextra v1.0.7.999 **Substrate has isoforms (i.e., same molecular weight): Lepidopterin/Rhodopterin/Pterorhodin, Xanthopterin/Isoxanthopterin, Drosopterin/Isodrosopterin.*


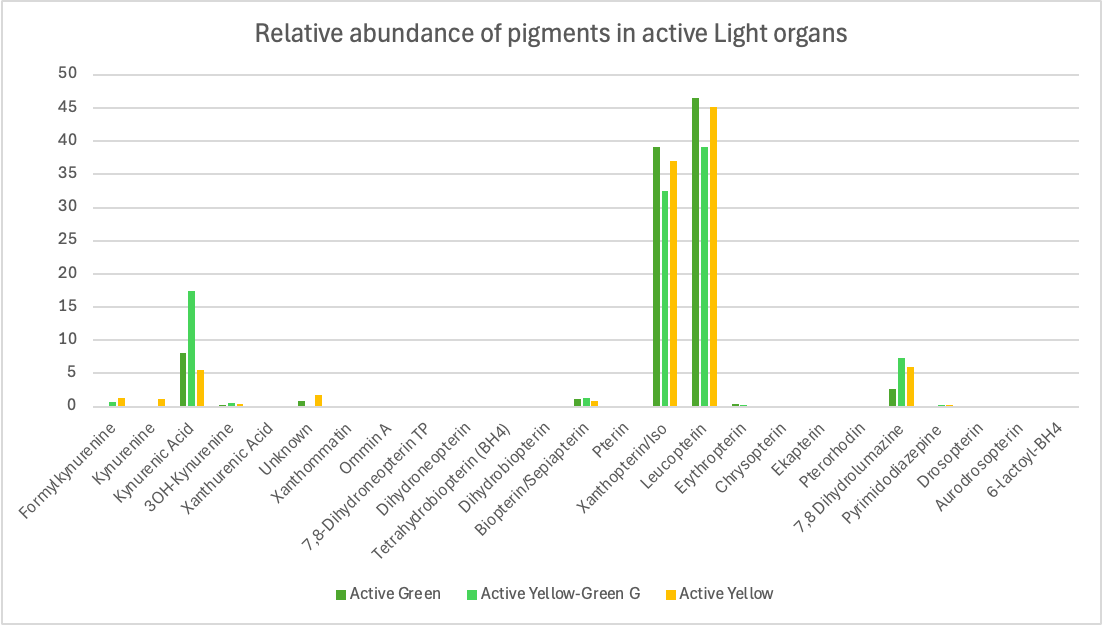


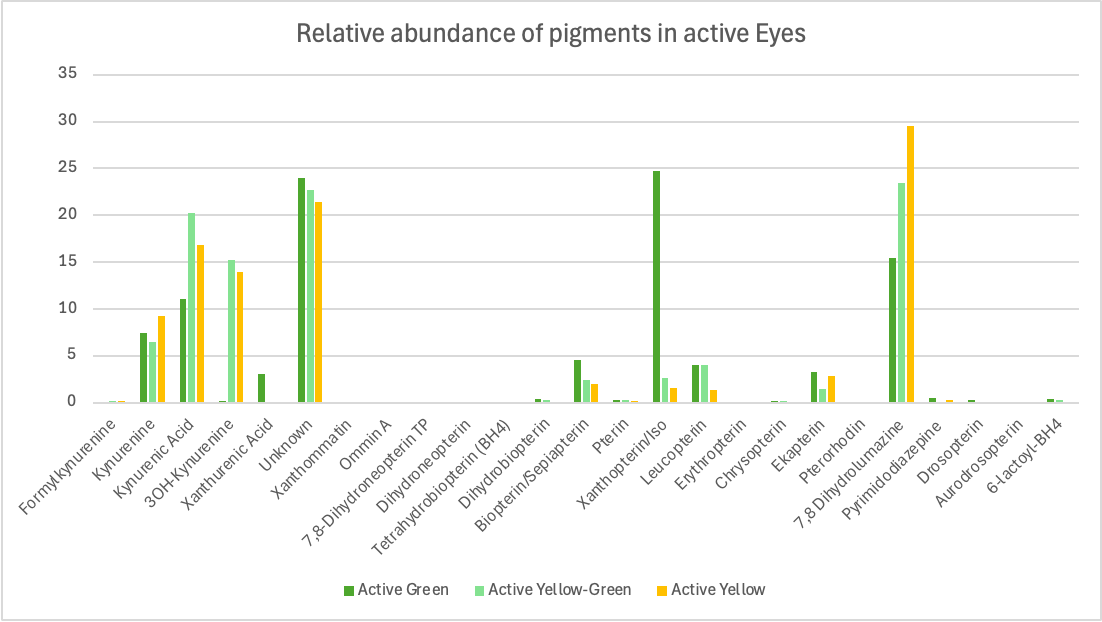


**Figure S13:** Pigments and their precursors from the ommochrome and pterin pathways in the LOs (top) and eyes (bottom) of active *P. pyralis* fireflies that emit green, yellow-green or yellow light color. Each sample represents pooled tissues from two fireflies. *3-hydroxy kynureninine is abbreviated 3OH-Kynurenine.*


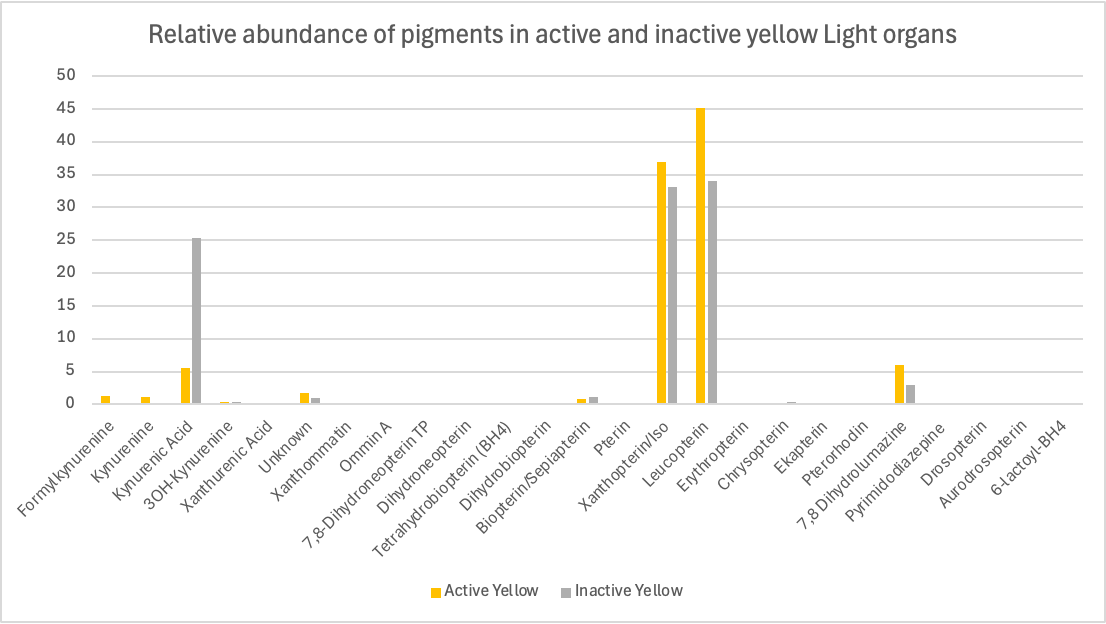


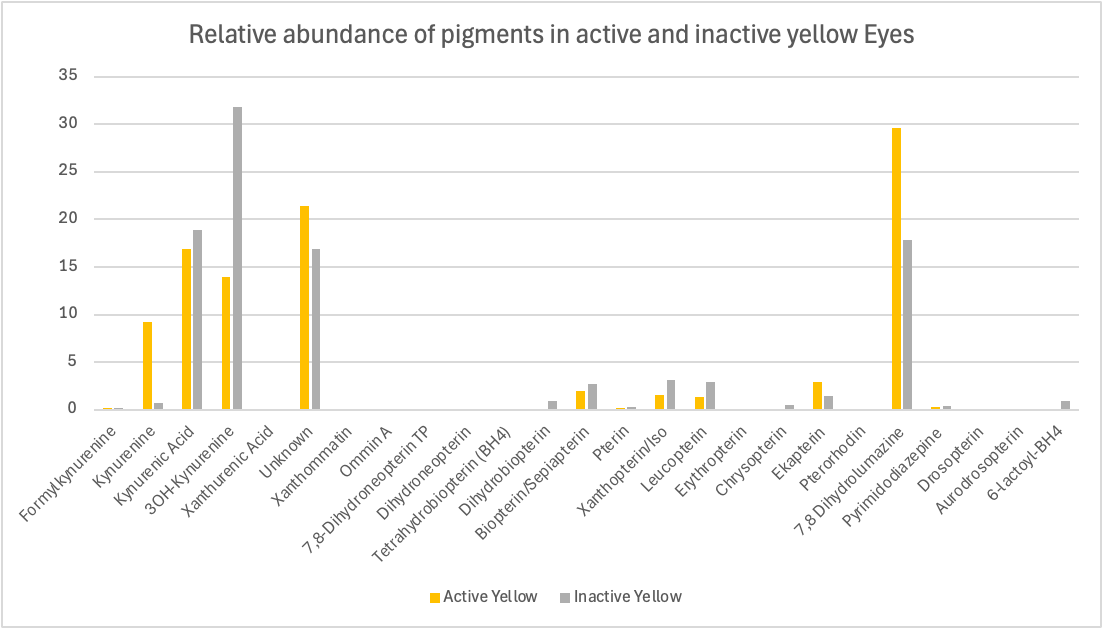


**Figure S14:** Relative pigment abundance in active and inactive yellow light organs (top) and eyes (bottom). Each sample represents pooled tissues from two fireflies. Kynurenic acid (off-white to light yellow) is much more abundant in inactive yellow firefly LOs than in active yellow LOs. Active yellow LOs tend to have both, slightly higher levels of kynurenine (light yellow) and 7,8 dihydrolumazine (yellow). *3-hydroxy kynureninine is abbreviated 3OH-Kynurenine.*


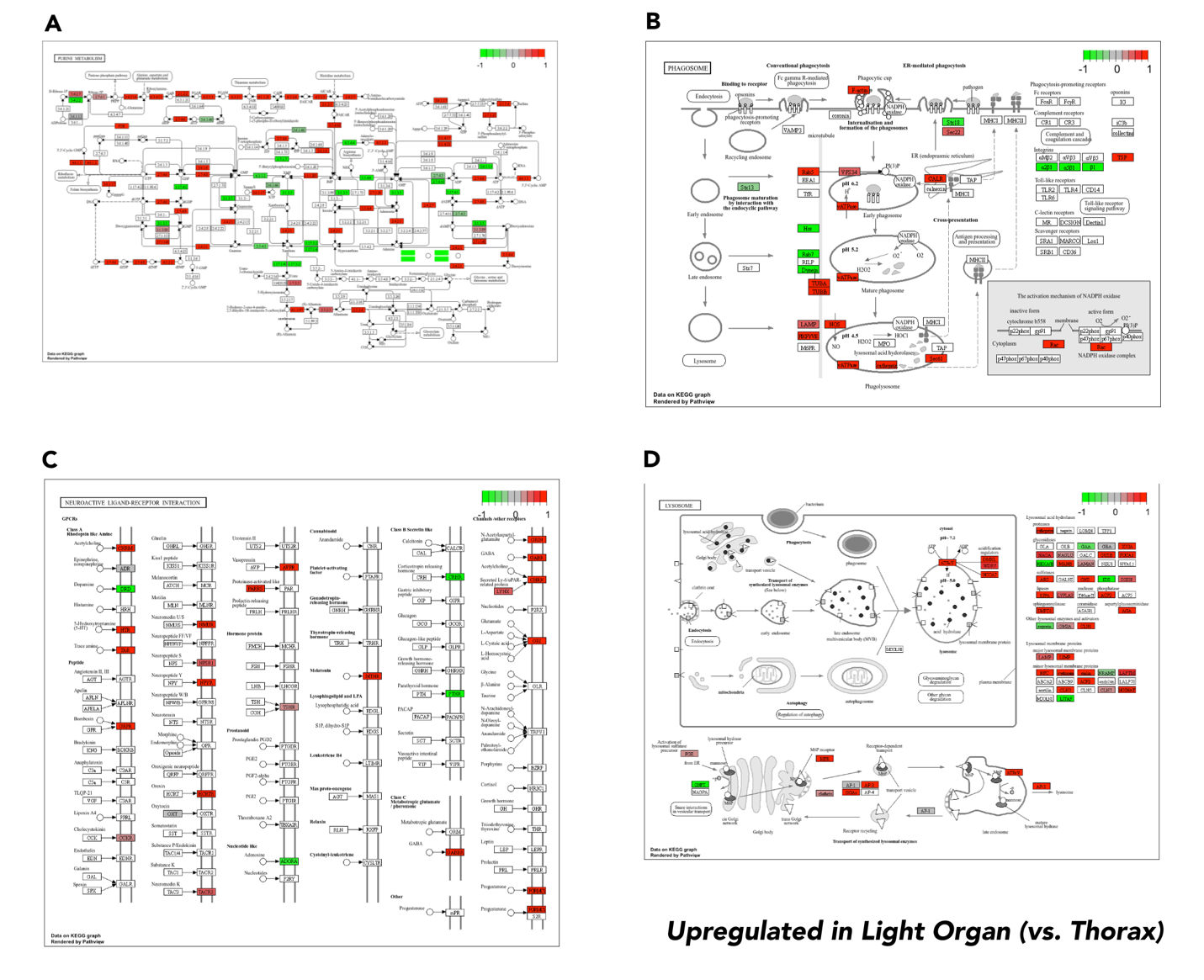


**Figure S15:** KEGG pathways “of interest” (q > 0.05, p < 0.05) enriched among genes upregulated in active LO relative to the thorax of active fireflies, suggesting these metabolic processes are relevant to the signaling LO. Pathway components (e.g., enzymes, non-enzymes) are displayed as boxes; labels correspond with KEGG orthology. Red=Upregulated in LO, Green=Downregulated in LO. A) Purine metabolism, B) Phagosome, C) Neuroactive Ligand-Receptor Interaction, and D) Lysosome. For complete list of KEGG pathways, see Table S8. Images created with Pathview v1.42.0 (Luo & [Brouwer](https://scholar.google.com/citations?user=cPpOd0kAAAAJ&hl=en&inst=2365059173406736517&oi=sra), 2013).

**
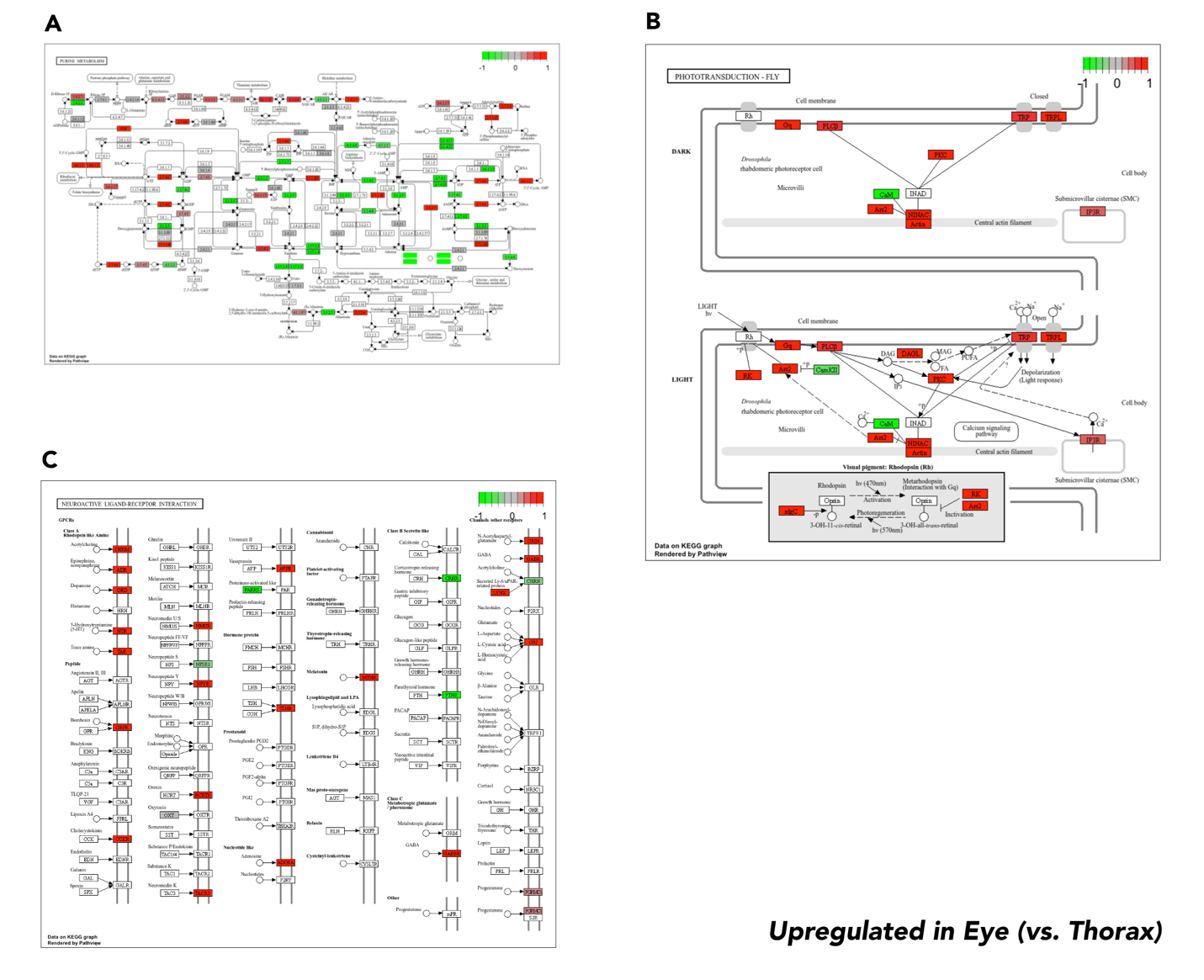
**

**Figure S16:** KEGG pathways “of interest” (q > 0.05, p < 0.05) enriched among genes upregulated in active eye relative to the thorax of active fireflies, suggesting these metabolic processes are relevant to the eye during the mate search. Pathways (i.e., purine metabolism, neuroactive ligand-receptor interaction) were upregulated in both photic tissues. Pathway components (e.g., enzymes, non-enzymes) are displayed as boxes; labels correspond with KEGG orthology. Red=Upregulated in eye, Green=Downregulated in eye. A) Purine metabolism, B) Phototransduction, and C) Neuroactive Receptor-Ligand Interaction. For complete list of KEGG pathways, see Table S8. Images created with Pathview v1.42.0 (Luo & [Brouwer](https://scholar.google.com/citations?user=cPpOd0kAAAAJ&hl=en&inst=2365059173406736517&oi=sra), 2013).


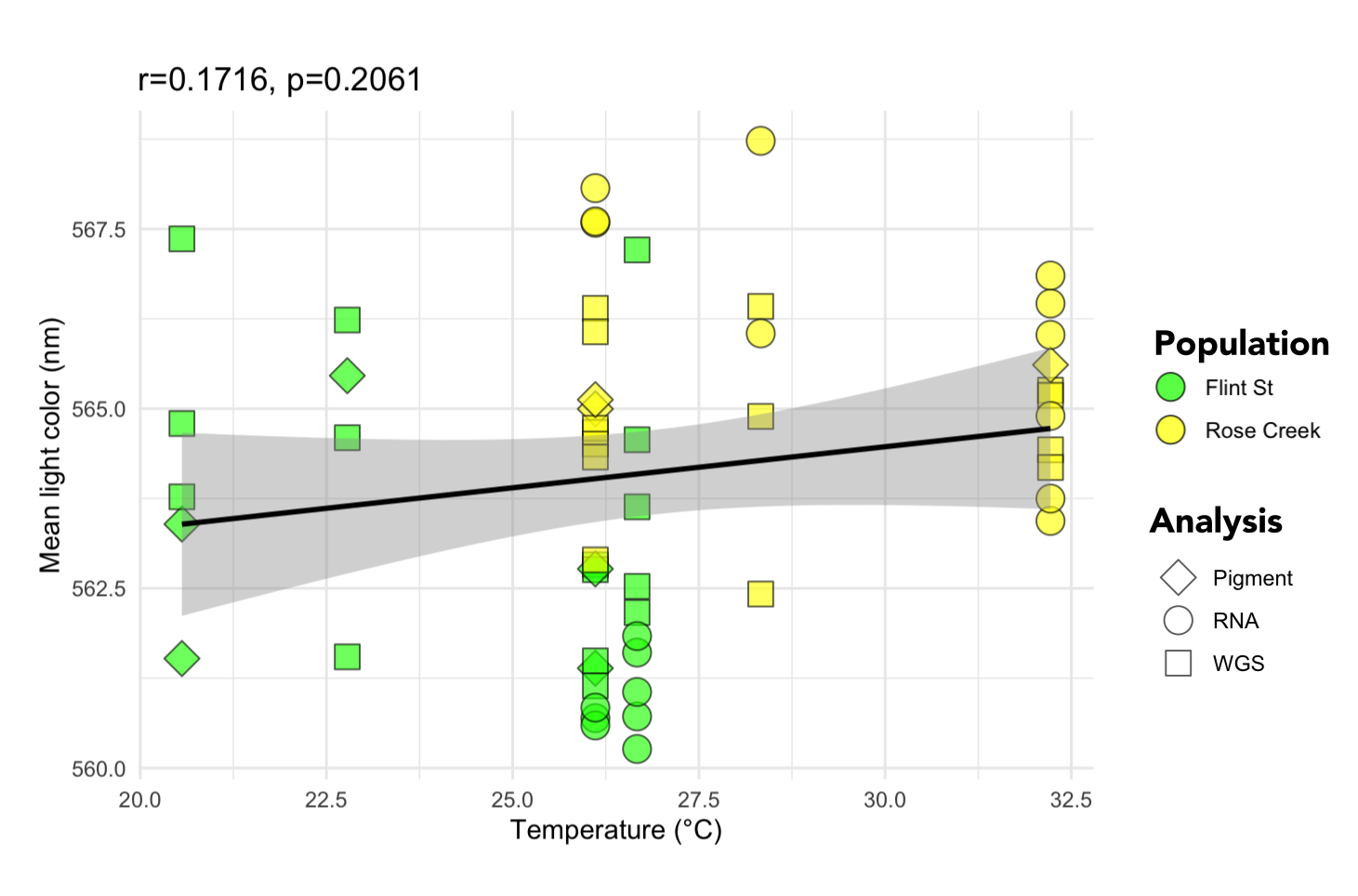


**Figure S17:** Light color was not significantly correlated with temperature (Pearson’s correlation: r= 0.1716, p-value= 0.2061) and the maximum temperature (32.22°C) was below the experimental threshold for red-shifted bioluminescence (34°C) observed in constrained *Sclerotia* fireflies Rabha et al. (2021). Overall, light color varied between individual fireflies and across temperatures. The temperature was higher during field days at RC when “yellower” fireflies were collected (26.11-32.22°C, mean=28.90679**±**2.753011°C) compared with our collection of “greener” fireflies at FS (20.56-26.67°C, mean=24.84321**±**2.462488°C). Temperature measurements obtained from Iowa Environmental Mesonet (recorded at the Athens Municipal station), which is in proximity to both Flint St (FS) and (RC) populations.


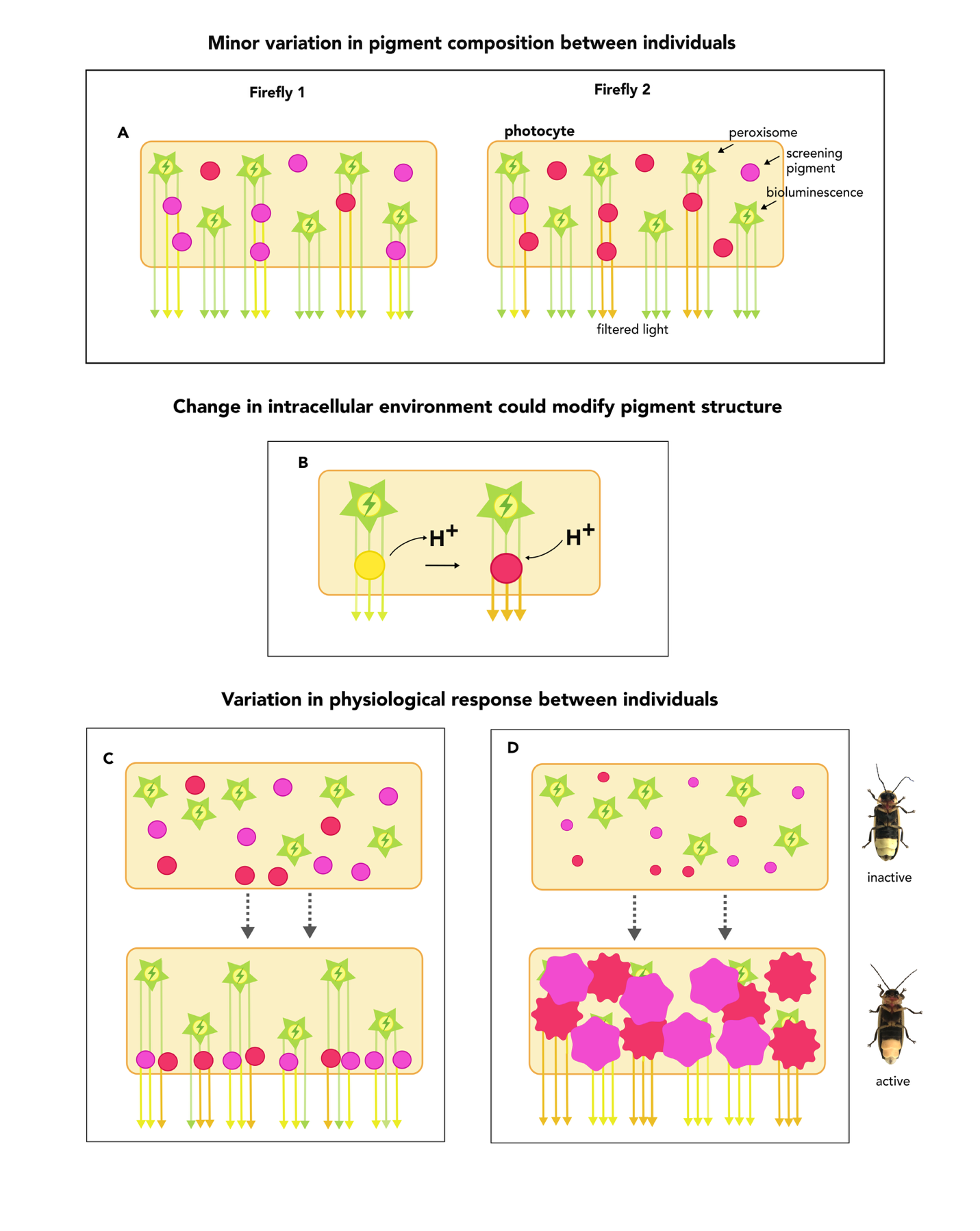


**Figure S18:** Genes encoding enzymes and transporters in pigment biosynthesis pathways (pigment genes) lacked differential expression between *P. pyralis* populations with greener and yellower light color. However, pigments could still contribute to intraspecific variation in light color, as described by the potential mechanisms: A) There are differences in the pigment composition (e.g., pigment type, abundance) across individual *P. pyralis* fireflies, as represented by color (magenta vs. red). Levels of pigment gene expression could vary within and between population to explain the measurable difference in light color; however, it may not have been substantial enough to detect significant changes in gene expression.

B) It is possible that modifications to the intracellular environment where pigments are located could alter its configuration (Figon & Casas, 2021). In this example, shifts in intracellular pH could alter the molecular structure of the pigment. Basic pH promotes the loss of a hydrogen (H^+^) whereas pigments in acidic conditions may gain a hydrogen. The pH directly influences redox potential and the likelihood of whether a molecule is oxidized (loss of electrons, hydrogen) or reduced (gain of electrons, hydrogen). Importantly, the redox state of a pigment determines what wavelengths it absorbs and reflects, resulting in a color change, as demonstrated by Futahashi et al. (2012). Binding to metals and proteins could also influence pigment properties (Figon & Casas, 2021). Therefore, the extent of these shifting intracellular conditions could contribute to differences in intraspecific light color. Genes differentially expressed between active light organs and active thorax (Contrast 2) were enriched for functions related to pH, raising the possibility this mechanism could influence light color variation.

Similarly, variation in physiological responses could influence light color across individual fireflies. C) Insect eyes use screening pigments to control the amount of light that enters the eye to stimulate the light-sensitive rhabdom. Under brightly lit conditions, pigments localize densely around the rhabdom to absorb indirect rays for enhanced vision. At night, they migrate away from the rhabdom to collect maximal light levels. We observed the *lightoid* gene was upregulated in both active light organs and eyes relative to thorax, which is notable because *lightoid* has been shown to associate with ommochrome and pterin pigments (Ma et al., 2004) and is involved with pigment migration in *Drosophila melanogaster* eyes (Satoh et al., 2008). Variation in the position of pigments within the light organ (i.e., clustered at periphery of photic cells vs. scattered) could have different light filtering effects across individuals, resulting in a range of emitted light color. However, it is unclear whether this mechanism could be present in tissues other than the eye and further study is needed.

D) In many animals (i.e., amphibians, fish, crustaceans), shifts in pigment conformation underlie color change. For example, the fiddler crab *Uca panacea* appears white at night, when its pigments are in a concentrated state, and black when dispersed during the day (e.g., Darnell, 2012). This dark coloration occurs because the dispersed pigment can absorb light. A comparable change in fireflies could underlie the light organ hue change between active and inactive states. The extent to which pigments exist in concentrated (upper) or dispersed (lower) states could affect their light absorption properties, which could also vary across individuals, resulting in light color variation. However, our study did not test this, and it is unknown whether a similar mechanism occurs in insects.

**4. Supplemental Tables**

***Located in Excel File: “Supplemental_Tables_Role_of_pigments_Photinus_pyralis_light_color.xlsx”***

**5.** **Supplemental References**

Capella-Gutiérrez S, Silla-Martínez JM, Gabaldón T. trimAl: a tool for automated alignment trimming in large-scale phylogenetic analyses. Bioinformatics. 2009 Aug 1;25(15):1972-3.

Darnell MZ. Ecological physiology of the circadian pigmentation rhythm in the fiddler crab Uca panacea. Journal of Experimental Marine Biology and Ecology. 2012 Sep 1;426:39-47.

Figon F, Casas J. The integrative biology of pigment organelles, a quantum chemical approach. Integrative and Comparative Biology. 2021 Oct;61(4):1490-501.

Futahashi R, Kurita R, Mano H, Fukatsu T. Redox alters yellow dragonflies into red. Proceedings of the National Academy of Sciences. 2012 Jul 31;109(31):12626-31.

Grabherr MG, Haas BJ, Yassour M, Levin JZ, Thompson DA, Amit I, Adiconis X, Fan L, Raychowdhury R, Zeng Q, Chen Z. Trinity: reconstructing a full-length transcriptome without a genome from RNA-Seq data. Nature biotechnology. 2011 Jul;29(7):644.

Haas BJ, Papanicolaou A, Yassour M, Grabherr M, Blood PD, Bowden J, Couger MB, Eccles D, Li BO, Lieber M, MacManes MD. De novo transcript sequence reconstruction from RNA-seq using the Trinity platform for reference generation and analysis. Nature protocols. 2013 Aug;8(8):1494-512.

Herzmann D, Arritt R, Todey D. Iowa environmental mesonet. Available at mesonet. agron. iastate. edu/request/coop/fe. phtml (verified 27 Sept. 2005). Iowa State Univ., Dep. of Agron., Ames, IA. 2004.

Kassambara A, Mundt F. Package ‘factoextra’. Extract and visualize the results of multivariate data analyses. 2017;76(2):10-8637.

Khan SA, Reichelt M, Heckel DG. Functional analysis of the ABCs of eye color in Helicoverpa armigera with CRISPR/Cas9-induced mutations. Scientific Reports. 2017 Jan 5;7(1):40025.

Kretzschmar D, Poeck B, Roth H, Ernst R, Keller A, Porsch M, Strauss R, Pflugfelder GO. Defective pigment granule biogenesis and aberrant behavior caused by mutations in the Drosophila AP-3β adaptin gene ruby. Genetics. 2000 May 1;155(1):213-23.

Luo W, Friedman MS, Shedden K, Hankenson KD, Woolf PJ. GAGE: generally applicable gene set enrichment for pathway analysis. BMC bioinformatics. 2009 Dec;10:1-7.

Luo W, Brouwer C. Pathview: an R/Bioconductor package for pathway-based data integration and visualization. Bioinformatics. 2013 Jul 15;29(14):1830-1.

Ma J, Plesken H, Treisman JE, Edelman-Novemsky I, Ren M. Lightoid and Claret: a rab GTPase and its putative guanine nucleotide exchange factor in biogenesis of Drosophila eye pigment granules. Proceedings of the National Academy of Sciences. 2004 Aug 10;101(32):11652-7.

Mirarab S, Nguyen N, Warnow T. PASTA: ultra-large multiple sequence alignment. InResearch in Computational Molecular Biology: 18th Annual International Conference, RECOMB 2014, Pittsburgh, PA, USA, April 2-5, 2014, Proceedings 18 2014 (pp. 177-191). Springer International Publishing.

Mullins C, Hartnell LM, Wassarman DA, Bonifacino JS. Defective expression of the μ3 subunit of the AP-3 adaptor complex in the Drosophila pigmentation mutant carmine. Molecular and General Genetics MGG. 1999 Nov;262:401-12.

Mullins C, Hartnell LM, Bonifacino JS. Distinct requirements for the AP-3 adaptor complex in pigment granule and synaptic vesicle biogenesis in Drosophila melanogaster. Molecular and General Genetics MGG. 2000 Aug;263:1003-14.

Nguyen LT, Schmidt HA, Von Haeseler A, Minh BQ. IQ-TREE: a fast and effective stochastic algorithm for estimating maximum-likelihood phylogenies. Molecular biology and evolution. 2015 Jan 1;32(1):268-74.

Oba Y, Ojika M, Inouye S. Firefly luciferase is a bifunctional enzyme: ATP-dependent monooxygenase and a long chain fatty acyl-CoA synthetase. FEBS letters. 2003 Apr 10;540(1-3):251-4.

Rabha MM, Sharma U, Barua AG. Light from a firefly at temperatures considerably higher and lower than normal. Scientific Reports. 2021 Jun 14;11(1):12498.

Satoh AK, Li BX, Xia H, Ready DF. Calcium-activated Myosin V closes the Drosophila pupil. Current Biology. 2008 Jul 8;18(13):951-5.

Simpson F, Peden AA, Christopoulou L, Robinson MS. Characterization of the adaptor-related protein complex, AP-3. The Journal of cell biology. 1997 May 19;137(4):835-45.

Vogels GV, Van der Drift C. Degradation of purines and pyrimidines by microorganisms. Bacteriological reviews. 1976 Jun;40(2):403-68.
